# Alternative Excipients for Protein Stabilization in Protein Therapeutics: Overcoming the Limitations of Polysorbates

**DOI:** 10.3390/pharmaceutics14122575

**Published:** 2022-11-23

**Authors:** Angel J. Castañeda Ruiz, Maryam A. Shetab Boushehri, Tamara Phan, Stefan Carle, Patrick Garidel, Julia Buske, Alf Lamprecht

**Affiliations:** 1 Department of Pharmaceutical Technology and Biopharmaceutics, University of Bonn, 53121 Bonn, Germany; 2 Boehringer Ingelheim Pharma GmbH & Co. KG, Innovation Unit, PDB, Birkendorfer Straße 65, 88397 Biberach an der Riss, Germany

**Keywords:** polysorbate alternatives, excipient, surfactant, protein stabilization, protein biotherapeutic formulations

## Abstract

Given their safety and efficiency in protecting protein integrity, polysorbates (PSs) have been the most widely used excipients for the stabilization of protein therapeutics for years. In recent decades, however, there have been numerous reports about visible or sub-visible particles in PS-containing biotherapeutic products, which is a major quality concern for parenteral drugs. Alternative excipients that are safe for parenteral administration, efficient in protecting different protein drugs against various stress conditions, effective in protein stabilization in high-concentrated liquid formulations, stable under the storage conditions for the duration of the product’s shelf-life, and compatible with other formulation components and the primary packaging are highly sought after. The aim of this paper is to review potential alternative excipients from different families, including surfactants, carbohydrate- and amino acid-based excipients, synthetic amphiphilic polymers, and ionic liquids that enable protein stabilization. For each category, important characteristics such as the ability to stabilize proteins against thermal and mechanical stresses, current knowledge related to the safety profile for parenteral administration, potential interactions with other formulation components, and primary packaging are debated. Based on the provided information and the detailed discussion thereof, this paper may pave the way for the identification or development of efficient excipients for biotherapeutic protein stabilization.

## 1. Introduction

The term “biotherapeutics” encompasses a wide range of biological products used with the prime objective of treating various human diseases. Unlike small molecular drugs, however, the production of biotherapeutics, at least in part, requires the use of a living host, capable of producing molecules with greater multidimensional complexity with secondary, tertiary, or even quaternary conformations [1]. Biotherapeutics comprise a broad range of biologically-derived therapeutics [2]. Among these, protein therapeutics are the most extensively developed group and form a major part of the FDA-approved biotherapeutics. Protein therapeutics consist of diverse subclasses such as antibodies (Ab), Fc-fusion proteins, blood factors and anticoagulants, enzymes, growth factors, protein hormones, cytokines, thrombolytics, scaffold proteins, etc. These are often used to replace deficient or abnormal proteins, promote or inhibit various cellular pathways, exert a new and non-existing function, interfere with a molecule of interest, or deliver various cargos to specific targets [3]. While the nature and the purpose of protein therapeutics is quite diverse, monoclonal Abs (mAb) remain the most prevalent subcategory in terms of clinical application.

In general, the development of protein therapeutics is a complex multiple step process, during which maintaining the protein integrity from the purification up to the administration of the final product to the patient is fraught with numerous and diverse challenges. Given their complex higher order conformational structures as well as the presence of various functional groups, protein biotherapeutics are susceptible not only to chemical degradation, but also to physical-induced conformational changes. The chemical instabilities are related to the breakage and/or formation of covalent bonds in the protein’s first-order structure, generated by intramolecular modifications such as non-reducible cross-linking [4,5], deamidation [4,6,7,8,9], formation of basic or acidic species [10,11], glycation (Maillard reaction) [12], isomerization [6,13] oxidation [4,11], fragmentation [10,14], C-terminal clipping [15], reduction [16], hydrolysis [17], and racemization [18]. Physical-induced conformational changes, on the other hand, are often in the form of denaturation [19,20], aggregation [21,22,23,24,25], surface adsorption [26], precipitation and unfolding [27], often impacting the secondary or tertiary structures of the proteins [28]. The challenges associated with maintaining the functionality and integrity of the protein drugs become more pronounced in the case of mAb-based therapeutics, as they often require higher dose concentrations (usually 50–200 mg/mL). The development of such high concentration liquid formulations (HCLF) comes along with additional challenges in terms of protein solubility and hydration, colloidal and conformational stability, and solution properties [29,30,31,32], which are directly related to the formation of mAb particles [33,34]. With increasing protein concentration of the biotherapeutic, viscosity and opalescence of the formulation also rises, and liquid–liquid phase separation phenomenon becomes more likely to occur [35]. While the liquid–liquid phase separation does not impact the native protein structure per se, it promotes reversible or irreversible protein particle formation, which can result in a reduction of the therapeutic efficiency and trigger immune reactions upon administration [36]. Furthermore, exposure to various interfaces (air–liquid, solid–liquid, and liquid–liquid) during different stages of manufacturing, including freeze–thawing, pumping, sterile filtration, lyophilization, and fill and finish processes, as well as various post-production stages such as storage in the primary packaging and transportation, can promote conformational changes leading to protein denaturation [37,38,39,40,41,42]. When inadequately formulated, proteins are adsorbed to such interfaces by virtue of their amphiphilic properties, and thus undergo conformational changes to reduce the interfacial tension, or else create viscoelastic interfacial protein gels. While the former can act as a nucleating point for further protein particle formation, the latter can facilitate agitation-mediated formation of protein particles [43,44]. The formation of such protein particles can in turn affect the quality of the final product and lead to undesirable consequences such as irreversible loss of activity, increase of the visible or sub-visible particle levels beyond the permitted threshold, and potential immunogenicity [45,46,47,48]. Hence, preserving the integrity of the protein drug from all potential sources of stress detailed above is not only relevant but of vital importance.

While factors such as harsh temperatures and extreme acidity or alkalinity can be more easily avoided, stress-induced physicochemical protein instability due to, e.g., the manufacturing is often inevitable. High protein concentrations are sometimes indispensable to achieve a desired therapeutic outcome, and exposure to various interfaces within the above-mentioned stages is unavoidable [17,49,50]. Hence, protein formulations require additional protection from the inevitable exposure to interfaces to ensure their stability during the product’s shelf-life (at least two years at 2–8 °C).

To ensure the integrity of the therapeutic protein in the final formulation, typically tonicity adjusting agents are added, which are not the focus of this review, as well as surface active stabilizers, like surfactants. After decades of research and production of therapeutic formulations, the excipient selection is frequently based on the approved commercialized formulations [51]. The use of excipients plays a considerable role in formulation development since the nature of the excipient influences directly or indirectly the stabilization of the protein. For a proper selection of the excipient for a specific parenteral protein formulation, several selection criteria must be considered [52,53]. These include:

1. The excipient’s effect on the overall quality, stability, and effectiveness of the drug product;
2. Physical, chemical, and biological compatibility of excipient with the drug as well as the packaging system [54];
3. Compatibility of the excipient with the manufacturing process;
4. Amount of excipients that can be added to drug product, both from the formulation, safety and toxicological perspectives.

Excipients such as surfactants are ubiquitously employed in the formulation to stabilize protein biotherapeutics. This is primarily through the preferential adsorption of such surfactants to the interfaces, sparing the protein drug from interface-mediated unfolding and denaturation. The second possible mechanism involves the formation of protein–surfactant complexes, which in turn minimizes the interaction of the protein with other protein molecules and the interfaces [55,56]. The latter mechanism, however, seems to be only applicable to certain types of proteins and is not possible in formulations with the routinely used protein to polysorbate ratios [56,57]. As the third mechanism, some surfactants have been shown to promote protein folding through a chemical chaperone mechanism [57].

Within the context of protein stabilization, both ionic and non-ionic surfactants have been proposed. Ionic surfactants such as sodium dodecyl benzenesulfonate and sodium dodecyl sulfate might offer the benefit of a possible strong interaction with the charged protein molecules in addition to adsorption to the interfaces [58]. Notwithstanding, non-ionic surfactants are more frequently used for the stabilization of parenteral protein formulations, as they have a proven history of low toxicity, minimal reactivity, and good tolerability for clinical use. On top of that, they are associated with the added benefit that some have been already approved by the regulatory bodies for use in various medicinal products [59,60,61].

**Figure 1:**
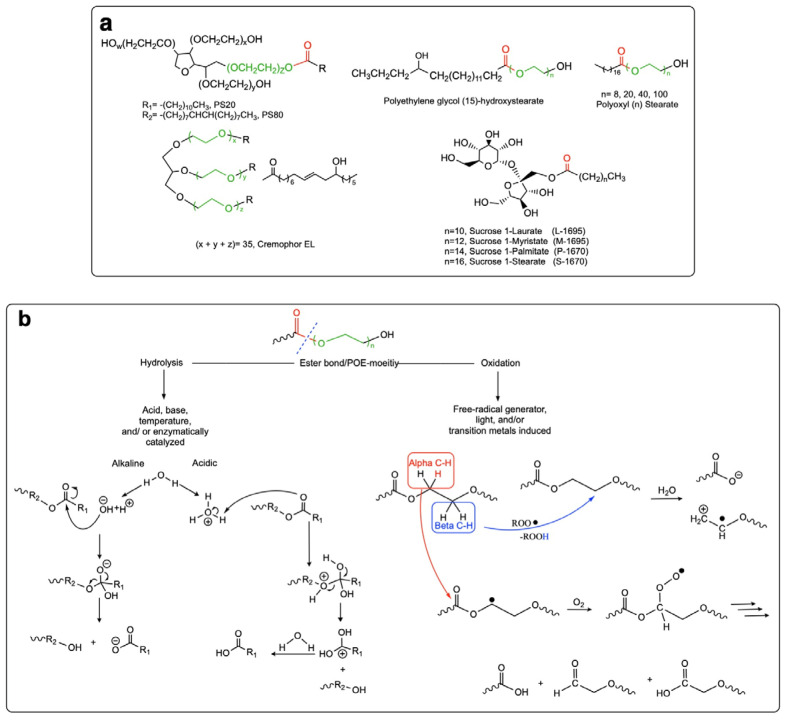
(**a**) Idealized chemical structure of PSs and potential surfactant alternatives containing ester bonds. The ester bonds are highlighted in red, and the POE moiety in green; (**b**) Expected degradation mechanisms for ester bond/POE-containing surfactants [65].

Polysorbates (PSs) are the most prevalently used family of non-ionic surfactants for protein stabilization (Figure 1a). Various types of PSs, including PS20 (Tween^®^ 20) and PS80 (Tween^®^ 80), have been shown not only to prevent protein particle formation as a result of exposure to liquid–air interfaces and freeze–thawing stresses, but also to minimize the protein adsorption to various liquid–solid interfaces such as the sterilization filters as well as the primary packaging. PS20 also prevents mechanical stress-induced particle formation in the case of several protein types [43,62,63,64]. To achieve such desirable stabilizing effects, PS20 and PS80 are often used in the formulation of the protein drugs in a concentration range of 0.003–3 mg/mL based on the Physicians Desk Reference [55]. Table S1 provides an overview of the FDA and CBER (Center for Biologics Evaluation and Research)-approved protein biotherapeutics stabilized with PSs, also indicating the concentration of the surfactant.

Notwithstanding the offered benefits, PS surfactants are associated with the drawback of chemical instability [66,67,68,69] because of the hydrolysis of the ester bond, auto-oxidation, and cleavage of the ethylene oxide subunit [4,68,70,71,72,73,74,75,76,77,78,79,80].

In line with such limitations, the current paper seeks to review potential alternative excipients to PSs for the stabilization of the parenteral protein formulations. We will start by presenting the latest findings regarding the benefits and limitations of the PSs in the formulation of protein biotherapeutics. We will then provide a discussion of FDA-approved surfactants for food and cosmetic applications that, given their chemical and physical similarity to PSs, could be tested for the preservation of parenteral protein therapeutics. Finally, we will highlight other alternative, mostly non-surfactant, excipients of approved or non-approved regulatory status that can be used to achieve protein stabilization without the drawbacks associated with the PS surfactants. In the end, it must be noted that most of the excipient classes discussed in paper, along with other inactive ingredients commonly used in the formulation of protein bio therapeutics, are well-established, and a thorough introduction of all is out of the scope of this paper. For more information, the reader is referred to several review articles that elaborate elegantly on the subject [81,82].

## 2. The Benefits and Drawbacks of Polysorbates in Protein Stabilization

PSs have been shown to protect protein therapeutics against denaturation or particle formation during various stages of the formulation and manufacturing, such as freeze–thawing [63], surface-induced agitation [83], lyophilization [84], and spray-drying [85]. They have also been reported to protect the protein during storage, e.g., in prefilled syringes [86]. Although showing stabilizing properties, the underlying mechanism(s) of PS protecting protein integrity remains unclear. Different mechanisms have been proposed, including the preferential adsorption to the interfaces and inhibition of protein–protein interactions, leading to a reduction of the protein unfolding and protein particle formation [56,87]. The latter has been discussed both to the competitive binding of the PSs to the hydrophobic regions of the proteins [55], as well as the chemical chaperone effect [88], either as a result of protein hydration [89,90], or else the reduction of the protein‘s interfacial affinity due to the surfactant‘s blocking properties [87,91]. Finally, Garidel et al. (2021) showed the formation of protein–PS micelle complexes to be unlikely at the routinely used protein to polysorbate ratio [56].

PSs are complex heterogenic mixtures of sorbitan polyoxyethylene (POE) fatty acid esters and derivatives thereof, including POE, POE sorbitan, POE esters, sorbitan, sorbitan esters, isosorbide, and isosorbide POE fatty acid ester [92,93,94,95,96,97]. European Pharmacopoeia (Ph. Eur.) defines PSs as mixtures of partial esters of fatty acids with sorbitol and its anhydrides, ethoxylated with approximately 20 moles of ethylene oxide for each mole of sorbitol and sorbitol anhydrides [98,99]. Different types of PSs contain varying types of fatty acids esterified with the POE sorbitan. For example, in the case of PS20, lauric acid comprises 40–60% of its total fatty acid content [99], whereas at least 58% of the fatty acid content in PS80 should be oleic acid [98]. As the PS chemical synthesis employs raw natural materials, the fatty acid content of the final product is rather heterogeneous [97,100,101,102]. According to the Ph. Eur., the composition of PS20 and PS80 is stipulated to contain at least 9 and 7 different types of fatty acids, respectively (see Table 1) [98,99]. Furthermore, the varying amounts of POE, sorbitan POE and isosorbide POE fatty acid esters in the final PS product renders them a diversified pool of subspecies [102]. While other subspecies of PSs are also available in the market, a detailed discussion of the structural properties of all PS subspecies is beyond the scope of the current paper.

Despite the enumerated benefits, the limited stability of the PSs constitutes the main drawback for their use in the formulation of protein therapeutics [79,80]. Early data obtained by Donbrow et al. (1978) highlighted the instability of various types of PSs upon storage in aqueous solutions, for these were associated with an increase of peroxide content, continuous raise of acidity, decrease in the surface tension, and a drop in the cloud point [73]. The degradation process of PSs, as well as the underlying mechanisms, needed to be further investigated for two reasons. First and foremost, the degradation-mediated reduction of PS concentration could increase the risk of protein particle formation over time, and second, the impact of PS side-products upon the protein stability needed to be explored [70,103,104]. Today, it is well-known that PSs undergo degradation mainly through the enzymatic-induced hydrolysis of the fatty acid ester bonds [105] and auto-oxidation [68,76,104,106,107] (see Figure 1b).

The hydrolytic degradation process for PSs is characterized by the cleavage of the ester bond between the fatty acid moiety and the POE chain, releasing POE, POE sorbitan, or POE isosorbide, as well as free fatty acids (FFAs) as major degradation products. The chemical hydrolysis of these surfactants can be catalyzed both by acidic and alkaline pHs [65], and is accelerated at high temperatures (e.g., 40 °C) [94]. The acid-catalyzed reaction is thermodynamically favored by an SN2 (nucleophilic substitution type 2) mechanism, where an ester carbonyl is attacked by a molecule of H_2_O under extremely acidic conditions (pH < 3) [105,108]. Hence, under normal pharmaceutical relevant conditions (slightly acidic to neutral pH of the protein biopharmaceuticals (pH 5–7) [70], and storage and administration temperatures of 2–8 °C and 25 °C, respectively), the chemical hydrolysis of the incorporated PSs is negligible. The hydrolysis of PSs, however, has also been shown to be enzyme-mediated [74,109,110,111,112,113,114]. This degradation route is the main cause of PS hydrolysis in highly concentrated monoclonal antibody (mAb) formulations [115,116]. Enzymatic hydrolysis of PSs is often induced by the host cell protein (HCP) traces, also referred to as PS-degradation enzymes, which, despite the best efforts for their complete removal, often linger in very small amounts in the purified therapeutic protein [117,118,119]. HCPs such as lipoprotein lipase [117,120], lysosomal acid lipase (LIPA) [120,121], phospholipases (e.g., phospholipase A2 group VII (PLA2G7) [122], and group XV lysosomal phospholipase A2 isomer X1 [111]), palmitoyl-protein thioesterase 1 (PPT1) [120], carboxylesterases [123], carboxylester hydrolases [112], and sialate O-acetylesterases [124] have been indeed shown to promote the enzymatic degradation of the PS in protein formulations. Due to their low amounts, residual HCPs are difficult to detect, and their measurement and control in formulations are highly challenging [125]. Furthermore, analytical methods detecting lipase activity vary from lipase to lipase and might thus lead to false positive results. For instance, phospholipase B-like 2 (PLBD2) was first proposed as a residual HCP responsible for PS degradation [109]. However, recent studies showed that depletion of PLBD2 in host cells did not diminish the degradation of PSs [126]. Hence, approaches that measure the lipase activity should be further supported by PS-based monitoring analyses, e.g., fluorescence micelle assay and reverse-phase high performance liquid chromatography (RP-HPLC) to provide conclusive evidence about the PS degradation potentials of certain HCPs [127]. The reduction of the PS concentration and the precipitation of FFA particles as a result of the enzymatic hydrolysis mediated by different HCP traces have been demonstrated in various studies [74,109,111,122,128,129]. Concurring with these findings, protein concentration has been shown to significantly influence the PS degradation rate, as this will dictate the amount of the HCP residues in the product [130]. Furthermore, purification of mAbs from lipoprotein lipase knockout Chinese hamster ovarian cells has been shown to account for significantly reduced degradation of PS20 and PS80 in comparison with those purified from the non-modified hosts [117]. Although HCPs are co-purified with the active pharmaceutical ingredient (API), not all HCPs are active or degrade PSs specifically. In the research of Graf et al. (2021), the identification of HCPs in a mAb solution gave 60 different HCPs, of which 23 have different catalytic activities, and 3 of them act upon PSs [120]. Similarly, in the study published by Dixit et al. (2016) [109], ten HCPs were identified in a sulfatase drug, however, not all identified HCPs were ester hydrolases. The work by Zhang et al. (2021) [124] also presents a screening of HCPs in seven different mAb drug candidates (mAb 1–7), identifying 30 HCPs from which a few proteins have potential lipase activity. Among these proteins, SIAE (sialate-O-acetylase) strongly enzymatically degrades PS20 [124]. One of the latest reviews focusing on PS enzymatic degradability lists 12 HCPs, including ceramidase, lipases, and esterase, which are responsible for the enzymatic degradation of PS20 [80]. Based on these reports, it can be concluded that the type of HCPs, and the amount thereof, present in biologic parenteral formulations varies depending on the type of the API they have been co-purified with and the purification process. Furthermore, the activity of enzymes depends on the temperature and pH value of the formulation buffer. As a result, some formulations might be more prone to HCP-mediated enzymatic degradation of the PS when compared to others.

Adjunct to chemical and enzymatic hydrolysis, auto-oxidation is a further cause of PS degradation in protein formulations. The reaction starts with a free radical initiation [130], which is favored by traces of peroxide species and transition state metals (e.g., iron, copper), as well as the presence of oxygen in contact with light, and is followed by propagation and termination stages [70,73,107,116] (see Figure 1b). In reaction phase I, a carbon-based radical is formed in either α or β carbon on the POE chain [71]. In reaction phase II, a reaction of the carbon radical with oxygen forms an organic peroxide, from which a hydrogen atom is abstracted, and a carboxylic acid and new carbon radical are formed allowing for the propagation reaction. In reaction phase III, the formation of bimolecular interactions through radicals provides the quenching of the auto-oxidation process [73,106]. The principal degradation products of auto-oxidation are hydroperoxides generated from the oxidation of the ethylene oxide moieties (of the PS20 and PS80) and short-chain POE esters (in case of all PSs). These, in turn, degrade and lead to the formation of secondary products that include acids, peroxides, alkenes, aldehydes, ketones, furanones, alcohols, FFAs, and POE esters [71,73,78,130,131,132], products that may oxidize the therapeutic protein under long term storage [67,74]. Intermediate radicals formed in reaction phase I have the potential to oxidize the therapeutic protein, specifically methionine and tryptophan residues [133], leading to the structural protein modification and formation of oligomeric species [116]. In addition to the POE moiety, unsaturated fatty acids present a further locus of susceptibility to auto-oxidation. In the case of PS80, auto-oxidation has been shown to largely depend on the number of the unsaturated fatty acids, e.g., oleic and linoleic acid, and subunits [134]. Despite PS20 being mainly composed of lauric acid, it can contain up to 1% of oleic acid and 0.3% linoleic acid according to the Ph. Eu. Hence, similar to PS80, PS20 is also susceptible to auto-oxidation at the fatty acid subunits.

Another potential phenomenon in biotherapeutics containing PSs is the possibility of light-induced degradation. Therapeutic proteins have shown susceptibility to photodegradation, as tryptophan, tyrosine, phenylalanine, and cysteine/cysteine residues undergo primary photo-oxidation. This might result in the modification of the protein structure, and subsequently has an impact on the bioactivity, long-term stability, and immunogenicity of the product [135]. The light spectrum to which formulations are exposed is usually in the visible or near UV (ultraviolet) wavelength regions [136,137]. While proteins do not absorb in the visible spectrum, excipients like PSs do [68,136]. Similar to the case of auto-oxidation, the photo-oxidation of the protein therapeutic formulations stabilized with PSs leads to a series of outcomes such as structural changes and protein particle formation [16,138,139,140], chemical modification [9,135,141,142,143], partial unfolding [144], and destabilization [16,145], as well as the degradation of the PSs, which in turn produces ROS (reactive oxygen species) [11] that will further oxidize the protein, threatening the formulation integrity [68,70,71,145]. Important to remark on is the effect of protein degradation products, as is the case for tryptophan degradants L-kynurenine, hydroxyl tryptophan, and N’-formylkynurenine. Owing to unsaturations in their structures, these can absorb light and act as photosensitizers [135,136].

The use of PSs as the principal surfactant for parenteral formulations was initially granted, given their ability to ensure biotherapeutic stability as well as their low toxicity in human subjects. Nonetheless, over the last decades, the idealness of these surfactants for the stabilization of protein therapeutics has been refuted by the clinical case reports and published articles having raised concerns about the potential consequences of PS degradation in parental formulations [146,147,148,149,150,151]. The biggest concern within this context is the PS degradation-mediated protein particle formation, which not only risks the loss of therapeutic activity of the formulation, but might lead, as supported by different case reports, to severe immunogenic [146,147], and in certain cases anaphylaxis reactions [148,149,150]. In addition to the particle-induced hypersensitivity reactions, PS-mediated immunogenicity might be also triggered through the exaggerated response of the body to the POE moiety of the surfactant [152]. Single observations reported PS-mediated hypersensitivity and anaphylactic reactions in case of non-protein therapeutics, in which PSs have been used as excipients for other purposes, e.g., solubility enhancement [150,151]. Similar cases have also been reported in cases of protein therapeutics. Examples include PS-mediated anaphylactoid reactions to omalizumab [153,154], urticaria following the treatment with adalimumab and ustekinumab [155], anaphylactic response after long-term treatment with mepolizumab [156], hypersensitivity to human papillomavirus vaccine [157], and darbepoietin and erythropoietin [158].

Considering the limitations debated above, the search for alternative excipients that offer the same benefits as PSs while being devoid of the drawbacks associated therewith is highly relevant. To address the lack of excipient development, the center of drug evaluation and research (CDER) has launched the novel excipient review pilot program, which allows excipient manufacturers to propose novel excipients to the FDA prior to their use in drug formulations [159]. In the next section, we will review a number of excipients that could constitute potential candidates to serve as alternative stabilizers in injectable protein biotherapeutic formulations. Some of these still pose parts of the problems associated with PS degradation, whilst avoiding the rest. Several have already been investigated within the context of stabilizing parenteral protein therapeutics, whereas the investigation of others within this frame inspires further research.

## 3. Potential Polysorbate Alternatives for Protein Stabilization in Injectable Formulations

### 3.1. Surfactants

As surfactants are the most common class of excipients with established protein stabilization abilities, it comes as no surprise that the first group of PS alternatives are proposed from this class. Here, we classified PS alternative surfactants into two main categories. The first includes the surfactants that, like PSs, comprise an ester bond in their structure. This means that, similar to the case of PSs, these are associated with the risk of enzyme-mediated cleavage of the ester bond (Figure 1b). Unlike PS surfactants, however, some of these might be less prone to auto- and photo-oxidation and are hence expected to offer advantages in this regard (see the upcoming sections for more information) [160]. The second group involves PS alternatives, which, given the lack of ester bond in their structure, are resistant to enzymatic hydrolysis and in cases, potentially, auto- and photo-oxidation. In this section, we will debate each group of these surfactants separately and discuss selected examples.

#### 3.1.1. Surfactants Comprising Ester Bonds

The main surfactants in this category include the sucrose fatty acid esters and sugar monoesters, polyethylene glycol (PEG) stearates, and PEG fatty esters, (Figure 1a). Similar to PS surfactants, these are associated with the main advantage of biodegradability by virtue of the cleavable ester bond in their structure, and have thus been approved by the FDA for use in the food, cosmetic, and pharmaceutical industries (see FDA Inactive Ingredient Database [161]). Yet again, the ester bond renders these prone to enzyme-mediated hydrolysis when used in the formulation of protein therapeutics.

- Sucrose fatty acid esters and sugar monoesters

Sucrose fatty acid esters or sucrose esters (SEs) are non-ionic surfactants with a sucrose backbone as the hydrophilic group, and a maximum of eight fatty acids per molecule as the hydrophobic moiety (Figure 1a). The fatty acids are most commonly lauric, myristic, palmitic, stearic, erucic, and oleic acid [162]. SEs are FDA-approved excipients used mostly in the food and cosmetic sectors and, to a lesser extent, in the pharmaceutical sector [163,164]. According to the FDA Inactive Ingredients Database [161], two members of the SE class, namely sucrose stearate and sucrose palmitate, are approved for use in oral and topical formulations of small molecules [164] for drug solubility enhancement [165,166], controlled drug release [167], and drug absorption and penetration enhancement [168,169,170]. SEs are also used as emulsifiers for the formulation of microemulsions for transdermal drug delivery or in food and cosmetic application [171,172], and for the preparation of different micro- and nanoparticulate systems [173,174,175]. The most important SEs used within this context include sucrose stearate, sucrose palmitate, sucrose erucate, sucrose laurate, sucrose myristate, and sucrose oleate [176]. These are biodegradable and biocompatible surfactants, as they comprise a cleavable ester bond in their chemical structure. Following oral intake, most of these hydrolyze into FFAs and saccharide constituents, rendering them gastrointestinal digestible [177,178]. On top of that, SEs are odorless, flavorless, non-toxic, and non-irritant to the skin. For such reasons, they have been granted the GRAS (generally recognized as safe) status by the FDA [178].

As commonly used surfactants in the food industry, some information regarding the biocompatibility and safety of the SEs is already in hand. In 1992, the Scientific Committee for Food stated an acceptable daily intake (ADI) of 0–20 mg/kg body weight/day for SEs of fatty acids and sucro-glycerides derived from palm oil, lard, and tallow fatty acids. In 2004, the European Food Safety Authority (EFSA) re-examined the safety of these food additives based on new research and granted a group ADI of 40 mg/kg body weight/day for SEs of all fatty acids [176]. Additionally, the absorption, distribution, and metabolism of the SEs has been extensively investigated. Following oral intake, higher fatty acid esters of sucrose, such as octa- and hepta-esters (e.g., Olestra [179]), are excreted unmetabolized, while lower esters are partly hydrolyzed and absorbed as sucrose and individual fatty acids [180,181,182]. However, one study reported that intravenous administration of sucrose monopalmitate in a relatively high dose of 0.5 g/kg could lead to hemolytic reactions in rats [183,184]. Interestingly, no hemolysis has been reported in case of the intravenous injection of palm oil SEs and lard and tallow SEs in rats and mice, respectively [184]. These findings point out the safety of SEs, at least in animal models. Nonetheless, more profound toxicological studies are required to ensure the full safety of these surfactants following intravenous application in human subjects. At the moment, only a few articles have described the use of SEs in pharmaceutical products tested on humans, focusing mostly on transdermal delivery systems, where SEs have been utilized as emulsifiers, or else for the control of drug release and the enhancement of cutaneous absorption [185,186,187]. The only case where SEs have been used for the formulation of parenteral injectables has been the exploitation of sucrose laurate as a POE-free Cremophor^®^ EL substitute in parenteral formulations for the enhancement of the solubility of the poorly water-soluble drugs, such as cyclosporin A, docetaxel, paclitaxel, and etoposide [188,189].

Accordingly, the potential of SE surfactants for the stabilization of injectable protein biotherapeutic formulations, particularly at a mechanistic level and with regard to the minimum required concentrations as well as the long-term stability of the surfactant under different conditions, has to be investigated. Within the context of the latter, given the presence of the ester bond, SEs will not be devoid of the same drawback that complicates the use of PS surfactants in protein formulations. The chemical and enzymatic hydrolysis of the SE surfactants can raise the same issues, e.g., precipitation of the FFAs, creation of the visible or sub-visible particles, and endangering protein stability, leading to potential immunogenic reactions. However, the rate of surfactant degradation, the size of the potential FFA particles, and the minimum required concentration of the SEs for protein stabilization are factors that need to be investigated to enable a decent comparison of the SEs with the PSs. On the other hand, SEs offer obvious advantages when compared to their conventionally used counterparts. For example, the chemical structure of SEs renders them less susceptible to oxidative degradation when compared to the PSs, which can significantly improve the quality of the final product over long-term storage. Additionally, the lack of POE moiety decreases the risk of immunogenic, hypersensitivity and anaphylactic reactions. In all, while SEs seem to have potential to serve as effective PS substitutes, comparative studies are required to state conclusively as to whether SEs could perform equal or superior to PSs when used for the stabilization of protein biotherapeutics.

Similar to SEs, monoesters of sugars other than sucrose can serve as potential candidates for protein stabilization. These have been introduced since a few decades as non-toxic and biodegradable emulsifiers for use in the food industry [190]. Garofalakis et al. (2000) investigated various sugar monoesters of xylose, galactose, and lactose esterified with different hydrophobic chain lengths. All sugar monoesters could reduce the surface tension of the water in a manner comparable with commercial SEs (C8-C16 alkyl chain). The authors further observed a decreased critical aggregation concentration of the surfactants with increasing carbon chain length [191]. This early study provides a view of the unexploited potential of some sugar monoesters, from which pharmaceutical formulations could benefit. These will be, however, potentially associated with similar advantages and drawbacks detailed in the case of SEs.

- Polyethylene glycol (PEG) stearates and PEG fatty esters

Polyethylene glycol (PEG) stearates, also referred as macrogol stearates, polyoxylstearates, POE stearates, or ethoxylated stearates, are non-ionic surfactants that contain a POE (also known as PEG) segment that endows their hydrophilic character and a saturated fatty acid as the hydrophobic moiety (mainly octadecanoic acid) linked with an ester bond (Figure 1a) [192]. The average length or the molecular weight of the polymer chain is often indicated in the name of the compound, e.g., PEG 8 stearate, and the ethylene oxide monomer range can be between 2 to 150 units [192,193]. The longer the polymer chain in the compound, the more water-soluble it is [192].

According to the FDA Inactive Ingredients Database [161], four different PEG stearates are approved for use in pharmaceutical products. Within this context, PEG 40 stearate is used in formulations for ophthalmic, oral, and topical application. The use of PEG 8 and PEG 100 stearates has been approved in topical and oral formulations, whereas PEG 2 stearate is merely applicable in topical formulations. PEG stearates are common excipients in cosmetic products, mainly due to their viscosity and solubility enhancement properties, along with their low toxicity, humectant, and emulsifying properties [194].

Structurally speaking, PEG stearates, similar to PS surfactants, are prone to hydrolysis of the ester bond present in their chemical structure, as well as the oxidation of the POE moiety. However, little is known about the rate of degradation, and the potential influence thereof upon the quality of the final product. Furthermore, no information regarding the efficiency of the surfactant in stabilizing different proteins and the required concentration range is in hand. Adjunct to the factors related to the stability of these surfactants, the potential interactions of the PEG stearates with the components of the primary packaging and the subsequent influence upon the final product quality is yet to be investigated.

However, as these are associated with relatively safe toxicological profiles, further investigations regarding their potential application for protein stabilization are meaningful. PEG stearates have been non-lethal in animal tests at a concentration of 10 g/kg body weight, leading to minimal ocular irritation in rabbits and resulting in merely low-level skin irritation in rabbits when tested at 100% concentration, and induced no irritation in humans [194]. While low molecular weight PEG-2 stearate and PEG-9 stearate can influence the skin barrier function at concentrations of 5% *w*/*v*, higher molecular weight PEG-40 stearate does not exert the same effect [195]. Feeding animal models with PEG-8, −40, and −100 stearates accounted for no impact upon the mortality rate, growth, and histopathological and hematological abnormalities [196]. Similarly, PEG fatty acid esters have been shown to induce no genotoxic effects [193,196,197], while carcinogenic and chromosomal aberration data are yet to be provided.

Another FDA-approved surfactant belonging to the PEG fatty esters is polyoxyl 15 hydroxy stearate, commercially labelled as Kolliphor^®^ HS 15 and Solutol^®^ HS 15. This surfactant has been approved by the FDA for the improvement of solubility of low water-soluble drugs in parenteral and oral formulations [198]. Kolliphor^®^ HS 15 is a non-ionic surfactant synthesized using 1 mol 12-hydroxystearic acid and 15 mol ethylene oxide (Figure 1a) [199]. Its chemical structure is based on 12-hydroxystearic acid (lipophilic moiety) polyethoxylated at both the carboxyl and the hydroxyl groups with PEG (hydrophilic moiety). As a mixture, Kolliphor^®^ HS 15 also contains free PEG as a side product of the epoxidation reaction [199]. It possesses a hydrophilic–lipophilic balance (HLB) value of about 16 and a critical micelle concentration (CMC) ranging from 0.06 and 0.1 nM when dissolved in water at 21 °C [198,200]. Due to these properties, Kolliphor^®^ HS 15 has efficient solubilization properties, having been thus used mainly for the solubilization of poorly soluble vitamins and hydrophobic drugs, such as clotrimazole, carbamazepine, ketoconazole, danazol, piroxicam, fenofibrate, and cinnarizine [198,201,202,203]. Kolliphor^®^ HS 15 can also be used to protect macromolecules and some protein formulations [204]. As an FDA-approved non-ionic surfactant, Kolliphor^®^ HS 15 has been used in oral, parenteral, and ophthalmic formulations for several years [205]. It has also been investigated for the development of various types of injectable nanoparticle formulations, including but not limited to self-emulsifying drug delivery systems, solid self-emulsifying drug delivery systems, lipid nanocapsules, solid lipid nanoparticles, and nanostructured lipid carriers, as well as other liquid and solid dispersion formulations [198]. Within the context of the marketed injectable drugs, it has been mostly used as a component of non-biotherapeutic formulations. These include injectable formulations of ibuprofen eugenol ester [206], propofol (2,6-diisopropylphenol) [207], and a mixture of α-tocopherol, ascorbic acid, and β-carotene [208].

The toxicological profile of Kolliphor^®^ HS 15 has been hitherto investigated, having shown a superior safety and toxicity profile in comparison with PS80 [209]. In animal models, Kolliphor^®^ HS 15 shows no acute toxicity in rats (intravenous LD_50_ > 1 g/kg body weight, oral LD_50_ > 20 g/kg body weight), mice (oral LD_50_ > 20 g/kg body weight), dogs (intravenous LD_50_ > 3 g/kg body weight), and rabbits (intravenous LD_50_ > 1 g/kg body weight) [192,210]. Parenteral administration of 100 mg/kg body weight of Kolliphor^®^ HS 15 has been shown to induce pseudo-allergic reactions associated with histamine release, though no histopathological changes or signs of specific toxicity have been observed [210]. These allergic reactions, however, have not been limited to Kolliphor^®^ HS 15, and have been observed in the case of Kolliphor^®^ EL, another PEG fatty acid, and PS80. Overall, the results of these investigations conclude the safety and low toxicity of Kolliphor^®^ HS 15 as a non-ionic surfactant in comparison with other commercial non-ionic PEG-based surfactants [199].

Few studies have shown the ability of Kolliphor^®^ HS 15 to stabilize proteins, e.g., amylase and bovine serum albumin (BSA) [204]. However, as the ester bond available in the structure of Kolliphor^®^ HS 15 is chemically and enzymatically cleavable, the surfactant is expected to be, at least to some extent, associated with similar drawbacks as PS surfactants. Furthermore, the presence of the POE moiety renders the molecule susceptible to oxidative degradation [211,212]. Here again, the rate of surfactant degradation along with the impact of degradation products on the protein stability as well as quality of the final product has to be investigated in detail, in order to provide a comparative overview of the surfactant’s efficiency in comparison to PSs for the stabilization of protein therapeutics. As in the case of any other excipients used for parenteral biotherapeutic formulations, the analytical characterization of the surfactant to detect impurities, as well as the plausible side products from the auto-degradation process, is important for quality control. Several techniques have been hitherto developed to quantitatively examine Kolliphor^®^ HS 15 composition. Examples include the use of ultraperformance liquid chromatography coupled with a nano quantity analyte detector to quantify the lipophilic moieties as well as hydrophilic free PEGs in the formulation [213], or else application of gel permeation chromatography to quantify Kolliphor^®^ HS 15 structural components [214]. Another most outstanding method is the use of liquid chromatography tandem mass spectrometry with an ion suppression effect, through which the identification of twelve oligomers by electrospray ionization has been possible. Based on these analyses, the lipophilic and hydrophilic components in Kolliphor^®^ HS 15 have been quantified to be around 63.3% and 36.7%, respectively [215,216]. Notwithstanding, except for the FFAs, little has been practically uncovered about the degradation products of this surfactant and how these will affect protein stability. Moreover, the potential interactions of the surfactant with the primary packaging have not been fully investigated either. Hence, a thorough investigation of the applicability of Kolliphor^®^ HS 15 for the stabilization of protein biopharmaceutics, the stability of the surfactant and the effect of potential degradation products on the quality of the formulation, and the potential interactions of the surfactant with the primary packaging inspires further research.

Similar to polyoxyl 15 hydroxy stearate, polyoxyl 35 castor oil (also known as Cremophor^®^ EL and Kolliphor^®^ EL) is a non-ionic surfactant belonging to the PEG fatty ester family (Figure 1a). It is made of reacting castor oil and ethylene oxide in a molar ratio of 1:35, and has an HLB value in the range of 12 to 14 [217,218]. The surfactant has been approved by the FDA for use in pharmaceutical formulations through intramuscular, intravenous, intravesical, subcutaneous, ophthalmic, sublingual, and topical administration routes, and it has been mainly used for the parenteral delivery of small molecular drugs [161]. In one study, the ability of Kolliphor^®^ EL to stabilize amylase over two months in the presence of hydrogen peroxide has been compared to that of Kolliphor^®^ HS 15 and PS80. Fluorescence measurements revealed the ability of Kolliphor^®^ EL in the preservation of amylase and the reduction of the oxidative degradation to be superior to that of PS80. In addition, similar to PS80 and Kolliphor^®^ HS 15, Kolliphor^®^ EL could efficiently protect the BSA exposed to mechanical stress for a period of 7 days [204].

Although the FDA and EMA have confirmed the low oral toxicity and non-mutagenicity of Kolliphor^®^ EL, anaphylactic reactions associated with parenteral formulations, in which the surfactant has been used as a delivery vehicle or an excipient, have been reported [219,220,221,222,223,224,225]. This is not a side effect specific to Kolliphor^®^ EL, but a drawback shared by all PEG (POE) containing excipients. Similar to the PS structures, the PEG moiety can induce mild to strong immunogenic and hypersensitivity reactions. Between January 1977 and April 2016, 37 case reports of immediate-type hypersensitivity to PEG emerged, of which 28 (76%) described hypersensitivity reactions that met anaphylaxis criteria [226]. This shared drawback of all PEG containing excipients is not taken seriously, as certain proteins or their aggregates can also trigger immunogenic reactions of their own, or else induce the secretion of various immunoglobulins [227,228,229]. Combined, these might lead to immunogenic reactions ranging from milder site-of-injection swelling and pain to hypersensitivity reactions and anaphylactic reactions in extreme cases [152,230]. Hence, more detailed clinical studies should be dedicated to uncovering the full extent and the reasons behind the immunological concerns raised in the case of PEG-based surfactants to thoroughly address the issue of the suitability of the surfactant for the stabilization of injectable protein formulations.

#### 3.1.2. Non-Ester Surfactants

**Figure 2:**
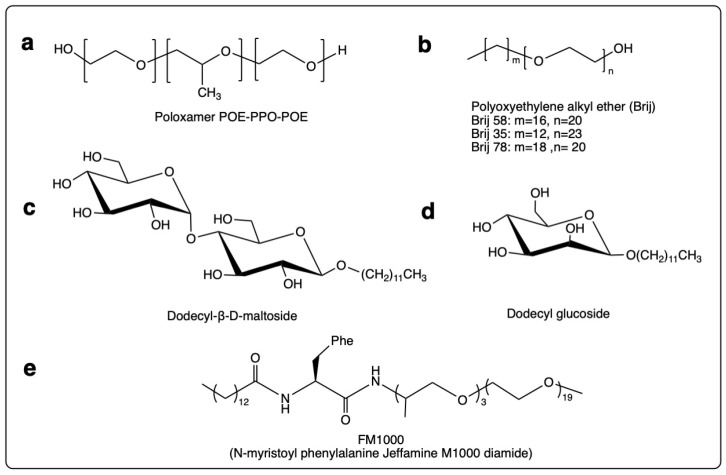
Chemical structures of potential surfactant PS alternatives devoid of an ester bond: (**a**) Poloxamer, (**b**) Polyoxyethylene alkyl ether (Brij), (**c**) Dodecyl-β-D-maltoside, (**d**) Dodecyl glucoside, and (**e**) N-myristoyl phenylalanine Jeffamine M1000 diamide (FM1000).

The benefit of the non-ester surfactants mainly lies in their lack of sensitivity to enzymes currently detected in protein formulations. The most important surfactant class of these include block polyethylene-propylene glycol and polyoxyethylene fatty ethers, as well as non-ester sugar-based surfactants such as dodecyl glucoside and dodecyl maltoside, and N-alkyl amino acid polyether amides such as N-myristoyl phenylalanine Jeffamine M1000 diamide (FM1000) (Figure 2).

- Block polyethylene-propylene glycol

Triblock copolymers of polyethylene oxide-polypropylene oxide-polyethylene oxide (POE-PPO-POE), commercially available as Poloxamers^®^ and Pluronics^®^, possess surfactant properties resulting from the hydrophobic PPO block in the middle and the hydrophilic POE blocks on the sides (Figure 2) [231]. Different Poloxamers^®^ vary mainly in the molecular mass of the PPO core and the percentage of POE content [232]. The name of the copolymers commonly starts with the letter “P” (for Poloxamer^®^) followed by three digits, the first two of which, when multiplied by 100, give the approximate molecular mass of the PPO core. Multiplication of the last digit by 10 provides information of the percentage POE content [160,233]. The two most well-known members of this family are Poloxamer^®^ 188 (P188; PPO molecular mass of 5400 g/mol and 80% POE content) and Poloxamer^®^ 407 (P407; PPO molecular mass of 4000 g/mol and 70% POE content). According to the FDA Inactive Ingredient Database, P188 is approved for use in a variety of formulations as an emulsifying, solubilizing, defoaming, and dispersing agent. In fact, P188 is one of the well-considered PS alternative surfactants, as it is already incorporated in commercial biotherapeutic formulations such as Gazyva^®^ (0.2 mg/mL) [234,235], Orencia^®^ (8 mg/mL) [236], Norditropin^®^ (3 mg/mL) [237], and Hemlibra^®^ (0.5 mg/mL) [234].

Since Poloxamers^®^, as efficient stabilizers of injectable protein biotherapeutics, have hitherto successfully found their way to the market, it is worth first detailing the stability and potential degradation of these surfactants [160]. P188 has been shown to degrade in the solid state at high temperatures due to auto-oxidation and chain cleavage, resulting in aldehydes, organic acids, and smaller molecular weight polymers [238]. The solid-state degradation of the P188, trackable based on the pH shifts, aldehyde formation, and changes in molar mass of the polymer has been shown to initiate at around 40 °C [238]. P407, on the other hand, has been shown to be stable, and the thermal degradation only initiated after 21 days at 80 °C, starting from the PPO center block, resulting in chain scissions in both PPO and POE polymer blocks [238,239]. In this study, P188 comprised less antioxidant compared to the P407, which the authors mentioned as a potential reason behind the higher sensitivity of the former to solid-state degradation. Based on the proposed mechanism, the solid-state degradation of P407 is associated with the generation of free radicals [239]. It should be, however, noted that these degradation reactions are triggered at higher temperatures, which are at any rate deteriorating to the stability of the protein and are hence avoided during the production, storage, and administration phases of protein biotherapeutics.

Although the chemical degradation of P188 and P407 in the solid state has been examined and published in greater depth, their chemical stability in solution has not been thoroughly investigated [240]. In one of the few studies in the subject, Wang et al. (2019) examined the degradation of P188 in solution as a function of the buffer type, pH, temperature, trace metals, and peroxides [240]. The results obtained using direct injection gas chromatography-mass spectroscopy were indicative of significant degradation of the polymer in 10 mM histidine buffer. Within this context, the thermal oxidation of the PPO block initiates the oxidation of P188 triggered by the secondary radicals in the polymer chain, which leads to secondary hydrogen peroxide. As a result, the oxidation of histidine could be catalyzed by both secondary free radicals and secondary hydrogen peroxide produced during the P188 oxidation process [240]. The authors concluded that, relative to PS surfactants, P188 has a lower stability liability in liquid formulations [240].

Recently, the stabilizing properties of PSs and P188 in mAb formulations under different stress and storage conditions have been compared [241]. These studies have indicated that both PSs and P188 could effectively protect mAbs from interfacial stress in liquid vial formulations. However, unlike formulations containing PSs, clear protein–polydimethylsiloxane (PDMS) particles were observed in vials of protein formulations stabilized using P188 after long-term storage at 2–8 °C. Moreover, the use of P188 as surfactant in vials with a bromobutyl teflonized and siliconized stopper led to the formation of molecule-specific visible and sub-visible protein–PDMS particles in liquid mAb formulations, when stored for an extended period of time [241]. The protein–PDMS particles found in this study have been attributed to the siliconized stopper used in the primary packaging, resulting from a shift of silicone oil traces from the non-product contacting side to the product contacting side during the bulk storage and processing of the stoppers [241]. Hence, while P188 seems to be a useful alternative to PSs for stabilizing liquid mAb formulations, a change of chemical composition of the PDMS stoppers is necessary to avoid the formation of protein–PDMS particles during storage, even at low temperatures. Even in cases where non-PDMS stoppers have been used, Fourier-transform infrared microscopy has revealed the formation of fluoropolymer particles in the solution. On the other hand, a related study examining the impact of excipients on the functionality of prefilled syringes discovered that when compared to P407 and P188-containing formulations, those containing PS80 showed a significant increase in glide force. While this was mostly observed at higher temperatures (40 °C), which indicates a low risk of syringe failure during storage at room temperature, an increase in protein concentration seems to accelerate this process and result in an increase of glide force at 25 °C. In comparison, the performance of P188 in terms of maintaining syringe functionality and stability, particularly in the case of HCLF, was shown to be superior [242].

To sum up, it seems that the use of Poloxamers^®^, including P188 and P407, as protein stabilizers in injectable formulations has both advantages and disadvantages depending on the physicochemical traits of the protein, formulation ingredients, and the type of packaging or delivery system in which the formulation is contained. Being FDA-approved excipients already in use for the formulation of protein injectables, these are not associated with safety and toxicological concerns, as is the case for the PS surfactants. However, unlike PSs, Poloxamers^®^ are not susceptible to HCP-mediated degradation. Nevertheless, it cannot be denied that similar to PSs, the use of Poloxamers^®^ in injectable protein formulations is still fraught with concerns regarding the instability of the surfactant and the subsequent impact on the protein integrity, the formation of visible and sub-visible particles, and the potential biological interactions. More in-depth research is yet to be conducted to thoroughly address such concerns and to optimize the protein formulations and primary packaging to fully benefit from the use of Poloxamers^®^ as PS alternatives in such formulations. Moreover, concerns regarding the potential immunological, hypersensitivity, and even anaphylactic reactions originating from the PEG (POE) moiety of these polymers must be addressed.

- Polyoxyethylene fatty ethers

POE ethers (commercially available as Brij^®^) comprise the POE (PEG) chain as a hydrophilic moiety, and the ethoxylated fatty alcohol constituting the lipophilic portion of the surfactant (Figure 2). By using different types of fatty alcohols (lauryl alcohol, cetyl alcohol, stearyl alcohol, and oleyl alcohol), different glycol ethers can be synthesized. According to the FDA Inactive Ingredient Database, Brij^®^ (cetomacrogol) is a polyether-based surfactant approved as an inactive ingredient solely for topical formulations. This is reflected in the review publications compiled by Jiao (2008) [243] and Sahoo et al. (2014) [244] focusing on the use of POE alkyl ethers (Brij^®^ 35, 78, 98) in pharmaceutical formulations, which mainly comprises a discussion of their role in the development of topical and ocular formulations. In addition, the stabilizing effect of POE ethers in oral protein formulations has also been investigated by Mesiha et al. (1981). The authors demonstrated that the incorporation of Brij^®^ 58 as a surfactant enhanced the stability of orally administered insulin in male white rabbits [245].

Since POE ethers have shown to provide good stability in topical formulations, their stabilizing effect in protein-based liquid formulations has been compared to the PS surfactants. Bam et al. (1995) investigated how well Brij^®^ 92 could stabilize the rhGH (recombinant human growth hormone) as a model protein in comparison with PS80 as a reference surfactant [246]. The surfactant activity was measured through the partitioning behavior of a spin-label with electron paramagnetic resonance to determine the binding stoichiometry [247]. The obtained results indicated that Brij^®^ 92 tends to behave in a similar way as PS80 and could effectively stabilize the rhGH protein [246]. Similarly, through light-scattering experiments, Krause et al. (2002) confirmed that when used in a concentration of 0.001 mg/mL, Brij^®^ 58 could effectively inhibit the protein particle formation of chemically denatured proteins (glucosidase, citrate synthase, and rhodanese) [248].

Even though the FDA approval of POE ethers as surfactants for parenteral formulations is yet to be obtained, the last decade has witnessed a more detailed investigation of the role of Brij^®^ as a surfactant in injectable biotherapeutic formulations [249]. For instance, Agarkhed et al. (2018) explored the effect of PS, Brij^®^ 35, and several other POE-based surfactants on the mechanical, thermal, and photostability of a mAb injectable formulation [250]. According to the findings of the mechanical and isothermal stability, as well as the sub-visible particle counting, while all tested non-ionic surfactants demonstrated a similar protein protecting effect, their performance was inferior to that of PS80. On the other hand, formulations comprising non-PS surfactants, including Brij^®^ 35, could better protect the mAb against photo-oxidation, as was evident from the decrease in methionine and tryptophan oxidation [250]. In one of the latest studies on the subject, Yue et al. (2020) proposed Brij^®^ 58 as a good alternative surfactant to PS20 and PS80 for the stabilization of protein injectable formulations [249]. Through visual inspection and dynamic light scattering, the authors confirmed superior mAb protection ability of Brij^®^ 58 under forced degradation conditions. Furthermore, ultra-performance liquid chromatography- charged aerosol detector investigations revealed better inherent stability of the surfactant in protein formulations when compared to PS20 and PS80. After 19 days of shaking and incubation at 40 °C for 1 month, no significant reduction of the Brij^®^ 58 concentration was observed, while the concentrations of PS20/PS80 underwent an approximate decrease of 30%. On top of that, the required concentration of Brij^®^ 58 as a surfactant was comparably low (0.075 mg/mL versus 0.2 mg/mL in case of PS20/ PS80). In terms of toxicological investigations, the estimated LD_50_ in mice was equal to 343.4 mg/kg body weight, which was lower than that reported for PS80 (5000 mg/kg body weight). The authors argued that this would still leave a large safety window for Brij^®^ 58 in protein formulations, as the required concentration of the surfactant is quite low. Indeed, no side effects were observed in acute toxicological studies when administrated in a concentration of 20 mg/mL in mice [249]. While these findings highlighted the potential of Brij^®^ 58 to be used as an efficient PS alternative in the formulation of injectable protein biotherapeutics, the authors also emphasized that the safety of the surfactant in human subjects when incorporated in parenteral formulations is yet to be investigated [249].

In terms of stability, little has been reported about POE ethers in general. One might argue that as non-ester surfactants, POE ethers might be superior to PSs in terms of susceptibility to hydrolysis. On the other hand, since Brij^®^ surfactants contain POE and alkyl chains, the likelihood that they might undergo a similar oxidative degradation to PSs cannot be completely ruled out. The literature holds only a few studies on Brij^®^ degradation in pharmaceutical formulations [78,251]. The most relevant publications within this context report a metal-mediated oxidation in case of Brij^®^ 30 [78] and a photocatalytic degradation of Brij^®^ 35 in the presence of inorganic minerals [251]. The ether moieties available in the structure of Brij^®^ molecules are susceptible to auto-oxidation in contact with air through the formation of hydroperoxides. The oxidation process begins with the formation of a free radical at the α-carbon atom of the ether moiety. According to Currie et al. (2004), the presence of silver ions leads to the removal of hydrogen, which usually is caused by oxygen in non-metal-mediated oxidation processes. Particularly in this metal-induced oxidation of Brij^®^ surfactants, the susceptibility of long alkyl chains to oxidation is greater than that of short alkyl chains. This has been attributed to the high hydrogen fed capacity to the oxidative process by virtue of the several repetitive methylene units in the saturated carbon chain [78]. The photocatalytic oxidation of POE moiety, on the other hand, is promoted by UV light incidence and the presence of a photocatalyst. The increase of oxygen concentration influences the rate of oxidation, but only up to a defined concentration where the system reaches a plateau [251]. With all this said, more profound research is needed to address the stability of the Brij^®^ surfactants in protein biological formulations and the influence of the degradation products on the stability of the protein cargo. Based on the available data at least, Brij^®^ surfactants seem to have superior stability to PS surfactants, and they seem to be valid candidates for further investigations within the context of stabilizing parenteral biotherapeutic formulations. However, concerns regarding the potential immunological, hypersensitivity and even anaphylactic reactions originating from the POE moiety of these surfactants must be addressed.

- Non-ester sugar-based surfactants

Non-ester sugar-based surfactants such as dodecyl glucoside and dodecyl maltoside (Figure 2) have been shown to reduce the surface tension of water in a matter comparable with SEs [191]. EPA (Environmental Protection Agency) has reported dodecyl maltoside to be a non-mutagenic, non-toxic, and non-irritating substance [252]. Dodecyl maltoside was/is further patented for use in nasal, parenteral, and hydrogel formulations [253]. In the case of different alkyl-β-glycosides, degradation following oral administration and the metabolism of the lipid and carbohydrate moieties through the relevant metabolic pathways have been shown [190]. Being safe for human consumption, n-dodecyl-β-D-maltoside (DMM) has been exploited as a non-ionic alkyl saccharide surfactant for the stabilization of a therapeutically active peptide (D-alanine Peptide T-amide (DAPTA)) during lyophilization and the subsequent reconstitution of the freeze-dried cake [253]. The surfactant could successfully extend the product shelf-life independent of the lyophilization-thawing process and could further increase the stability and reduce the aggregation and immunogenicity of the therapeutic peptide [253]. In a later study, DMM was used as a surfactant to stabilize interferon-β in intranasal and subcutaneous formulations for the treatment of multiple sclerosis [254]. Within this context, DMM could successfully prevent protein particle formation in solution and reduce the immunogenicity of the final formulation [254]. Despite these promising results, the use of this surfactant in the formulation of parenteral protein therapeutics in the market remains underexploited.

- N-alkyl amino acid polyether amides

N-alkyl amino acid polyether amides are a series of chemically defined surfactants with an amino acid as an alkyl tail linked to the polyether hydrophile through amide bonds [255]. These have been developed to reconcile the protein stabilization properties of the amino acids (detailed under amino acid-based stabilizers) with that of the non-ester surfactant families. Due to the presence of the amide bond, rather than an ester bond, N-alkyl amino acid polyether amides are resistant to the described HCP-mediated hydrolysis, while they might be still associated with the problem of oxidation as in the case of PSs. N-alkyl amino acid polyether amides also offer the advantage of low batch to batch variability due to their chemically defined structure [44]. One paramount example within this context is N-myristoyl phenylalanine Jeffamine M1000 diamide (FM1000; Figure 2), which, when used in a concentration of 1 mg/mL, could significantly slow down the thermal-induced aggregation of 10 mg/mL abatacept. Dynamic light scattering (DLS) measurements and size exclusion chromatograms demonstrated the reduction of thermal-induced protein particle formation. Interestingly, compared to PS20, PS80, and P188, FM1000 resulted in 3-fold more reduction of protein particle formation [255]. Following these interesting findings, the efficiency of FM1000 for the stabilization of liquid biotherapeutics under an induced mechanical stress was also investigated [256]. To this end, the efficiency of FM1000 to inhibit protein particle formation in an immunoglobulin G (IgG) formulation at a concentration of 20 mg/mL following exposure to 24-h-long agitation was compared to that of PS20, PS80, and P188. Interestingly, the rate of protein particle formation was highest in the case of P188, followed by PS80 and then FM1000 and PS20. Compared to conventional surfactants, FM1000 could better facilitate the stabilization of air/water and air/silicon interfaces in IgG containing formulation [256]. Furthermore, the effect of FM1000 upon the agitation stress of abatacept in IV bags was explored. The protein stability was highest in the case of FM1000, followed by P188, PS80, and PS20. The surface tension measurements revealed that under the same conditions, the CMC of FM1000 was 10 times lower than that of PS80 (0.55 versus 5.5 PPM) [257]. While the use of these amino acid-based surfactants for protein stabilization has been successfully patented [257], their industrial translation necessitates more in-depth investigations in terms of potential pharmacological and toxicological side effects. It should also be highlighted, that the current available stability studies were published by the manufacturer of FM1000; thus, it is unclear whether “negative” examples related to protein stabilization of FM1000 would have been disclosed.

### 3.2. Carbohydrates and Their Derivatives

**Figure 3:**
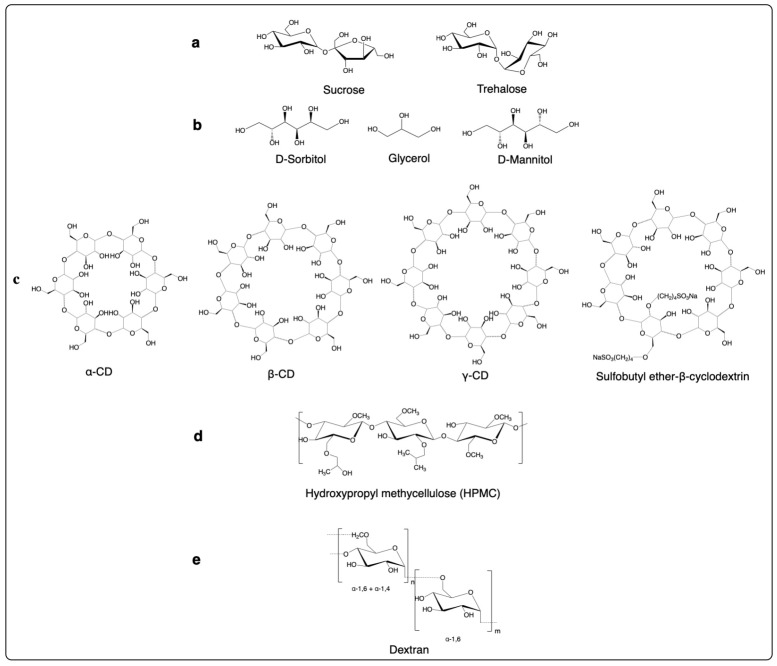
Chemical structures of potential carbohydrate PS alternatives: (**a**) Disaccharides, (**b**) Sugar alcohols, (**c**) Cyclodextrins (CD), (**d**) Hydroxypropyl methylcellulose, and (**e**) Dextran.

This category of potential alternative excipients includes disaccharides and their chemical derivatives, sugar alcohols, cyclodextrins, and semisynthetic polysaccharides. These are natural or in cases semi-synthetic compounds associated with an acceptable safety profile and have been hitherto exploited for protein stabilization, mainly during lyophilization. The chemical structures of these compounds are presented in Figure 3.

- Disaccharides

Disaccharides such as sucrose and trehalose are well-known for their ability for protein cryopreservation and protein stabilization in the lyophilized state [258]. What might be less widely known is their ability to stabilize proteins in solution. As osmolytes, sucrose and trehalose exert their protein stabilizing effect indirectly by altering the solvent properties and the consequent protein–solvent interactions [259]. By surrounding the protein molecules, they also separate them from one another, thereby reducing the protein–protein interactions [259]. Sucrose (Figure 3a) has been shown to inhibit the protein particle formation of recombinant interleukin-1 receptor antagonist (IL-1ra), which tends to form a soluble dimer during storage at 30 °C under minimal stressful conditions [260]. It is proposed that proteins with an increased surface area tend to be thermodynamically less stable in the presence of sucrose compared to those with a more compact structure. This could lead to a shift of equilibrium from the aggregated to the unaggregated species over time [260].

Trehalose (Figure 3a), according to the literature, acts superior to sucrose in terms of protein stabilization, mainly through its specific solvent structure and dynamics [261]. Sun et al. (1998) demonstrated the superior ability of trehalose/glucose over sucrose/glucose mixture to preserve the stability and the biological activity of a dehydrated dehydrogenase enzyme, after long-term storage at 44 °C (ca. 80 days vs. 5 days), 60 °C (>70 h vs. ca. 50 h), and 75 °C (ca. 30 h vs. <10 h) [262]. It has also provided protection and stabilization of the enzymes like restriction during the vacuum-drying process [263]. Accordingly, trehalose is a well-established excipient for the cryo- and lyoprotection of different proteins.

Research has highlighted the effective concentration of trehalose to maintain the protein stability during parenteral biotherapeutics (bevacizumab, ranibizumab, trastuzumab, recombinant antihemophilic factor) to oscillate between 10–100 mg/mL, but it is usually used in combination with PS surfactants, as it cannot provide adequate protein stability in the absence of the latter [264]. Since the pharmaceutical industry currently endeavors towards finding a substitution for polyethoxylated surfactants in biotherapeutic formulations, unusual approaches have been taken to provide trehalose better protein stabilization by modifying the molecule’s chemical structure [265,266,267,268,269,270]. One of these strategies involves the modification of trehalose with fatty acids to provide the molecule surfactant properties (similar to sucrose fatty acid esters). Schiefelbein et al. (2010) synthesized 6-O-monolauroyl- α, α-trehalose, and investigated its ability to stabilize human growth hormone (hGH) subjected to agitation stress. At a concentration of 1 mg/mL, the trehalose-based surfactant could stabilize the protein as effectively as PS20 and PS80 [265]. As a trehalose fatty acid ester, 6-O-monolauroyl-α, α-trehalose is prone to hydrolysis. Thus, a similar synthetic approach was proposed by Messina et al. (2017), who produced 4 trehalose regioisomers with an ether moiety. The compound was shown to prevent protein particle formation (>97%) against thermal and mechanical-induced stresses [266]. In a recent study, synthetic linear and 4-arm star glycopolymers (mannose, galactose, arabinose, lactose, and trehalose) were investigated in terms of maintaining the conformational stability and reducing the protein particle formation tendency of mAb1 as a model protein [267]. The stress conditions used in this study involved storage at 25 °C and 40 °C for up to 7 weeks. According to the static light scattering and differential scanning calorimetry results, the conformational and colloidal stability of the mAb1 were affected by the sugar monomer concentration as well as the polymer structure (linear or star). Unlike their low molecular weight sugar counterpart, which increased mAb 1 formulation stability even at low concentrations, glycopolymers had the opposite effect, leading to the destabilization of the Ab [267]. This was most pronounced in the case of trehalose-based glycopolymers at higher concentrations and during long-term storage both at 25 °C and 40 °C for up to 7 weeks. These results demonstrate that unlike low molecular weight sugar excipients, their corresponding glycopolymers cannot act as efficient protein stabilizers [267]. In another study, Mancini et al. (2012) synthetized a trehalose sidechain glycopolymer further conjugated to a thiolated hen egg white lysozyme. This approach led to superior stability of the lysozyme against lyophilization, and high temperatures compared to POE and trehalose excipients [268]. Nonetheless, it also required the modification of the protein, which can potentially affect the protein’s activity. In a different study, trehalose glycopolymers, along with four different monomer modifications, were synthetized, and their efficiency in terms of preserving the activity of horseradish peroxidase and glucose oxidase at higher temperatures and after several lyophilization cycles was investigated [269]. In comparison with trehalose monomers, the polymerized glycopolymer made of four trehalose monomers better preserved the protein activity and conformational stability. As an additional benefit, the glycopolymer was shown to be non-toxic for different cell lines when examined up to a concentration of 8 mg/mL [269]. In the most recent study, the authors continued their research on the same trehalose glycopolymer by chemically conjugating it to insulin. This prevented insulin from undergoing thermal and agitation-induced protein particle formation in solution. When tested in mice, the conjugate had a significantly more prolonged plasma circulation than the free insulin [270]. Furthermore, from a safety perspective, the glycopolymer has been shown to be biocompatible and non-toxic to mice when administered 1.6 mg/kg body weight [269].

The above-debated studies highlight both the benefits and limitations of disaccharides for the stabilization of protein biotherapeutics. Disaccharides offer the advantage of safety and lack of toxicity for parenteral, particularly intravenous, use. Sucrose and trehalose are FDA-approved GRAS excipients commonly used in the injectable formulations. While these are excipients of choice for the protection of the proteins against thermal and lyophilization stresses, their ability to protect the proteins in solution remains to be optimized. In many cases, the use of sucrose and trehalose alone cannot ensure adequate protein stability, as these can only minimize protein–protein interaction to a certain degree and are not as effective against sheer and interface stresses [258]. To overcome this issue, other classes of glycopolymers based on the disaccharides have also been developed. However, a further issue to consider is the excipient stability. Both sucrose and trehalose are susceptible to acid hydrolysis, though sucrose is much more prone to this phenomenon than trehalose [271]. Hydrolysis of disaccharides creates reducing sugars, which must be avoided as they can damage the proteins. Nevertheless, this is usually not an issue unless the pH is lower than 4, which is not the case for protein biotherapeutics [84]. Reducing sugars such as lactose or maltose must be avoided, as they can chemically interact to proteins through the Maillard reaction during storage [84]. Taken together, while disaccharides might not be adequate PS alternatives in their crude form [264], they can provide a base for chemical modification, or else be used in combination with other classes of stabilizers, which can hopefully pave the way for the synthesis of more suitable PS alternatives.

- Sugar alcohols

Sugar alcohols such as glycerol, sorbitol, and mannitol (Figure 3b) stabilize proteins in solution as well as during lyophilization or storage [272,273,274]. Similar to trehalose and sucrose, these mainly serve as osmolytes, protecting the integrity of the protein by altering the protein–solvent interactions [272], and by replacing the water molecules during lyophilization [273,274]. Glycerol has been shown to shift the conformation of the native protein towards a more compact state [275]. It also inhibits protein unfolding and aggregation through the stabilization of aggregation-prone intermediates. The main reason behind this phenomenon is the amphiphilic nature of glycerol, which arranges at the interface of the hydrophobic protein regions and the polar solvent [275]. To what extent glycerol can improve the protein stability is, however, dictated by the nature of the protein in question.

Mannitol, a hexahydro alcohol, is often used as a bulking agent in freeze-dried and spray-dried formulations [276,277]. In an amorphous state, mannitol also provides protein stabilization during lyophilization, as it provides intimate H-bonding with the protein at molar ratios of 360:1 or even higher. This leads to the shift of the lattice structure of the protein towards an amorphous state, which renders these more stable than their crystalline counterparts [276,277]. However, research has also demonstrated that the protein stabilization effect of mannitol occurs up to a certain concentration of the excipient. In spray-dried recombinant humanized mAb formulation, mannitol has been shown to exert a protective effect up to an excipient: protein ratio of 200:1. At higher concentrations, the crystallization of mannitol exerts a detrimental effect upon the protein stability [278]. Literature holds many other reports describing the detrimental effect of crystallized mannitol upon protein stabilization [279,280,281,282,283]. On the other hand, a recent review details varying techniques to achieve a successful lyophilized injectable formulation without the inconveniences that mannitol conveys. As a detailed discussion about the optimization of freeze-drying cycle parameters in multi-component formulations is out of the scope of the current paper, the reader is referred to a detailed review by Thakral et al. (2022) for further information [284]. The excipient to protein ratio has also been reported as essential to maintain the stability of lyophilized protein formulations during storage [285]. Today, mannitol, along with PSs, is a component of several marketed mAb formulations, including but not limited to Humira^®^, Simulect^®^, and Ilaris^®^ (Ilaris liquid formulation contains mannitol, while the lyophilized formulation contains sucrose instead) [15,286].

Sorbitol is another common sugar alcohol used in marketed mAbs parenteral formulations such as Vyepti^®^, Anthim^®^, Simponi^®^, etc [286]. It has been shown to protect the proteins in a manner similar to mannitol but is associated with the drawback of recrystallization over time, which negatively impacts the protein stability during long-term storage [287,288]. As in the case of mannitol, excipient to protein ratio seems to play an important part, with sorbitol-recrystallization-mediated protein instability being less pronounced when lower protein concentrations are used [288]. The combination of sorbitol and sucrose has shown a synergistic protein stabilizing effect [289,290].

Like disaccharides, sugar alcohols possess acceptable safety profiles for intravenously injectable formulations. Glycerol, the backbone of many lipids, is found in vegetable and animal fats and oils within regularly consumed foods. Following ingestion, glycerol is absorbed from the intestine and then follows two degradation pathways; it is transformed to glycogen and carbon dioxide or used as a precursor for the anabolism of fatty acid in the body [291]. Glycerol has been listed as GRAS, included in the FDA Inactive Ingredients Database for pharmaceutical applications, and added to the Canadian list of acceptable non-medicinal ingredients. Given its metabolism in the human body, glycerol is widely employed in parenteral, topical, oral, and ophthalmic formulations, as well as a solvent and sweetener [292]. In general, glycerol as a food additive or excipient has been proven to be non-irritant and non-toxic [291]. Low concentrations of glycerol for oral formulations present scarce side effects [293]. Nevertheless, high oral doses of glycerol can induce hyperglycemia, nausea, headache, and thirst. Intravenous or oral administration of glycerol for the reduction of cerebral edema [294] has been linked to side effects such as hemolysis, hemoglobinuria, and renal failure [295,296]. Of course, the concentration of glycerol used for such purposes is potentially much higher than the amounts necessary to stabilize protein formulations. Another side effect of high dosage use is the mucous membranes and skin irritation [297].

Mannitol and sorbitol are also GRAS, FDA-approved excipients and, as previously mentioned, part of the marketed injectable formulations. They are listed in the FDA Inactive Ingredients Database for injectable formulations, and also in the Canadian List of Acceptable Non-medicinal Ingredients [291]. The safety of these ingredients has been established based on the lack of evidence signifying their systemic toxicity, irritation, sensitization, and adverse clinical reports [291]. While mannitol is partially metabolized by the gut microbiota, it is not degraded following intravenous administration. Both mannitol and the products of its partial metabolism are cleared through renal excretion in a few hours [298,299]. Sorbitol, on the other hand, is rapidly metabolized through normal glycolytic pathways, ultimately creating carbon dioxide and water [299]. While both excipients have been shown to be safe, literature holds a few reports about the hypersensitivity towards intravenous administration of mannitol [300,301,302]. Even in cases where mannitol has been used as an excipient in lower concentrations, hypersensitivity reactions have been still reported [291].

While sugar alcohols offer the advantage of safety and appropriate regulatory status, they often cannot provide the protein with adequate protection from all sources of stress, in particular the interface-induced stresses. As a result, these often need to be used in combination with a second, more surface-active excipient to ensure the adequate stability of the protein during all stages of production and storage. They can also provide a backbone for further chemical modification to reconcile the advantages of these molecules with the surface-active properties that can be provided through conjugation with other more lipophilic moieties.

- Cyclodextrins

According to the Inactive Ingredient Database of the FDA, four different types of cyclodextrins (CDs) have already been approved for use in pharmaceutical formulations. These include 2-Hydroxypropyl-γ-cyclodextrin (HP-γ-CD) for ophthalmic and topical formulations, α-cyclodextrin (α-CD) or Alfadex for liquid and solid intracavitary formulations, β-cyclodextrin (β-CD) or Betadex for oral, topical, intramuscular, and intravenous formulations, and sulfobutylether-β-cyclodextrin (SBE-β-CD) for intravenous and subcutaneous formulations [161]. CDs are cyclic oligosaccharides (Figure 3c) and the diversity in the ring size and in the sidechain of CDs confer them different properties that can be used for a variety of applications [303]. CDs are produced through the enzymatic degradation of starch and possess a hydrophilic outer surface and a hydrophobic cavity, rendering them water-soluble and biocompatible [304]. The most popular natural CDs are α, β, and γ, which contain 6, 7, and 8 glucopyranose units, respectively [304]. These are non-hygroscopic, crystalline, and homogeneous substances. Given their ideal cavity size for accommodating small molecules of the size of an amino acid, efficient drug complexation and loading, availability, and low cost, β-CDs provide an interesting candidate for the complexation with various drugs, mainly for the purpose of enhancement of the drug water solubility, improvement of the drug stability, increase of the drug dissolution rate and bioavailability, reduction of the side effects, and manipulation of the drug’s physicochemical characteristics [305]. To elaborate on the diverse role of CDs in drug delivery systems and the variety of CD-based systems for parenteral use is out of the scope of the current paper. For more information, the reader is referred to a well-structured review by Ferreira et al. (2022) for further information [306].

Interestingly, some CDs, e.g., HP-β-CDs (Hydroxypropyl-β-cyclodextrin) and Mβ-CDs (Methyl-β-cyclodextrins) have been shown to inhibit protein aggregation in a manner similar to non-ionic surfactants [307,308,309,310]. This can be either a by-product of the preferential adsorption of the CDs to the interface or else originate from the interaction of the CD molecules with the protein itself. It seems that both the nature and the concentration of the protein and the properties of the CDs determine which of the two aforementioned mechanisms will be dominant. Samra et al. (2010), who investigated the stabilizing effect of seven different CDs on three pharmaceutically relevant proteins, reported that almost all investigated CDs could successfully inhibit protein aggregation at a pH of 5.5. They argued that this potentially pertains to the characteristics (isoelectric point, molecular weight, and detailed tertiary structure) of the proteins, which in turn influences the nature of the interaction of the CDs therewith [311]. Concurringly, CDs have been shown to successfully stabilize rhGH through direct interaction therewith, for the unusually high proportion of solvent-accessible aromatic amino acids favors such interactions. It appears that stabilization due to the direct interaction between the CDs and the protein is often the dominant mechanism in case the protein has substantial solvent exposure to hydrophobic amino-acid residues [311]. Protein stabilization as a result of the preferential adsorption to the interface, however, is governed by the degree of the surface activity of the CD-derivative under investigation rather than the structure of the protein. For instance, HP-β-CD has been shown to reduce the interface-induced precipitation of porcine growth hormone [307]. HP-β-CD has also been shown to be a beneficial PS alternative for the protection of the IgG formulations from aggregation at the air–water interface [312,313].

As a result of the duality of the mechanisms involved in the CD-mediated protein stabilization, i.e., reduction of the protein surface adsorption and protein stabilization through direct interaction, it seems that a CD derivative beneficial for one therapeutic protein may have little effect on, or even jeopardize, the stability of another protein [314]. CD-derivatives that inhibit protein particle formation caused by one stress condition may not be able to do so in case of a different stress condition. Moreover, the required CD to protein concentration or molar ratio that would yield adequate stabilization differs greatly depending on the nature of both components. Thus, for the selection of CD-derivatives for the stabilization of biotherapeutic protein formulations, several factors should be heeded. The first is the stabilizing role that CDs are supposed to play in the formulation, i.e., “direct” interaction with the protein versus prevention of “surface/interface-induced” aggregation. Stabilization through the former may only be possible in certain cases, for which the presence of the highly solvent accessible exposed hydrophobic residues on the proteins might be an important but not compelling prerequisite [314]. The use of CDs to prevent surface-induced aggregation, on the other hand, may be possible in many cases, for which the surface activity of the CD-derivative must be taken into account [303]. The concentration of the protein might also play an important part. In solutions with lower protein concentrations, CDs might be able to better interact with the interface, leading to more efficient protection of the protein against interface-induced denaturation and particle formation. In case the protein concentration in the solution is high, however, the interaction of the CDs with the protein molecules might gain more relevance, which, as previously elaborated, varies significantly depending on the type of the CD and the presence of hydrophobic residues in the protein structure [311].

While CDs offer the benefit of chemical stability in biotherapeutic formulations, the concern of their enzymatic degradation remains to be investigated. A recent study by Zhang et al. (2022) investigated the chemical stability of HP-β-CD in comparison to PS20 and PS80. When subjected to heat stress, autoclave, light, and oxidative stresses, HP-β-CD remains almost stable in contrast to PS20 and PS80 [315]. However, the degradation of CDs can be triggered by various enzymes mainly from the glycoside hydrolase family 13, including cyclodextrinase, α-amylase, glycoamylase, and amylase belonging to the bacteria and archaea domain involved in the metabolism of carbohydrates [316]. Some of these are currently in use in CDs, and their derivates have shown low toxicity and resistance to the enzymatic degradation [317]. The saccharide nature of CDs endows them with physicochemical properties similar to dextrins. An outstanding property of CDs is their resistance against β-amylase due to the lack of susceptible non-reducing end groups. Conversely, α-amylases are capable of hydrolyzing the CDs from the carbohydrate chain as in starch, but to a lower extent [318]. The degree of hydrolysis is directly proportional to the size of the free CD moiety. By burying oxygen bridges inside the CD cavity, the resistance to hydrolysis increases [319]. Accordingly, α- and β-CDs are resistant to salivary α-amylase, while γ-CDs are sensitive to pancreatic and salivary α-amylase [320,321]. Furthermore, Aspergillus oryzae α-amylase, a fungal amylase mainly used for saccharide fermentation, has shown enzymatic activity towards γ-CDs, leading to the production of maltooligomers [322]. Additionally, two more α-amylase enzymes with cyclo-malto-dextrinase specificity have been identified in thermophilic bacterial strains [323]. It should be, however, noted that the majority of the identified CD-degrading enzymes are of bacterial origin, and hence will not create any issues in case the protein is purified from a eucaryotic host. In case of bacterial hosts, quantification of the CD degrading enzyme traces in HCPs and the determination of the enzymes’ affinity for the molecule is necessary to present a fair comparison between CDs and PSs in terms of their potential sensitivity to HCP-mediated degradation.

CDs can also be used in combination with PSs to help mitigate their limitations. In an interesting patented work, Connolly et al. (2017) demonstrated that the use of HP-β-CD, together with PS20, improves the stability of the latter towards enzymatic degradation. This was confirmed based on the decrease of the sub-visible particle formation in PS-containing solutions in the presence of different HCPs. HP-β-CD has also been shown to protect PS20 in the presence of several enzymes (phosphotriesterase-like-lactonase, Candida antarctica lipase B, renin-like enzyme, and lipoprotein lipase) in a concentration-dependent manner [324]. It has been hypothesized that such protective effects might originate from the “direct” interaction of the CD with PS20, leading to the formation of an inclusion complex. This would in turn decrease the interaction between the host cell enzyme traces and the PS20, thereby protecting the latter against degradation. Furthermore, it is described that CDs can help solubilize the sub-visible particles originating from the enzymatic degradation of the PS20 [324]. Accordingly, using a combination of CDs and PSs for the stabilization of protein biopharmaceutics can be beneficial. An improved stability of the PS surfactants will help overcome some of the problems originating from the PS degradation, including the potential damage to the protein structure as well as the sedimentation of visible or sub-visible FFA particles. It should be, however, noted that combined use of CDs and PSs would increase the complexity of the formulation. Moreover, CDs might disturb established analytical methods to detect surfactants like PSs.

In addition to the enzymatic degradation, the oxidative cleavage of CDs is another under-investigated potential instability mechanism for these excipients [325]. Within this context, the presence of OH-radicals generated by auto-oxidation processes can lead to the cleavage of glucosidic bonds of β-CD, producing thereby different oligosaccharides like D-xylose, D-threose, D-arabinose, and D-erythrose [325].

Overall, there seems to be a need for more in-depth research to provide further information regarding the ability of the CDs to stabilize protein formulations. Furthermore, the degradation profile of the excipient, along with its consequences for the quality of the final product, particularly upon long-term storage, should be examined. The available information at this point depicts a promising picture, where CDs, alone or in combination with PSs, can help overcome the drawbacks of the latter in the formulation of injectable protein biotherapeutics.

- Hydroxypropyl methylcellulose

Hydroxypropyl methylcellulose (HPMC) (Figure 3d) is a semi-synthetic cellulose ether, well-known for its use as a pharmaceutical excipient in oral dosage forms [326]. HPMC is a unique excipient in the sense of the malleability of its physicochemical characteristics, which supports the molecule diverse applications. Within this context, HPMC has been used for the formulation of mucoadhesive systems for the delivery of small molecules, as an excipient in amorphous solid dispersions to enhance the bioavailability of poorly water-soluble drugs, or as a component of various nanocarrier formulations [327,328]. HPMC and other cellulose derivatives have been shown to be non-toxic following pulmonary, oral, intraperitoneal, subcutaneous, or dermal application [329]. These have been shown to be non-irritating to mildly irritating, non-sensitizing, and non-mutagenic in clinical studies [329].

Interestingly, HPMC has been tested as a stabilizer for mAb formulations [330]. In one study, different cellulose polymers, including methylcellulose, HPMC, hydroxypropyl cellulose, and hydroxyethyl cellulose, were used to stabilize 1 mg/mL cetuximab. Among these, low molecular weight HPMC could efficiently stabilize the Ab, with an efficiency comparable with PS80 [330]. Similarly, the ability of low molecular weight HPMC to protect abatacept has been demonstrated [330].

HPMC is classified as GRAS, included in the FDA Inactive Ingredients Database for use in ophthalmic and nasal formulations, oral capsules, suspensions, syrups, and tablets, and listed in the Canadian list of acceptable non-medicinal ingredients [161,291]. A high oral dosage of HPMC has been evaluated for the treatment of postprandial insulinemia [329] and metabolic syndromes such as hypertension, proinflammatory or inflammatory state, prothrombotic state, atherogenic dyslipidemia, and insulin resistance [331].

Based on the above-debated information, more profound research is worth being dedicated to the investigation of the ability of HPMC to stabilize protein formulations, degradation of the molecule, and the safety of the excipient following intravenous administration.

- Dextrans

Dextrans (Figure 3e), complex branched glucans, are another group of carbohydrate-based polymers capable of protein stabilization through molecular crowding effect [332]. Dextrans can reduce the adsorption of the proteins to the liquid/air interface [53], thus preventing the aggregation and denaturation thereof in liquid formulations. They can also help maintain protein structural integrity during freezing, drying, and storage in the solid state [333]. Within the context of the last point, the molecular weight of dextran is a determining factor for the extent of protein stabilization. In a study comparing the efficiency of dextrans with different molecular weights ranging from 12 kD to 2000 kD on the structural stability of freeze-dried protein, the medium molecular weight of 512 kD was shown to be most effective [334]. The reasons behind the differences in protein stability among various molecular weight dextrans remain unknown [334].

The drawback of dextrans for protein stabilization lays in their tendency to form polymerprotein aggregates. This occurs through a Maillard reaction between the protein’s primary amines and dextran’s reducing ends. Typically, these reactions are observed following storage at temperatures over 50 °C within the course of days. For some proteins, such as those of blood origin, the reaction can occur even following around 6 months of storage at room temperature or a month at 37 °C. Dextranol, a form of reduced dextran, has been shown to solve this problem, thereby serving a more efficient lyoprotectant [335].

Dextrans are FDA-approved and already part of several FDA-approved injectable formulations. However, based on our literature search, there have been a few reports of side effects in patients. For instance, infusion of dextran 40 (average molecular weight: 40,000 D) has been reported to induce renal diseases in patients with reduced urine flow. Infusion of dextran 70 (average molecular weight: 70,000 D) might induce hypersensitivity reactions, such as urticaria, hypotension, and bronchospasm, and milder reactions like fever and nasal congestion. Despite not being so common, anaphylactic reactions are still possible when dextran is administered [294]. In fact, a case of anaphylactic reaction to dextrans as a component of the BCG (Bacillus Calmette–Guérin) vaccine and remimazolam formulation has been reported [336,337].

Despite the potential benefits, it is unclear whether the use of dextrans alone can confer adequate stability to the protein biopharmaceuticals. Here again, the polymers can be used, potentially in combination with a more surface-active excipient, as potential PS alternatives, or else serve as a backbone for further chemical modifications to enhance their surface-active properties.

### 3.3. Amino Acid-Based Stabilizers

- Amino acids

Certain amino acids and their derivatives can inhibit protein aggregation and promote protein refolding. Many of these are neutral osmo-protective molecules, e.g., proline [338], taurine [339], glycine [340], arginine [341], etc. The mechanism behind this phenomenon is rather diverse depending on the type of amino acid and the protein in question. As a detailed discussion of these is out of the scope of the current paper, the reader is referred to an elegant review by Mojtabavi et al. (2019) for further information [342]. While the effect of amino acids upon protein stability seems to be protein dependent, arginine has been proposed as a “universal” protein stabilizer and aggregation suppressor. Arginine has been long since shown to reduce the thermally and dilution-induced particle formation of proteins such as lysozyme [341,343], recombinant plasminogen activator (rPA) [343], and lactoferrin. Another advantage of arginine is its ability to decrease the viscosity of liquid formulations, as is the case for highly concentrated BSA (275 mg/mL) [344], bovine gamma globuli (250 mg/mL) [345], and human gamma globulin (292 mg/mL) [345]. The molecular mechanisms through which arginine fulfills this are poorly understood. In solution, arginine molecules tend to self-associate [346]. These supramolecular assemblies provide a hydrophobic environment, which might interfere with the hydrophobic association of the proteins, thus preventing protein aggregation [346]. Furthermore, the presence of the carboxyl group in the arginine structure ensures enough interaction with the protein to enable the stabilization of the partially unfolded intermediates, but not so strong to induce protein unfolding [347]. Within the course of dehydration during freeze-drying, protonated arginine can provide protein stability through the formation of effective bonds therewith, partially attributable to the additional hydrogen binding of the protonated side chain of the amino acid. It may also be favored by ion-dipole interactions between the protein and the cationic charge of the amino acid [348]. Other amino acids, such as histidine and lysine, which behave like arginine by virtue of comprising a cationic sidechain in their structure, can also serve as effective stabilizers of mAbs [349]. Besides arginine, histidine, which is primarily used as a buffering agent [350], has been reported to stabilize mAbs as well.

A more recent publication demonstrated that arginine could improve the stability of high concentration protein solution. Here, Bramham et al. (2021) compared high concentrated mAb formulation (171 mg/mL) with and without 100 mM arginine after storage at 4 °C and 40 °C for 4 weeks. Antibody aggregation was not found in samples stored at standard refrigerated conditions (4 °C) but under stressed conditions (40 °C). However, the formulation with 100 mM arginine was more stable and displayed less antibody aggregation at 40 °C compared to the formulation without arginine [351].

Amino acids are GRAS and FDA-approved for use in different drug products, including injectable formulations [352]. A thorough assessment of the safety of different amino acids for cosmetic use is published [352]. Many amino acids are available as sterile solutions for use in pharmacy admixture programs for the preparation of intravenously injectable fluids. Among these, however, the safety of arginine is most widely investigated, as it is already marketed as R-Gene^®^ 10 for direct intravenous infusion. The product contains 10 g arginine hydrochloride to stimulate the pituitary for the release of hGH [353]. Intravenous infusion of R-Gene^®^ 10 in 1670 patients has been well-tolerated. With the exception of single case reports, the only prevalent adverse effect reported by 3% of the patients has been non-specific side effects such as nausea, vomiting, headache, flushing, numbness, and local venous irritation [353]. In one study, intravenous infusion of 30 g L-arginine, as well as oral intake of 10 g L-arginine in healthy subjects was shown to be well-tolerated [354]. Within this context, no significant change in the vital signs and no adverse reactions was observed [354]. On the other hand, 30-min-long infusion of 30 g of L-arginine in healthy subjects has also been shown to reduce peripheral arteriolar tone and inhibit platelet aggregation through the increase in nitric oxide formation [355]. According to the data submitted to the European Chemical Agency (ECHA), orally administered LD_50_ of arginine in male and female Wistar rats has been greater than the administered dosage, i.e., 5110 mg/kg body weight [356]. Tsubuku et al. (2004) performed cytotoxicity studies with rats to evaluate the toxicity of arginine when given at concentrations of 1.25%, 2.5%, and 5% *w*/*w* for a period of 13 weeks, followed by a 5-week-long recovery phase [357]. The results showed no significant impact of L-arginine upon parameters such as body weight, organ weight, as well as histopathology and ophthalmology indicators. In the group having received the highest dose (5% *w*/*w*), an elevated blood glucose level, though still within the physiological range, as well as an increase in hemoglobin, along with a tendency toward increased erythrocyte counts was observed in some animals [357]. According to a literature review presented in document provided by the FDA Center for Drug Evaluation and Research, L-arginine has no adverse effect on animal reproduction, is not genotoxic, and is not expected to be carcinogenic, given that it is a naturally occurring amino acid [358]. The same document reports the safe level of arginine oral intake in healthy adults to be about 20,000 mg/day [358].

In conclusion, being safe and FDA-approved for parenteral administration, amino acids provide viable options as stabilizers of protein formulations. However, they are associated with the drawback of the complexity of their effect upon different proteins in various biotherapeutic formulations. Accordingly, the type and concentration of the stabilizing amino acids for each specific protein formulation must be individually optimized.

- Natural polyamines

**Figure 4:**
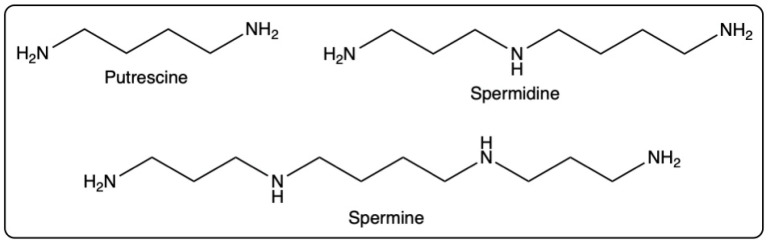
Structures of low molecular weight polyamines used in the prevention of heat-induced aggregation and inactivation of proteins.

Low molecular weight aliphatic polycations, polyamines, are ubiquitous highly charged molecules synthesized from arginine, ornithine, and methionine [359]. Naturally occurring polyamines have been demonstrated to prevent the heat-induced aggregation and inactivation of proteins [360]. Kudou et al. (2003) reported the efficiency of spermidine and spermine polyamines (Figure 4) on the aggregation inhibition of lysozyme heated at 98 °C for 30 min, though 50% of the protein had lost its activity as a result of severe thermal stress [360]. Putrescine, a third polyamine (Figure 4), managed to prevent aggregation under the same conditions, although it resulted in a preservation of a mere 15% of the lysozyme residual activity. Under identical conditions, Okanojo et al. (2005) investigated the efficiency of different monoamines and diamines in terms of lysozyme aggregation inhibition [361]. Unlike polyamines, monoamine compounds were not able to prevent lysozyme thermal aggregation. Diamines, on the other hand, yielded better results compared to monoamines [361]. It should be noted, however, that the stabilizing effect of polyamines has been only established in case of formulations with low protein concentrations (0.2 mg/mL). Accordingly, their ability to prevent protein inactivation and particle formation in HCLF needs to be further investigated. Furthermore, whether these can protect protein integrity in face of stress factors other than the thermal load is yet to be explored. Finally, the investigation of the safety and tolerability of these stabilizers is another vital aspect that requires more detailed investigation. In one study, the safety and tolerability of orally administered spermidine from plant extract in mice and older adults was confirmed [362]. In a different study, Til et al. (1997) investigated the acute and subacute toxicity of 5 different polyamines, namely tyramine, spermidine, spermine, putrescine, and cadaverine, in Wistar rats [363]. They established an oral acute toxicity dose of 2000, 600, and 600 mg/kg body weight for putrescine, spermidine, and spermine, respectively [363]. In subacute toxicity studies, spermine was the most toxic when given at the highest possible concentration. Based on these studies, the no-observed adverse effect level (NOAEL) for the investigated polyamines following oral administration was reported to be 2000 ppm (180 mg/kg body weight/day) for putrescine, tyramine, and cadaverine, 1000 ppm (83 mg/ kg body weight/day) for spermidine, and 200 ppm (19 mg/kg body weight/day) for spermine [363]. The study also cast a look on the effect of intravenous injection of these polyamines upon the blood pressure of the rats. The results demonstrated that apart from tyramine, all polyamines resulted in dose-dependent hypotension.

- Albumin

Albumin, the most prevalent plasma protein, is an effective stabilizer for protecting other proteins from denaturation and aggregation [364]. This is partially governed by the ability of albumin to competitively inhibit protein adsorption to the interfaces [365], as the methyl and carboxylic groups of the protein interact with hydrophobic hydrophilic phases, respectively [366]. Furthermore, the chemical structure of albumin enables the protection of other proteins through hydrophobic and electrostatic interactions [367], as well as through intermolecular structure formation [368].

By virtue of the above-debated, and based on its favorable safety profile, albumin has been used to stabilize different interferon (IFN) formulations. Commercially available examples include IntronA^®^ and Betaseron^®^, where albumin has been exploited to prevent or reduce particle formation [369]. Usually, higher concentrations of the human serum albumin (HSA) are used in the case of IFN-β formulations, as these have higher aggregation tendencies compared to the IFN-α products [369]. As albumin has been shown to have antioxidant properties, it might also prevent or mitigate protein oxidation [370].

Constituting more than 50% of the plasma proteins, albumin is safe for parenteral use, is non-toxic, and has non-irritant properties. It is included in the FDA Inactive Ingredients Database for intravenous injections and infusions, subcutaneous injectables, nasal formulations, and oral dosage forms, and is also listed under the Canadian list of acceptable non-medicinal ingredients [161,291]. Anaphylactic reactions are possible, but not so common. It might, however, lead to minor side effects such as urticaria, skin rash, nausea, vomiting, chills, and febrile reactions [291].

Notwithstanding the listed benefits, the use of albumin in protein formulations is fraught by several drawbacks. The first is well-known in the case of IFN formulations, where the molecular interactions between the two proteins can lead to the formation of albumin–IFN aggregates. These aggregates are currently the paramount suspects behind the immunogenicity of IFN formulations [23]. One strategy to overcome this issue has been the design of a genetic fusion protein, albinterferon-α-2b. This albumin derivative has shown considerable stability towards stress-inducing conditions such as agitation and high-temperature storage [371].

While HSA remains an established stabilizer for the improvement of the storage stability of lyophilized protein and mAb formulations [256,367], it is also associated with the concern of immunogenicity and blood-transmitted diseases [256]. Being of animal origin, the same concerns also apply to BSA [256]. An approach to tackle this has been the design of recombinant albumin. In one early study, Tarelli et al. (1998) compared the ability of recombinant albumin with HSA in terms of stabilizing three lyophilized preparations containing thyroid-stimulating hormone, interleukin-15 and ranulocyte colony-stimulating factor [372]. The findings confirmed the comparable ability of the recombinant albumin to HSA in terms of preserving the immunological, biological, and biochemical properties of all three proteins [372]. Furthermore, both forms of albumin showed similar physicochemical characteristics, e.g., similar binding affinity to fatty acids [372]. Hence, recombinant albumin can provide a viable excipient for the stabilization of protein biologicals. It has to be mentioned, that the exact formulation composition, of commercially available recombinant albumin is barely disclosed.

### 3.4. Synthetic Amphiphilic Polymers

Another group of potential excipients for protein stabilization includes the synthetic amphiphilic polymers. These are debated here separately from the surfactant polymers considering their limited surface-active properties in comparison to the latter group. The advantage of synthetic polymers lies within the modifiability of their chemical structure, molecular weight, and functionality, depending on the requirements of the formulation. Some of these are already used as excipients in intravenously injected formulations, while others still lack an acceptable regulatory status.

- Polyether polyols

**Figure 5:**
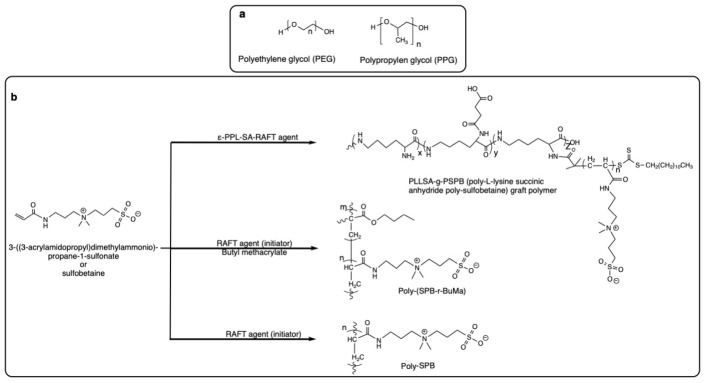
Chemical structures of synthetic amphiphilic polymers with potential to serve as PS alternatives: (**a**) Polyether polyols, (**b**) Sulfobetaine and its derivatives as examples of polyampholates.

The most famous members of this category are PEGs (Figure 5a), which have also been included in the structure of various non-ionic surfactants. Chemical conjugation of PEGs to various drugs is a highly sought-after method to enhance the drug solubility, improve the drug stability, and prolong the drug’s residence time in the systemic circulation. Proteins have been no exception, and different PEG–protein conjugates have been developed thus far. Chemical conjugation of the PEG moiety to therapeutic proteins can reduce the aggregation of the latter, probably attributable to the steric shielding of the protein’s superficial hydrophobic patches [373]. Short-chain PEGs have been shown to even protect the protein intracorporeally, reducing its proteolytic degradability either through interacting with the protein surface or else extending into the solvent [374]. Notwithstanding, the covalent binding of the PEGs to the proteins is associated with the disadvantage of requiring chemical processing that can potentially influence the proteins’ biological activity. It also mandates subsequent additional steps, including characterization and purification. Fortunately, to protect the protein integrity, PEGs do not necessarily need to be covalently bound thereto. When used in optimal molar ratios, free PEGs of a higher molecular weight have been shown to maintain the thermal stability of BSA and urokinase during lyophilization and thermal stress, respectively [375,376]. However, considering the significantly lower surface activity of PEGs compared to PS surfactants, the efficiency of the polymer to reduce monomer loss or particle generation is inferior to the latter. It also shares the drawback of sensitivity to auto-oxidation [44]. A third drawback of PEGs and PEG-containing molecules, that has been previously elaborated on, is their tendency to induce mild to life-threatening immunogenic and hypersensitivity reactions [226]. Combined, these drawbacks highlight the necessity of searching for more suitable polyether polyols with potential to serve as PS alternatives.

Poly (propylene glycols) (PPGs; Figure 5a) is another polyether polyol exploited for the stabilization of protein formulations. A patent published by Reform Biologics (2019) investigated PPGs of different molecular weights (PPG 425, 1000, 2000) in terms of their ability to stabilize a solution of 2 mg/mL cetuximab exposed to continuous shaking stress (275 rpm) for 16 and 40 h at ambient temperature. The results demonstrated a comparable performance of PPGs to PS80 and a superior effect compared to PEG 1000 and HPMC [330]. In general, PPGs have been shown to possess higher surface-activity than PEGs. For instance, the surface tension of PEG and PPG hydrogels with a bulk concentration of 1.5 g/L has been measured as 36.58 and 33.01 mN/m, respectively [377]. Nonetheless, the protein stabilization of the PPGs is hypothesized to be a function of direct interaction with the protein molecules rather than interfacial competition [44]. From a safety perspective, PPGs are also FDA-approved excipients, mainly used in oral and topical formulations. Combined, these characteristics raise hope that PPGs might be viable PS alternatives for protein stabilization. Nevertheless, two issues need to be addressed in respect to these excipients. The first issue relates to the stability of these polymers, and whether they are also prone to undergoing auto-oxidative reactions. The second issue is the potential immunogenicity of the polymer. To our best of knowledge, no reports of immunogenicity and hypersensitivity to PPGs are currently available. However, cross-reactivity between anti-PEG Abs and PPGs has been observed [378]. Thus, a thorough investigation of the stability and immunogenicity of these polymers inspires further research.

- Polyampholytes

Polyampholytes are a subclass of zwitterionic materials that contain opposite charges on different monomer subunits [379]. An example of these materials is the sulfobetaine-based polymers (Figure 5b). Rajan et al. (2017) reported poly-sulfobetaine (poly-SPB) to suppress thermal aggregation of lysozyme as a model protein [380]. The polymer showed a good protective effect against conformational changes and helped retain more than 85% protein activity following exposure to elevated temperatures. Furthermore, the zwitterionic polymer has been shown to suppress the aggregation-induced collision within the misfolded protein hydrophobic domains [380]. Employing the same sulfobetaine backbone, poly-SPB was copolymerized with a hydrophobic monomer, butyl methacrylate, to produce copolymers with variable degrees of hydrophobicity [381]. The polymer was used to stabilize insulin upon exposure to 37 °C for 24 h. The results demonstrated no turbidity, which is often observed in inadequately stabilized formulations after 7 h of incubation. Another zwitterionic polymer with similar properties was synthesized by Zhao et al. (2019), where a poly-SPB was grafted to an ε -poly-L-lysine-succinic anhydride carboxylated backbone [382]. Compared to the unmodified poly-SPB, the grafted polymer yielded better thermal aggregation inhibition of lysozyme [382]. These synthesized polymers have shown promising protein particle formation inhibition at elevated temperatures. However, their chemical stability, safety, and suitability for parenteral biotherapeutic formulations remains uncovered.

### 3.5. Ionic Liquids

Organic salts with low melting points (typically < 100 °C) and often liquid at room temperature, ionic liquids have recently gained remarkable interest for the stabilization different proteins [383]. These are mostly made of an asymmetric organic cation in combination with an organic or inorganic anion. Considering the million possible anion/cation combinations, the properties of ionic liquids are highly tunable [383]. Ionic liquids are often classified as protic and aprotic, with the base being quaternized by protons in case of the former and an alkyl group in case of the latter [384]. Accordingly, protic ionic liquids often serve as hydrogen bond donors, whereas their aprotic counterparts act as hydrogen bond acceptors [383]. Ionic liquids can be exploited to improve various stages of the development of protein formulations, including protein extraction, separation, crystallization, solubilization, and stabilization [385]. Within the context of protein stabilization, ionic liquids have been both investigated as neat solvents and as co-solvents in aqueous solutions [383]. However, considering the high viscosity of the ionic liquids, their hypertonicity in the neat form, their limited ability to dissolve high concentrations of proteins, and the reduced activity of many proteins (enzymes) therein, the use of ionic liquids as cosolvents for protein stabilization in aqueous solutions has been more widely sought after [383,386]. Depending on the anion charge density as well as the presence or length of an alkyl group on the cation, and perhaps the anion, ionic liquids can behave in a hydrophilic or a hydrophobic manner in aqueous solutions [383].

Literature holds many favorable reports confirming the ability of different ionic liquids in protein stabilization. For instance, ammonium-based ionic liquids have been shown to increase the denaturation temperature of insulin, and to prevent the aggregation and increase the amount of the monomeric, i.e., the active form of the protein [387]. Furthermore, they have been shown to induce refolding and renaturation of chemically unfolded lysozyme [388,389]. Summers and Flowers (2000) have proposed the protein stabilization mediated by the ammonium-based ionic liquids to originate from both the interaction of their hydrophobic groups with the hydrophobic sections of the protein and the subsequent inhibition of intermolecular association thereof, as well as the ability of the charged portion of the compound to stabilize the protein’s secondary structure [388]. Ammonium-based ionic liquids have also been shown to protect proteins against thermal stress [390]. For instance, ethylammonium formate (EAF), 2-methoxyethylammonium formate (MOEAF) and propylammonium formate (PAF; up to 62.5% *w*/*w*) have been shown to effectively induce lysozyme refolding following exposure to a temperature of 90 °C, while ethanolammonium formate (EtAF) could completely protect lysozyme against thermal-induced denaturation under the same conditions [390]. A second group of ionic liquids, imidazolium-based salts can also exert a stabilizing effect on some proteins under the right conditions [383]. These have been shown to successfully preserve the native form of lysozyme and to increase the melting point of the enzyme, thereby improving the protein’s thermal stability [391]. The formulation of horse heart cytochrome C in 1 mM aqueous solution of 1-methyl-3-octyl imidazolium chloride and 1-decyl-3-methylimidazolium chloride could maintain the stability of the protein for up to 6 months when stored at room temperature [392]. Similarly, studies have indicated that N’-alkyl and N´-(ω-hydroxyalkyl)-N-methylimidazolium chlorides can facilitate the refolding of lysozyme and single-chain antibody fragment, although destabilization was observed with increasing salt concentration [393]. As a last example, 1-ethyl 3-methyl imidazolium ethyl sulfate and 1-ethyl 3-methyl imidazolium chloride could enable the refolding of chemically denatured serum albumin [394].

While the ability of some ionic liquids in the stabilization of certain proteins has been demonstrated, the underlying interactions are yet to be fully understood [384]. This is in part due to the structural diversity of the proteins and ionic liquids, and is further complicated by the presence of water, which forms hydrogen bonds with both components [384]. Constatinescu et al. (2010) have demonstrated that, depending the nature of the ions, ionic liquids can have both stabilizing and destabilizing effects on RNase A as a model protein [395]. They have also demonstrated that regardless of their stabilizing/destabilizing abilities, all tested ionic liquids were able to inhibit protein aggregation when used in the “right concentration” [395]. A detailed discussion of the why behind the controversial stabilizing/destabilizing effect of ionic liquids on different proteins is out of the scope of the current paper. For detailed information, the reader is referred to an elegant review by Zhao (2016) [396]. In general, however, the stabilizing/destabilizing effect of ionic liquids on different proteins seem to originate from and intricate interplay of various properties of these solvents, including their hydrogen binding capacity, log P, nature of the anions (the Hofmeister ion series) and cations, and the alkyl chain length of the cation [385]. One of the most important factors is the concentration of the salt, where higher concentrations lead to more intense interactions with the protein, which facilitates the destabilization of the latter [393,397,398]. Bisht et al. (2015) demonstrated that the protein stabilizing effect of ammonium-based ionic liquids against thermal stress is reversely correlated with the concentration of the salt, as in the presence of highly concentrated ionic liquids the strong interaction between the ions and the amino acid residues of the protein becomes highly favorable [397]. In addition to the concentration, the type of the anion is also determinant of the outcome of ionic liquids’ interaction with the proteins. Kumar et al. (2014) observed that short-chain imidazolium salts based on Br^−^ and Cl^−^ stabilized insulin, whereas anions such as SCN^−^, HSO_4_^−^, CH_3_COO^−^, and I^−^ acted as denaturants [399]. Similar findings have been reported by Kumar et al. (2017), who established the efficiency of imidazolium salts in stabilizing stem bromelain to be in the order of Cl^−^ > Br^−^ > I^−^, which is in accordance with the Hofmeister series [400]. The length of the alkyl group on the cation is also a determining factor. For instance, the loss of the activity of adenosine deaminase was significantly higher in the presence of the more hydrophobic 1-octyl 3-methyl-imidozolium chloride than the less hydrophobic shorter-chain 1-allyl 3-methyl-imidazolium [401]. Similarly, short- and medium-chain imidazolium-based ionic liquids performed superior to their long-chain counterparts in terms of preserving the stability of green fluorescent proteins [402].

Despite the promising findings supporting the potential of ionic liquids for efficient protein stabilization, the application of these as excipients in biotherapeutic formulations requires addressing a number of issues. First and foremost, the controversial effect of ionic liquids on the stability of different proteins necessitates the screening of potential protein–ionic liquid pairs. This requires suitable analytical methods for the prediction of the stabilization/destabilization outcome in the pre-formulation step. To this date, different techniques have been used to examine the effect of ionic liquids upon the stability of various proteins. These include ultraviolet-visible (UV-Vis) spectroscopy, far and near UV circular dichroism, Fourier-transform infrared spectroscopy, Raman spectroscopy, intrinsic tryptophan fluorescence, DLS, small-angle X-ray scattering, small angel neutron scattering, tensiometry, microcalorimetry, and nuclear magnetic resonance (NMR) [385]. Cheng et al. (2019) have proposed ^19^F NMR as a simple analytical technique to study thermodynamics of the protein stability and protein–protein interactions in ionic liquids [403]. However, Bui-Le et al. (2020) have argued that considering the complex nature of the interactions between different ionic liquids and proteins, a single technique investigation is potentially not conclusive, and a multi-technique analytical framework is needed to paint a complete picture of the underlying interactions [386]. The multi-technique analytical framework proposed by the authors included a combination of circular dichroism, fluorescence, UV-Vis spectroscopy, NMR spectroscopy, and small-angle X-ray scattering [386].

The second issue pertains to the isotonicity of these formulations. Isotonicity is one of the important characteristics of parenteral formulations to be strived towards [404]. Should protein stabilization require high ionic liquid concentrations (e.g., >50% *w*/*w*), issues related to the hypertonicity of the final formulation will be unavoidable [383]. This might be an issue, even when ionic liquids are used in lower amounts [405]. For instance, a formulation containing 12% *w*/*w* choline dihydrogenphosphate has an osmolality of about 1520 mOsm/kg [405]. This level of osmolality is far beyond the near physiological levels (300–600 mOsm/kg), which renders this formulation questionable for intravenous bolus injection [405]. One potential approach to overcome such problems is to consider diluting the preparation before administration, or to infuse the formulation with an adjusted rate [383,406]. Alternatively, other routes of administration can be considered. For instance, the intramuscular administration of 0.5 mL of a hypertonic formulation with an osmolality up to 1100 mOsm/kg has been reported to be well-tolerated [406].

A last, but highly essential, issue with the use of ionic liquids is their under-investigated toxicity and biocompatibility in humans [407]. To this date, the toxicity of ionic liquids for bacteria, fungi, plants, and certain small animals such as *Danio rerio* (zebra fish), *Mytilus galloprovincialis*, *Artemia salina*, *Caenorhabditis elegans,* and *Galleria mellonella* has been investigated and elegantly reviewed by Gonçalves et al. (2021) [408]. Currently, the only available data regarding the toxicity of ionic liquids for humans are those obtained based on cell culture studies on HeLa, CaCo-2, and HT-29 cells [409]. The results are indeed dependent on the type of ionic liquid used for these experiments, as well as the cell line under investigation [409]. In a comparative study, the cytotoxicity of 31 different ionic liquids for normal human dermal fibroblasts was investigated [410]. The results showed imidazolium-based ionic liquids combined with dialkyl phosphate anions or with the ethyl sulfate anion to have the lowest toxicity [410]. A different study comparing the toxicity of eight different ionic liquids, including imidazolium-based salts, established the choline-based ionic liquids as the type with the lowest toxicity for HeLa cells [411]. In general, the findings of toxicological studies seem to point out that the toxicity of ionic liquids mostly pertains to the toxicity of the anionic and cationic precursors used for the synthesis and which remain as impurities in the ionic liquids [412]. The most important factors within this context are the length of the alkyl chain, as well as the branching and hydrophobicity of the cation [412]. By decreasing the hydrophobicity of the side chain through the addition of hydrophilic functional groups (e.g., hydroxyls, nitriles, and polar ethers), their interaction with the cell membrane and the subsequent cytotoxicity of these solvents will be reduced [412]. Moreover, the increase in the size of the anion can increase the toxicity of the ionic liquids, as demonstrated by Weaver et al. (2010), who compared the toxicity of choline-based ionic liquids containing five phosphate anions of varying sizes [413]. In all, the increasing knowledge of the factors determining the toxicity of ionic liquids raises hope that by choosing biocompatible, biodegradable, and non-toxic cations and counter anions, a new generation of safe, non-toxic, biocompatible, and even biodegradable ionic liquids can be synthetized [412]. Until then, the potential of ionic liquids to serve as PS alternatives in protein therapeutic formulations will remain limited.

## 4. Discussion

Recent decades have witnessed a rapid rise in the development of protein biotherapeutics for the treatment of various disorders, as these provide higher specificity and lower side effects when compared to small molecular drugs in many cases. The formulation of HCLF (high concentration liquid formulations), however, is fraught with unique complications in terms of maintaining the stability of the therapeutic protein. Under such conditions, a decrease in the protein solubility, along with the increase of the formulation viscosity, can threaten the colloidal and conformational stability of the protein therapeutics. Protein denaturation and aggregation are also promoted through protein adsorption to various interfaces, as well as through shear and thermal stresses during different stages of production, packaging, storage, transportation and even administration. As gold standards for the stabilization of the protein biotherapeutics, PSs (polysorbates) are prevalently used in the pharmaceutical industry given their compatibility with most bioactives, their ability to prevent protein aggregation and surface adsorption, and their efficiency in protecting the protein from different stress conditions. According to one of the latest reviews from Strickley et al. (2021), there are 131 commercially available antibody formulations. From these, PS80 is contained as the main surfactant in 82 of the commercialized formulations, followed by PS20, which is contained in 32 commercialized formulations, poloxamer 188 (P188) in only 4 solution formulations, and macrogol in only 1 product [160,234]. The remaining 12 commercialized products do not contain a surfactant [234]. To do a comparison between surfactants with other excipients regarding their presence in commercially available antibody formulations is not possible, mainly because other excipients might have been used to fulfill a different role in the formulation. For example, the main excipients used to adjust tonicity or osmolality and to serve as lyoprotectants are from the carbohydrate families, and their order of occurrence in approved biological products is sucrose > trehalose > sorbitol > mannitol [234]. Although used as the most prevalent non-ionic surfactant, PS has been associated with several drawbacks, mainly the susceptibility of the ester bond to chemical and HCP-mediated (host cell proteins) enzymatic cleavage [74,109,111,122,128,129], as well as the photo- and auto-oxidation, and the subsequent cleavage of the ethylene oxide subunit [68,70,73,107,116,136]. Thus, PS degradation can culminate in protein denaturation, an increase in the number of visible or sub-visible particles, and even undesirable mild to severe immunogenic reactions. Hence, the search for excipients that can offer the advantages of PSs while being devoid of their shortcomings is still ongoing. Novel excipients which have not been previously used in FDA-approved drug products or in the food industry can be proposed by excipient manufacturers to the Novel Excipient Review Pilot Program. This program will address the lack and foster the development of excipients.

Excipients with the potential to serve as PS alternatives belong to diverse classes. These can be surfactants, carbohydrate-, and amino acid-based excipients, synthetic amphiphilic polymers, or perhaps ionic liquids. Table 2 presents a summary of the advantages and limitations of each class as potential PS alternatives in protein biotherapeutic formulations.

To serve as a suitable PS alternative, the candidate excipient must have several characteristics. In the first and foremost place fall three different independent properties, which are equally important for the selection of a suitable excipient. One is the ability to efficiently stabilize the protein against thermal and mechanical stresses in amounts that are realistic from a formulation development point of view. The second is, of course, the safety profile of the excipient for parenteral and particularly intravenous administration and commercial GMP-availability for parenteral applications, and finally, the stability of the excipients under storage conditions during the product’s shelf-life. These three characteristics provide a chicken and egg scenario, where an excipient with good protein stabilization efficiency but undefined or suboptimal toxicity profile is not suitable for pharmaceutical application, and yet toxicological evaluations are not worth being carried out on molecules with limited protein stabilization efficiency. The best solution to this controversy is perhaps to investigate the protein stabilization efficiency of the excipients with already-established safety profiles. Ideally, the excipient should be approved for parenteral and, in particular, intravenous administration. However, excipients hitherto approved for other routes are also worth considering, as their further investigation for parenteral administration is easier to carry out.

In terms of protein stabilization efficiency, surfactants are the most promising candidates, as they can universally compete with proteins for various interfaces and inhibit particle formation through direct interaction with certain proteins. For some excipients, the efficiency to stabilize certain proteins has been compared to that of PS20 or PS80 (Table 2). Table 2 provides a summary of the findings of the studies dedicated to this subject. As observed, at least several excipients such as Kolliphor^®^ HS15 [204], Kolliphor^®^ EL [204], Brij^®^ 92 [247], and Brij^®^ 58 [249] have exhibited comparable, or in some cases, even superior protein-stabilizing efficiency to PSs, rendering them as viable candidates for the debated purpose. Some non-surfactant excipients, such as HPMC (Hydroxypropyl methylcellulose, have also been shown to stabilize certain proteins with an efficiency comparable with that of PS80 [330]. The ability of other non-surfactant excipients to protect proteins is rather diverse. Disaccharides and sugar alcohols are better known for their thermal and cryoprotective effects, and often need to be combined with excipients with higher surface-active properties. Others, such as albumin, are efficient protectors against both stress conditions. Amino acid excipients and ionic liquids can both stabilize and destabilize proteins depending on their nature, concentration, and the structure of the protein in question.

Within the context of the safety profile, surfactants such as Kolliphor^®^ HS15, Kolliphor^®^ EL, P188, and other excipients, including albumin, amino acids, disaccharides, sugar alcohol, HP-β-CD (hydroxypropyl- β-cyclodextrin), and dextrans, are most promising as they are already approved by the FDA for parenteral administration. Others including PEG (polyethylene glycol) 2, 40, 80, and 100 stearates, Brij^®^ 78 and 98, sucrose stearate and sucrose palmitate, and other types of CDs possess acceptable regulatory status for use in non-injectable dosage forms, having thereby the potential for further evaluation for injection purposes. For an excipient to obtain acceptable regulatory status for intravenous injection, it needs to be, among other things, non-hemolytic, non-toxic, non-immunogenic, non-carcinogenic, and non-sensitizing. Interestingly, despite the FDA approval, there have been reports of severe immunogenic and hypersensitivity reactions following the intravenous injection of certain excipients. Examples include PSs, PEGs, and PEG-containing surfactants, as well as albumin–IFN (interferon) aggregates.

A further issue is, of course, the stability of the excipients. Similar to PSs, excipients comprising an ester bond, such as sugar and PEG esters, suffer from susceptibility to chemical and enzymatic-mediated hydrolysis. Moreover, many of the debated surfactants contain a POE (polyethylene oxide) group, which can be prone to auto-oxidation. Poloxamers^®^ degrade at higher temperatures, initiating from the PPO (polypropylene oxide) center block and continuing by the breakage of the POE moiety. Carbohydrate excipients might also be sensitive to enzymatic degradation through host cell polysaccharidase traces. To what extent this is the case for each carbohydrate subclass, and how much it can exacerbate the quality of the formulation, however, remains to be investigated. Even in the case of potential instability of a given excipient, the rate of degradation and the minimum concentration required to protect the integrity of the protein are relevant factors that need to be considered. Despite predisposition to degradation, an excipient might still serve as a suitable PS alternative if a slower degradation rate is observed, or in case the surfactant can maintain protein stability even at lower concentrations. On a final note, on the subject, the availability or development of suitable analytical methods is a prerequisite for the investigation of the excipient stability and the detection and quantification of the potential degradation products.

A last issue, which is perhaps less considered, is the compatibility of the excipient not only with other formulation components, but also with the primary packaging. For instance, due to interaction with silicone oil, P188 has been shown to generate large PDMS (polydimethylsiloxane) particles over long-term storage in vials with siliconized stoppers [241]. Such findings, need however, to be further investigated. Further, PSs and dodecyl-β-d-maltoside have been shown to increase the glide force of the prefilled syringes [242]. On the other hand, little is known about the stability of prefilled syringes or regular glass vials with stoppers of different chemical compositions when in long-term contact with surfactant containing formulations. These findings highlight the importance of an in-depth investigation of not only the stability and degradation of the surfactant, but also the potential interactions with the primary packaging.

The current review sought to introduce potential PS alternatives for protein stabilization in parenteral biotherapeutic formulations. The fact that many of the debated excipients are yet to be profoundly investigated raises hope that future research can uncover the efficiency of some excipients to overcome the limitations and drawbacks of PSs in the formulation of protein biotherapeutics. While some of the introduced excipients might turn up to be suitable PS alternatives on their own, others can serve as a backbone for chemical modifications, through which the shortcomings of the parent molecule can be eliminated, or at least mitigated. An example is the development of the surfactant classes SEs (sucrose esters) and sugar monoesters. In both cases, chemical modification has enabled a significant improvement of the surface-active properties of the parent molecule, thereby improving the ability of the excipient to protect the protein integrity. It should also be noted that at the end of the day, one excipient on its own might not hold the key to the optimal stabilization of protein biopharmaceutical formulations. Sometimes, the use of a mixture of different excipients can provide a viable option [376]. Not only can this enable a more efficient stabilization of the protein formulation against different sources of stress, but other excipients can also ensure the continued solubilization of the formed protein particles and the potential degradation products of an excipient that has, for some reason, suffered from instability. Using excipient mixtures also reduces the required concentration of each component, which in turn decreases the risk of the potential side effects that can arise from each inactive individually.

## Figures and Tables

**Table 1:** Type and content of the fatty acids present in PS20 and PS80 raw material [98,99].

Fatty Acid	Polysorbate 20 [%]	Polysorbate 80 [%]
Caproic acid	max. 1.0	-
Caprylic acid	max. 1.0	-
Capric acid	max. 10.0	-
Lauric acid	40.0–60.0	-
Myristic acid	14.0–25.0	max. 5.0
Palmitic acid	7.0–15.0	max. 16.0
Palmitoleic acid	-	max. 8.0
Stearic acid	max. 7.0	max. 6.0
Oleic acid	max. 11.0	Min. 58.0
Linoleic acid	max. 3.0	max. 18.0
Linolenic acid	-	max. 4.0

**Table 2:** Summary of the available information on the potential alternatives to PSs for the stabilization of protein biotherapeutic formulations.

Excipient		Protein Stabilization Efficiency	Physicochemical Stability	Regulatory Status and Safety	Comparative Studies to PS
	Subcategory				
Surfactants	Sucrose fatty acid esters	remains to be investigated. As in the case of other surfactants, protection of the protein integrity through direct interaction as well as competitive interface adsorption is expected.	are susceptible to chemical and enzymatic cleavage of ester bond and enzymatic degradation of the sugar moiety	- GRAS - FDA-approved for food and cosmetic use - sucrose stearate and sucrose palmitate: FDA-approved for oral and topical formulations - sucrose monopalmitate (0.5 g/kg): resulted in hemolytic reactions [183,184]	Not available
	Sugar monoesters	remains to be investigated. As in the case of other surfactants, protection of the protein integrity through direct interaction, as well as competitive interface adsorption being expected.	are susceptible to chemical and enzymatic cleavage of ester bond and enzymatic degradation of the sugar moiety	nontoxic and biodegradable emulsifiers for use in the food industry [190]	Not available
	Polyethylene glycol (PEG) stearates and PEG fatty esters	polyoxyl 15 hydroxy stearate and polyoxyl 35 castor oil (Kolliphor^®^ HS 15 and Kolliphor^®^ EL): protect amylase and bovine serum albumin (BSA) from chemical and mechanical stresses [204]. As in the case of other surfactants, protection of the protein integrity through direct interaction therewith as well as competitive interface adsorption is expected.	are susceptible to chemical and enzymatic cleavage of ester bond and oxidation of POE [203]	- PEG 2 stearate: FDA-approved for topical application - PEG 8 and 100 stearates: FDA-approved for oral and topical application - PEG 40 stearate: FDA-approved for ophthalmic, oral, and topical applications - polyoxyl 15 hydroxy stearate and polyoxyl 35 castor oil: FDA approval for oral, parenteral, and ophthalmic application - potential PEG-mediated immunogenicity and hypersensitivity	- polyoxyl 15 hydroxy stearate: superior toxicity profile to PS80 [209] - polyoxyl 15 hydroxy stearate and polyoxyl 35 castor oil: comparable efficiency in protecting BSA against mechanical stress for 7 days [204] - polyoxyl 35 castor oil: superior efficiency to stabilize amylase in the presence of H_2_O_2_ over a period of two months [204]
	Block polyethylene-propylene glycols (Poloxamers^®^)	protect the protein through direct interaction as well as competitive interface adsorption	are susceptible to thermal oxidation of PPO block in solid and liquid states	- P188: FDA-approved for intravenous formulations, part of commercial biotherapeutic formulations such as Gazyva^®^, Orencia^®^, Norditropin^®^, and Hemlibra^®^ - potential PEG-mediated immunogenicity and hypersensitivity	P188: Comparable efficiency with PSs in stabilizing mAb formulations, production of PDMS during long-term storage due to interactions with the silicone oil in the stopper in case of P188 [241], increase of glide force in prefilled syringes by PS, but not P188
	Polyoxyethylene fatty ethers (Brij^®^)	protect the protein through direct interaction as well as competitive interface adsorption [247,248]	might be susceptible to metal- and photo-induced oxidation [78,251]	- FDA-approved for topical administration - Brij^®^ 58: no side effects when administrated in a concentration of 20 mg/mL in animal toxicological studies [249]	- Brij^®^ 92: comparable efficiency to PSs in stabilizing rhGH [247] - Brij^®^ 35: performed inferior to PSs in improving the mechanical and thermal stability of mAb, but superior in protecting the protein against photo-oxidation [250] - Brij^®^ 58: superior inherent stability of the surfactant in protein formulations when compared to PS20 and PS80 [249]
	Non-ester sugar-based surfactants	n-dodecyl-β-D-maltoside: serves as cryoprotectant for D-alanine Peptide T-amide [253], reduces the aggregation and immunogenicity of human IFN- β [254], prevents insulin self-association under mechanical stress in liquid state [253]	susceptible to enzymatic degradation of the sugar moiety	- n-dodecyl-β-D-maltoside: classified by EPA as non-mutagenic, non-toxic, and non-irritating [252] - biodegradable and biocompatible	Not available
	N-alkyl amino acid polyether amides	N-myristoyl phenylalanine Jeffamine M1000 diamide (FM1000): protects proteins from thermal and mechanical stresses [255,256]	FM1000: potential susceptibility of the amide bond to hydrolysis, through to a significantly lower extent compared with ester bond in ester surfactants	Not available	FM1000: 3-fold more reduction of thermal-induced abatacept aggregation compared to PS20 and PS80 [255], inferior, and superior IgG protection from agitation-mediated stress compared to PS80 and PS20, respectively [256], protein stabilization from agitation-mediated stress in IV bags was superior to both PS20 and PS80 [256]
Carbohydrates	Disaccharides and sugar alcohols	- protect the proteins against thermal and to some extent mechanical stresses in solid and liquid state, act mostly by altering protein–solvent interactions, also serve as molecular crowders [259,272] - mostly improve the thermal stability of the protein - act as cryoprotectants - are associated with inadequate protein protection necessitating the co-application of surfactants - chemical modification, e.g., esterification with fatty acids, etc. improves the protein stabilization efficiency	are susceptible to enzymatic degradation	FDA-approved, GRAS, established excipients in the formulation of intravenously injected formulations	are often used in combination with PSs to confer proteins adequate stability
	Cyclo- dextrins	protect protein either through direct interaction (in case of certain proteins with substantial solvent exposure to hydrophobic amino-acid residues [311]) or competitive interfacial adsorption (in case of CD with higher surface-active properties [307]), dominant mechanism also depends on protein concentration	are potentially susceptible to enzymatic degradation	- 2-hydroxypropyl-γ-cyclodextrin; FDA-approved for ophthalmic and topical administration - α-cyclodextrin: FDA-approved for intracavitary administration - β-cyclodextrin and sulfobutylether-β-cyclodextrin: FDA-approved for oral, topical, intramuscular, subcutaneous, and intravenous administration (GRAS)	hydroxypropyl-β-cyclodextrin: reduced the interface-induced precipitation of porcine growth hormone through a mechanism similar to PS20 [307], protects PS20 from enzymatic degradation [324] thereby combination of the two might be beneficial for overcoming the drawbacks associated with PS degradation
	Hydroxypropyl methyl cellulose (HPMC)	efficiently stabilizes mAb formulations such as cetuximab and abatacept [330]	is potentially susceptible to enzymatic degradation	FDA-approved, GRAS excipient for oral, buccal, vaginal, and nasal administration	stabilization of cetuximab comparable with PS80 [330]
	Dextran	stabilize the proteins through a crowding effect [332], reduce the surface adsorption of the proteins in the liquid state [53], maintain protein structural integrity during freezing, drying, and storage in the solid state [333]	potential susceptibility to enzymatic degradation	used in several injectable formulations	Not available
Amino acid-based stabilizers	Amino acids	- certain amino acids promote protein solubilization and refolding mostly by altering protein–solvent interactions, also serve as molecular crowders, stabilizing effect of amino acids is protein dependent - arginine protects protein in solution, and acts as a cryoprotectant [255,256]	are not prone to degradation under formulation and storage conditions	- GRAS - FDA-approved for injectable (intramuscular, intravenous), and oral formulations	Not available
	Polyamines	protect proteins against thermal aggregation and denaturation	Not available	- oral acute toxicity dose of 2000, 600, and 600 mg/kg body weight for putrescine, spermidine, and spermine, respectively [363] - no-observed adverse effect level (NOAEL) following oral administration was 2000 ppm (180 mg/kg body weight/day) for putrescine, 1000 ppm (83 mg/ kg body weight/day) for spermidine and 200 ppm (19 mg/kg body weight/day) for spermine [363] oral administration of spermidine in human and animal models has been well-tolerated [362]	Not available
	Albumin	protects protein through direct interaction as well as competitive interface adsorption [365,366,368]	is potentially prone to forming aggregates with itself or other proteins	- is marketed as an injectable product on its own, and is approved as an excipient for injectable formulations - hypersensitivity and immunological reactions to albumin-containing IFN formulations have been reported [23] - concerns regarding blood-transmitted diseases in case of human and bovine serum albumin	Not available
Synthetic amphiphilic polymers	Polyether polyols (PEG) and Polypropylene glycols (PPG)	- PEGs: increase protein stability through covalent binding, protection of the free protein from thermal and lyophilization stresses [373,374] - PPGs: protect cetuximab from agitation-induced stress, potentially through direct interaction with the protein [330]	are potentially susceptible to oxidation	- PEGs: FDA-approved for intravenous administration, immunogenicity, and hypersensitivity to formulations containing PEGs and their derivatives have been reported [226] - PPGs: FDA-approved for oral and topical formulations, cross-reactivity of anti-PEG antibodies with PPGs is possible [378]	PPGs: comparable efficiency with PS80 in protecting cetuximab from agitation-induced stress [378]
	Polyampholytes	protect proteins from thermal and agitation-induced stress [380]	Not available	Not available	Not available
Ionic liquids		- can protect the proteins against thermal and storage-mediated unfolding [387,390,391,392] - can induce refolding of denatured proteins [388,389] - stabilizing effects depends on the concentration, the length of the alkyl chain and the nature and size of the anion [385]	are generally recognized as highly stable [414]	Toxicity studies have been limited to bacteria, fungi, plants, small animals, and human cell lines [408,409]	Not available

BSA: bovine serum albumin, CD: cyclodextrin, EPA: environmental Protection Agency, FDA: Food and Drug Administration, FM100: N-myristoyl phenylala-nine Jeffamine M1000 diamide, GRAS: generally recognized as safe, HPMC: hydroxypropyl methyl cellulose, IFN-β: interferon beta, IgG: immunoglobulin G, IV: intravenous, mAb: monoclonal antibody, NOAEL: no-observed adverse effect level, P188: poloxamer 188, PEG: polyethyelene glycol, PDMS: protein–polydimethylsiloxane, ppm: parts per million, PSs: polysorbates, POE: polyethylene oxide, PPG: polypropylene glycol, PPO: polypropylene oxide, rhGH: recombinant human growth hormone.

## Data Availability

Not applicable.

## Supplementary Materials

The following supporting information can be downloaded at: https://www.mdpi.com/article/10.3390/pharmaceutics14122575/s1, Table S1: FDA and CBER-approved protein biopharmaceuticals containing PS20 and PS80 as a surfactant indicating the amount of surfactant per mL. Data obtained from https://www.accessdata.fda.gov/scripts/cder/iig/index.cfm (accessed on 10 August 2022).

## References

[1] Vulto A.G., Jaquez O.A. (2017). The Process Defines the Product: What Really Matters in Biosimilar Design and Production?. Rheumatology.

[2] Center for Biologics Evaluation and Research (CBER) (2019). What Are “Biologics” Questions and Answers.

[3] Dimitrov D.S. (2012). Therapeutic Proteins. Methods Mol. Biol..

[4] Kroon D.J., Baldwin-Ferro A., Lalan P. (1992). Identification of Sites of Degradation in a Therapeutic Monoclonal Antibody by Peptide Mapping. Pharm Res..

[5] Tous G.I., Wei Z., Feng J., Bilbulian S., Bowen S., Smith J., Strouse R., McGeehan P., Casas-Finet J., Schenerman M.A. (2005). Characterization of a Novel Modification to Monoclonal Antibodies: Thioether Cross-Link of Heavy and Light Chains. Anal. Chem..

[6] Harris R.J., Kabakoff B., Macchi F.D., Shen F.J., Kwong M., Andya J.D., Shire S.J., Bjork N., Totpal K., Chen A.B. (2001). Identification of Multiple Sources of Charge Heterogeneity in a Recombinant Antibody. J. Chromatogr. B Biomed. Sci. Appl..

[7] Wang W. (1999). Instability, Stabilization, and Formulation of Liquid Protein Pharmaceuticals. Int. J. Pharm..

[8] Cleland J.L., Powell M.F., Shire S.J. (1993). The Development of Stable Protein Formulations: A Close Look at Protein Aggregation, Deamidation, and Oxidation. Crit. Rev. Ther. Drug Carr. Syst..

[9] Xie M., Schowen R.L. (1999). Secondary Structure and Protein Deamidation. J. Pharm. Sci..

[10] Usami A., Ohtsu A., Takahama S., Fujii T. (1996). The Effect of PH, Hydrogen Peroxide and Temperature on the Stability of Human Monoclonal Antibody. J. Pharm. Biomed. Anal..

[11] Lam X.M., Yang J.Y., Cleland J.L. (1997). Antioxidants for Prevention of Methionine Oxidation in Recombinant Monoclonal Antibody HER2. J. Pharm. Sci..

[12] Kennedy D.M., Skillen A.W., Self C.H. (1994). Glycation of Monoclonal Antibodies Impairs Their Ability to Bind Antigen. Clin. Exp. Immunol..

[13] Cacia J., Keck R., Presta L.G., Frenz J. (1996). Isomerization of an Aspartic Acid Residue in the Complementarity-Determining Regions of a Recombinant Antibody to Human IgE: Identification and Effect on Binding Affinity. Biochemistry.

[14] Zheng J.Y., Janis L.J. (2006). Influence of PH, Buffer Species, and Storage Temperature on Physicochemical Stability of a Humanized Monoclonal Antibody LA298. Int. J. Pharm..

[15] Wang W., Singh S., Zeng D.L., King K., Nema S. (2007). Antibody Structure, Instability, and Formulation. J. Pharm. Sci..

[16] Liu D., Ren D., Huang H., Dankberg J., Rosenfeld R., Cocco M.J., Li L., Brems D.N., Remmele R.L. (2008). Structure and Stability Changes of Human IgG1 Fc as a Consequence of Methionine Oxidation. Biochemistry.

[17] Kozlowski S., Swann P. (2006). Current and Future Issues in the Manufacturing and Development of Monoclonal Antibodies. Adv. Drug Deliv. Rev..

[18] Geiger T., Clarke S. (1987). Deamidation, Isomerization, and Racemization at Asparaginyl and Aspartyl Residues in Peptides. Succinimide-Linked Reactions That Contribute to Protein Degradation. J. Biol. Chem..

[19] Chen B., Bautista R., Yu K., Zapata G.A., Mulkerrin M.G., Chamow S.M. (2003). Influence of Histidine on the Stability and Physical Properties of a Fully Human Antibody in Aqueous and Solid Forms. Pharm. Res..

[20] Li S.-Q., Bomser J.A., Zhang Q.H. (2005). Effects of Pulsed Electric Fields and Heat Treatment on Stability and Secondary Structure of Bovine Immunoglobulin G. J. Agric. Food Chem..

[21] Hermeling S., Crommelin D.J.A., Huub S., Huub W. (2004). Structure-Immunogenicity Relationships of Therapeutic Proteins. Pharm. Res..

[22] Shire S.J., Shahrokh Z., Liu J. (2004). Challenges in the Development of High Protein Concentration Formulations. J. Pharm. Sci..

[23] Braun A., Kwee L., Labow M.A., Alsenz J. (1997). Protein Aggregates Seem to Play a Key Role among the Parameters Influencing the Antigenicity of Interferon Alpha (IFN-Alpha) in Normal and Transgenic Mice. Pharm. Res..

[24] Sukumar M., Doyle B.L., Combs J.L., Pekar A.H. (2004). Opalescent Appearance of an IgG1 Antibody at High Concentrations and Its Relationship to Noncovalent Association. Pharm. Res..

[25] Andya J.D., Hsu C.C., Shire S.J. (2003). Mechanisms of Aggregate Formation and Carbohydrate Excipient Stabilization of Lyophilized Humanized Monoclonal Antibody Formulations. AAPS PharmSci..

[26] Doran P.M. (2006). Loss of Secreted Antibody from Transgenic Plant Tissue Cultures Due to Surface Adsorption. J. Biotechnol..

[27] Wang T., Kumru O.S., Yi L., Wang Y.J., Zhang J., Kim J.H., Joshi S.B., Middaugh C.R., Volkin D.B. (2013). Effect of Ionic Strength and PH on the Physical and Chemical Stability of a Monoclonal Antibody Antigen-Binding Fragment. J. Pharm. Sci..

[28] Wang W. (2005). Protein Aggregation and Its Inhibition in Biopharmaceutics. Int. J. Pharm..

[29] Garidel P., Blume A., Wagner M. (2015). Prediction of Colloidal Stability of High Concentration Protein Formulations. Pharm. Dev. Technol..

[30] Garidel P., Pevestorf B., Bahrenburg S. (2015). Stability of Buffer-Free Freeze-Dried Formulations: A Feasibility Study of a Monoclonal Antibody at High Protein Concentrations. Eur. J. Pharm. Biopharm..

[31] Kalonia C., Toprani V., Toth R., Wahome N., Gabel I., Middaugh C.R., Volkin D.B. (2016). Effects of Protein Conformation, Apparent Solubility, and Protein-Protein Interactions on the Rates and Mechanisms of Aggregation for an IgG1 Monoclonal Antibody. J. Phys. Chem. B.

[32] Saluja A., Badkar A.V., Zeng D.L., Kalonia D.S. (2007). Ultrasonic Rheology of a Monoclonal Antibody (IgG2) Solution: Implications for Physical Stability of Proteins in High Concentration Formulations. J. Pharm. Sci..

[33] Chi E.E., Krishnan S., Kendrick B.S., Change B.S., Carpenter J.F., Chi T.w. (2003). Roles of Conformational Stability and Colloidal Stability in the Aggregation of Recombinant Human Granulocyte Colony-Stimulating Factor. Protein Sci. A Publ. Protein Soc..

[34] Saito S., Hasegawa J., Kobayashi N., Tomitsuka T., Uchiyama S., Fukui K. (2013). Effects of Ionic Strength and Sugars on the Aggregation Propensity of Monoclonal Antibodies: Influence of Colloidal and Conformational Stabilities. Pharm. Res..

[35] Reiche K., Harti J., Blume A., Garidel P. (2017). Liquid-Liquid Phase Separation of a Monoclonal Antibody at Low Ionic Strength: Influence of Anion Charge and Concentration. Biophys. Chem..

[36] Jiskoot W., Randolph T.W., Volkin D.B., Middaugh C.R., Schöneich C., Winter G., Friess W., Crommelin D.J.A., Carpenter J.F. (2012). Protein Instability and Immunogenicity: Roadblocks to Clinical Application of Injectable Protein Delivery Systems for Sustained Release. J. Pharm. Sci..

[37] Schiefelbein L.K.J. (2011). Sugar-Based Surfactants for Pharmaceutical Protein Formulations.

[38] Gidalevitz D., Huang Z., Rice S.A. (1999). Protein Folding at the Air-Water Interface Studied with X-ray Reflectivity. Proc. Natl. Acad. Sci. USA.

[39] Jones L.S., Kaufmann A., Middaugh R. (2005). Silicone Oil Induced Aggregation of Proteins. J. Pharm. Sci..

[40] Liu L., Ammar D.A., Ross L.A., Mandava N., Kahook M.Y., Carpenter J.F. (2011). Silicone Oil Microdroplets and Protein Aggregates in Repackaged Bevacizumab and Ranibizumab: Effects of Long-Term Storage and Product Mishandling. Investig. Ophthalmol. Vis. Sci..

[41] Li J., Pinnamaneni S., Quan Y., Jaiswal A., Andersson F.I., Zhang X. (2012). Mechanistic Understanding of Protein-Silicone Oil Interactions. Pharm. Res..

[42] Krayukhina E., Tsumoto K., Uchiyama S., Fukui K. (2015). Effects of Syringe Material and Silicone Oil Lubrication on the Stability of Pharmaceutical Proteins. J. Pharm. Sci..

[43] Gerhardt A., McUmber A.C., Nguyen B.H., Lewus R., Schwartz D.K., Carpenter J.F., Randolph T.W. (2015). Surfactant Effects on Particle Generation in Antibody Formulations in Pre-Filled Syringes. J. Pharm. Sci..

[44] Jordan S., Katz J. (2020). Alternative Surfactants for Biologics Stabilization. https://www.pharma.dupont.com/content/dam/dupont/amer/us/en/nutrition-health/general/pharmaceuticals/documents/Biologics%20stabilization%20white%20paper.pdf.

[45] van Beers M.M.C., Sauerborn M., Gilli F., Brinks V., Schellekens H., Jiskoot W. (2011). Oxidized and Aggregated Recombinant Human Interferon Beta Is Immunogenic in Human Interferon Beta Transgenic Mice. Pharm. Res..

[46] Bessa J., Boeckle S., Beck H., Buckel T., Schlicht S., Ebeling M., Kiialainen A., Koulov A., Boll B., Weiser T. (2015). The Immunogenicity of Antibody Aggregates in a Novel Transgenic Mouse Model. Pharm. Res..

[47] Palazzi L., Leri M., Cesaro S., Stefani M., Bucciantini M., Polverino de Laureto P. (2020). Insight into the Molecular Mechanism Underlying the Inhibition of α-Synuclein Aggregation by Hydroxytyrosol. Biochem. Pharmacol..

[48] Moussa E.M., Panchal J.P., Moorthy B.S., Blum J.S., Joubert M.K., Narhi L.O., Topp E.M. (2016). Immunogenicity of Therapeutic Protein Aggregates. J. Pharm. Sci..

[49] Yadav S., Laue T.M., Kalonia D.S., Singh S.N., Shire S.J. (2012). The Influence of Charge Distribution on Self-Association and Viscosity Behavior of Monoclonal Antibody Solutions. Mol. Pharm..

[50] Yadav S., Liu J., Shire S.J., Kalonia D.S. (2010). Specific Interactions in High Concentration Antibody Solutions Resulting in High Viscosity. J. Pharm. Sci..

[51] Rao V.A., Kim J.J., Patel D.S., Rains K., Estoll C.R. (2020). A Comprehensive Scientific Survey of Excipients Used in Currently Marketed, Therapeutic Biological Drug Products. Pharm. Res..

[52] Rayaprolu B.M., Strawser J.J., Anyarambhatla G. (2018). Excipients in Parenteral Formulations: Selection Considerations and Effective Utilization with Small Molecules and Biologics. Drug Dev. Ind. Pharm..

[53] Jorgensen L., Hostrup S., Moeller E.H., Grohganz H. (2009). Recent Trends in Stabilising Peptides and Proteins in Pharmaceutical Formulation-Considerations in the Choice of Excipients. Expert Opin. Drug Deliv..

[54] Akers M.J. (2002). Excipient-Drug Interactions in Parenteral Formulations. J. Pharm. Sci..

[55] Wang S., Wu G., Zhang X., Tian Z., Zhang N., Hu T., Dai W., Qian F. (2017). Stabilizing Two IgG1 Monoclonal Antibodies by Surfactants: Balance between Aggregation Prevention and Structure Perturbation. Eur. J. Pharm. Biopharm..

[56] Garidel P., Blech M., Buske J., Blume A. (2021). Surface Tension and Self-Association Properties of Aqueous Polysorbate 20 HP and 80 HP Solutions: Insights into Protein Stabilisation Mechanisms. J. Pharm. Innov..

[57] Garidel P., Hoffmann C., Blume A. (2009). A Thermodynamic Analysis of the Binding Interaction between Polysorbate 20 and 80 with Human Serum Albumins and Immunoglobulins: A Contribution to Understand Colloidal Protein Stabilisation. Biophys. Chem..

[58] Otzen D. (2011). Protein-Surfactant Interactions: A Tale of Many States. Biochim. Biophys. Acta.

[59] Kaplon H., Reichert J.M. (2019). Antibodies to Watch in 2019. MAbs.

[60] Kaplon H., Muralidharan M., Schneider Z., Reichert J.M. (2020). Antibodies to Watch in 2020. MAbs.

[61] Kaplon H., Reichert J.M. (2021). Antibodies to Watch in 2021. MAbs.

[62] Kreilgaard L., Jones L.S., Randolph T.W., Frokjaer S., Flink J.M., Manning M.C., Carpenter J.F. (1998). Effect of Tween 20 on Freeze-Thawing- and Agitation-Induced Aggregation of Recombinant Human Factor XIII. J. Pharm. Sci..

[63] Hillgren A., Lindgren J., Aldén M. (2002). Protection Mechanism of Tween 80 during Freeze–Thawing of a Model Protein, LDH. Int. J. Pharm..

[64] Bam N.B., Cleland J.L., Yang J., Manning M.C., Carpenter J.F., Kelley R.F., Randolph T.W. (1998). Tween Protects Recombinant Human Growth Hormone against Agitation-Induced Damage via Hydrophobic Interactions. J. Pharm. Sci..

[65] Zhang L., Yadav S., Demeule B., Wang Y.J., Mozziconacci O., Schöneich C. (2017). Degradation Mechanisms of Polysorbate 20 Differentiated by 18O-Labeling and Mass Spectrometry. Pharm. Res..

[66] Edelmann F., Ravuri K. Instability of Polysorbates in Protein Biopharmaceutics. Proceedings of the 10th Global Drug Delivery and Formulation Summit.

[67] Santana H., González Y., Campana P.T., Noda J., Amarantes O., Itri R., Beldarraín A., Páez R. (2013). Screening for Stability and Compatibility Conditions of Recombinant Human Epidermal Growth Factor for Parenteral Formulation: Effect of PH, Buffers, and Excipients. Int. J. Pharm..

[68] Ha E., Wang W., Wang Y.J. (2002). Peroxide Formation in Polysorbate 80 and Protein Stability. J. Pharm. Sci..

[69] Reyes N., Ruiz L., Aroche K., Gerónimo H., Brito O., Hardy E. (2005). Stability of Ala 125 Recombinant Human Interleukin-2 in Solution. J. Pharm. Pharmacol..

[70] Kerwin B.A. (2008). Polysorbates 20 and 80 Used in the Formulation of Protein Biotherapeutics: Structure and Degradation Pathways. J. Pharm. Sci..

[71] Kishore R.S.K., Kiese S., Fischer S., Pappenberger A., Grauschopf U., Mahler H.-C. (2011). The Degradation of Polysorbates 20 and 80 and Its Potential Impact on the Stability of Biotherapeutics. Pharm. Res..

[72] Brange J., Langkjaer L., Havelund S., Vølund A. (1992). Chemical Stability of Insulin. 1. Hydrolytic Degradation during Storage of Pharmaceutical Preparations. Pharm. Res..

[73] Donbrow M., Azaz E., Pillersdorf A. (1978). Autoxidation of Polysorbates. J. Pharm. Sci..

[74] Labrenz R. (2014). Ester Hydrolysis of Polysorbate 80 in MAb Drug Product: Evidence in Support of the Hypothesized Risk after the Observation of Visible Particulate in MAb Formulations. J. Pharm. Sci..

[75] Lipiäinen T., Peltoniemi M., Sarkhel S., Yrjönen T., Vuorela H., Urtti A., Juppo A. (2015). Formulation and Stability of Cytokine Therapeutics. J. Pharm. Sci..

[76] Jaeger J., Sorensen K., Wolff S.P. (1994). Peroxide Accumulation in Detergents. J. Biochem. Biophys. Methods.

[77] Li S., Schöneich C., Borchardt R.T. (1995). Chemical Instability of Protein Pharmaceuticals: Mechanisms of Oxidation and Strategies for Stabilization. Biotechnol. Bioeng..

[78] Currie F., Andersson M., Holmber K. (2004). Oxidation of Self-Organized Nonionic Surfactants. Langmuir ACS J. Surf. Colloids.

[79] Wuchner K., Yi L., Chery C., Nikels F., Junge F., Crotts G., Rinaldi G., Starkey J.A., Bechtold-Peters K., Shuman M. (2022). Industry Perspective on the Use and Characterization of Polysorbates for Biopharmaceutical Products Part 1: Survey Report on Current State and Common Practices for Handling and Control of Polysorbates. J. Pharm. Sci..

[80] Wuchner K., Yi L., Chery C., Nikels F., Junge F., Crotts G., Rinaldi G., Starkey J.A., Bechtold-Peters K., Shuman M. (2022). Industry Perspective on the Use and Characterization of Polysorbates for Biopharmaceutical Products Part 2: Survey Report on Control Strategy Preparing for the Future. J. Pharm. Sci..

[81] Ionova Y., Wilson L. (2020). Biologic Excipients: Importance of Clinical Awareness of Inactive Ingredients. PLoS ONE.

[82] Nema S., Brendel R.J. (2011). Excipients and Their Role in Approved Injectable Products: Current Usage and Future Directions. PDA J. Pharm. Sci. Technol..

[83] Liu L., Qi W., Schwartz D.K., Randolph T.W., Carpenter J.F. (2013). The Effects of Excipients on Protein Aggregation during Agitation: An Interfacial Shear Rheology Study. J. Pharm. Sci..

[84] Carpenter J.F., Chang B.S., Garzon-Rodriguez W., Randolph T.W. (2002). Rational Design of Stable Lyophilized Protein Formulations: Theory and Practice. Pharm. Biotechnol..

[85] Jayasundera M., Adhikari B., Adhikari R., Aldred P. (2011). The Effect of Protein Types and Low Molecular Weight Surfactants on Spray Drying of Sugar-Rich Foods. Food Hydrocoll..

[86] Gerhardt A., Bonam K., Bee J.S., Carpenter J.F., Randolph T.W. (2013). Ionic Strength Affects Tertiary Structure and Aggregation Propensity of a Monoclonal Antibody Adsorbed to Silicone Oil-Water Interfaces. J. Pharm. Sci..

[87] Lee H.J., McAuley A., Schilke K.F., McGuire J. (2011). Molecular Origins of Surfactant-Mediated Stabilization of Protein Drugs. Adv. Drug Deliv. Rev..

[88] Bam N.B., Cleland J.L., Randolph T.W. (1996). Molten Globule Intermediate of Recombinant Human Growth Hormone: Stabilization with Surfactants. Biotechnol. Prog..

[89] Lee L.L., Lee J.C. (1987). Thermal Stability of Proteins in the Presence of Poly(Ethylene Glycols). Biochemistry.

[90] Arakawa T., Timasheff S.N. (1985). Mechanism of Poly(Ethylene Glycol) Interaction with Proteins. Biochemistry.

[91] Dixit N., Maloney K.M., Kalonia D.S. (2013). Protein-Silicone Oil Interactions: Comparative Effect of Nonionic Surfactants on the Interfacial Behavior of a Fusion Protein. Pharm. Res..

[92] Ayorinde F.O., Gelain S.V., Johnson J.H., Wan L.W. (2000). Analysis of Some Commercial Polysorbate Formulations Using Matrix-Assisted Laser Desorption/Ionization Time-of-Flight Mass Spectrometry. Rapid Commun. Mass Spectrom..

[93] Frison-Norrie S., Sporns P. (2001). Investigating the Molecular Heterogeneity of Polysorbate Emulsifiers by MALDI-TOF MS. J. Agric. Food Chem..

[94] Dwivedi M., Blech M., Presser I., Garidel P. (2018). Polysorbate Degradation in Biotherapeutic Formulations: Identification and Discussion of Current Root Causes. Int. J. Pharm..

[95] Hewitt D., Alvarez M., Robinson K., Ji J., Wang Y.J., Kao Y.-H., Zhang T. (2011). Mixed-Mode and Reversed-Phase Liquid Chromatography–Tandem Mass Spectrometry Methodologies to Study Composition and Base Hydrolysis of Polysorbate 20 and 80. J. Chromatogr. A.

[96] Tomlinson A., Zarraga I.E., Demeule B. (2020). Characterization of Polysorbate Ester Fractions and Implications in Protein Drug Product Stability. Mol. Pharm..

[97] Pan J., Ji Y., Du Z., Zhang J. (2016). Rapid Characterization of Commercial Polysorbate 80 by Ultra-High Performance Supercritical Fluid Chromatography Combined with Quadrupole Time-of-Flight Mass Spectrometry. J. Chromatogr. A.

[98] European Pharmacopoeia Commission (2017). European Pharmacopoeia PS80. European Pharmacopoeia.

[99] European Pharmacopoeia Commission (2017). European Pharmacopoeia PS20. European Pharmacopoeia.

[100] Li Y., Hewitt D., Lentz Y.K., Ji J.A., Zhang T.Y., Zhang K. (2014). Characterization and Stability Study of Polysorbate 20 in Therapeutic Monoclonal Antibody Formulation by Multidimensional Ultrahigh-Performance Liquid Chromatography-Charged Aerosol Detection-Mass Spectrometry. Anal. Chem..

[101] Sun H., Yang R., Wang J., Yang X., Tu J., Xie L., Li C., Lao Q., Sun C. (2017). Component-Based Biocompatibility and Safety Evaluation of Polysorbate 80. RSC Adv..

[102] Ravuri K.S.K., Warne N.W., Mahler H.-C. (2018). Polysorbate Degradation and Quality. Challenges in Protein Product Development.

[103] Kishore R.S.K., Pappenberger A., Dauphin I.B., Ross A., Buergi B., Staempfli A., Mahler H.-C. (2011). Degradation of Polysorbates 20 and 80: Studies on Thermal Autoxidation and Hydrolysis. J. Pharm. Sci..

[104] Khossravi M., Kao Y.-H., Mrsny R.J., Sweeney T.D. (2002). Analysis Methods of Polysorbate 20: A New Method to Assess the Stability of Polysorbate 20 and Established Methods That May Overlook Degraded Polysorbate 20. Pharm. Res..

[105] Bates T.R., Nightingale C.H., Dixon E. (1973). Kinetics of Hydrolysis of Polyoxyethylene (20) Sorbitan Fatty Acid Ester Surfactants. J. Pharm. Pharmacol..

[106] Donbrow M., Hamburger R., Azaz E., Pillersdorf A. (1978). Development of Acidity in Non-Ionic Surfactants: Formic and Acetic Acid. Analyst.

[107] Donbrow M., Hamburger R., Azaz E. (1975). Surface Tension and Cloud Point Changes of Polyoxyethylenic Non-Ionic Surfactants during Autoxidation. J. Pharm. Pharmacol..

[108] Carey F.A., Sundberg R.J. (2002). Advanced Organic Chemistry Part A: Structure and Mechanism. Advanced Organic Chemistry.

[109] Dixit N., Salamat-Miller N., Salinas P.A., Taylor K.D., Basu S.K. (2016). Residual Host Cell Protein Promotes Polysorbate 20 Degradation in a Sulfatase Drug Product Leading to Free Fatty Acid Particles. J. Pharm. Sci..

[110] Roy I., Patel A., Kumar V., Nanda T., Assenberg R., Wuchner K., Amin K. (2021). Polysorbate Degradation and Particle Formation in a High Concentration MAb: Formulation Strategies to Minimize Effect of Enzymatic Polysorbate Degradation. J. Pharm. Sci..

[111] Hall T., Sandefur S.L., Frye C.C., Tuley T.L., Huang L. (2016). Polysorbates 20 and 80 Degradation by Group XV Lysosomal Phospholipase A2 Isomer X1 in Monoclonal Antibody Formulations. J. Pharm. Sci..

[112] McShan A.C., Kei P., Ji J.A., Kim D.C., Wang Y.J. (2016). Hydrolysis of Polysorbate 20 and 80 by a Range of Carboxylester Hydrolases. PDA J. Pharm. Sci. Technol..

[113] Siska C.C., Pierini C.J., Lau H.R., Latypov R.F., Fesinmeyer R.M., Litowski J.R. (2015). Free Fatty Acid Particles in Protein Formulations, Part 2: Contribution of Polysorbate Raw Material. J. Pharm. Sci..

[114] Honemann M.N., Wendler J., Graf T., Bathke A., Bell C.H. (2019). Monitoring Polysorbate Hydrolysis in Biopharmaceuticals Using a QC-Ready Free Fatty Acid Quantification Method. J. Chromatogr. B Anal. Technol. Biomed. Life Sci..

[115] Tomlinson A., Demeule B., Lin B., Yadav S. (2015). Polysorbate 20 Degradation in Biopharmaceutical Formulations: Quantification of Free Fatty Acids, Characterization of Particulates, and Insights into the Degradation Mechanism. Mol. Pharm..

[116] Larson N.R., Wei Y., Prajapati I., Chakraborty A., Peters B., Kalonia C., Hudak S., Choudhary S., Esfandiary R., Dhar P. (2020). Comparison of Polysorbate 80 Hydrolysis and Oxidation on the Aggregation of a Monoclonal Antibody. J. Pharm. Sci..

[117] Chiu J., Valente K.N., Levy N.E., Min L., Lenhoff A.M., Lee K.H. (2017). Knockout of a Difficult-to-Remove CHO Host Cell Protein, Lipoprotein Lipase, for Improved Polysorbate Stability in Monoclonal Antibody Formulations. Biotechnol. Bioeng..

[118] Mihara K., Ito Y., Hatano Y., Komurasaki Y., Sugimura A., Jones M., Liu H., Mai S., Lara-Velasco O., Bai L. (2015). Host Cell Proteins: The Hidden Side of Biosimilarity Assessment. J. Pharm. Sci..

[119] Aboulaich N., Chung W.K., Thompson J.H., Larkin C., Robbins D., Zhu M. (2014). A Novel Approach to Monitor Clearance of Host Cell Proteins Associated with Monoclonal Antibodies. Biotechnol. Prog..

[120] Graf T., Tomlinson A., Yuk I.H., Kufer R., Spensberger B., Falkenstein R., Shen A., Li H., Duan D., Liu W. (2021). Identification and Characterization of Polysorbate-Degrading Enzymes in a Monoclonal Antibody Formulation. J. Pharm. Sci..

[121] Zhang S., Riccardi C., Kamen D., Reilly J., Mattila J., Bak H., Xiao H., Li N. (2022). Identification of the Specific Causes of Polysorbate 20 Degradation in Monoclonal Antibody Formulations Containing Multiple Lipases. Pharm. Res..

[122] Li X., Chandra D., Letarte S., Adam G.C., Welch J., Yang R.-S., Rivera S., Bodea S., Dow A., Chi A. (2021). Profiling Active Enzymes for Polysorbate Degradation in Biotherapeutics by Activity-Based Protein Profiling. Anal. Chem..

[123] Zhang S., Xiao H., Molden R., Qiu H., Li N. (2020). Rapid Polysorbate 80 Degradation by Liver Carboxylesterase in a Monoclonal Antibody Formulated Drug Substance at Early Stage Development. J. Pharm. Sci..

[124] Zhang S., Xiao H., Li N. (2021). Degradation of Polysorbate 20 by Sialate O-Acetylesterase in Monoclonal Antibody Formulations. J. Pharm. Sci..

[125] Li X., Wang F., Li H., Richardson D.D., Roush D. (2022). The Measurement and Control of High-Risk Host Cell Proteins for Polysorbate Degradation in Biologics Formulation. Antib. Ther..

[126] (2020). Putative Phospholipase B-like 2 Is Not Responsible for Polysorbate Degradation in Monoclonal Antibody Drug Products. J. Pharm. Sci..

[127] Glücklich N., Carle S., Buske J., Mäder K., Garidel P. (2021). Assessing the Polysorbate Degradation Fingerprints and Kinetics of Lipases—How the Activity of Polysorbate Degrading Hydrolases Is Influenced by the Assay and Assay Conditions. Eur. J. Pharm. Sci..

[128] Saito H., Tomioka H., Watanabe T., Yoneyama T. (1983). Mycobacteriocins Produced by Rapidly Growing Mycobacteria Are Tween-Hydrolyzing Esterases. J. Bacteriol..

[129] Tomioka H. (1983). Purification and Characterization of the Tween-Hydrolyzing Esterase of Mycobacterium Smegmatis. J. Bacteriol..

[130] Jones M.T., Mahler H.-C., Yadav S., Bindra D., Corvari V., Fesinmeyer R.M., Gupta K., Harmon A.M., Hinds K.D., Koulov A. (2018). Considerations for the Use of Polysorbates in Biopharmaceuticals. Pharm. Res..

[131] Bodin A., Linnerborg M., Nilsson J.L.G., Karlberg A.-T. (2002). Novel Hydroperoxides as Primary Autoxidation Products of a Model Ethoxylated Surfactant. J. Surfact Deterg..

[132] Bäcktorp C., Börje A., Nilsson J.L.G., Karlberg A.-T., Norrby P.-O., Nyman G. (2008). Mechanisms of Air Oxidation of Ethoxylated Surfactants--Computational Estimations of Energies and Reaction Behaviors. Chemistry.

[133] Singh S.R., Zhang J., O’Dell C., Hsieh M.-C., Goldstein J., Liu J., Srivastava A. (2012). Effect of Polysorbate 80 Quality on Photostability of a Monoclonal Antibody. AAPS PharmSciTech..

[134] Yao J., Dokuru D.K., Noestheden M., Park S.S., Kerwin B.A., Jona J., Ostovic D., Reid D.L. (2009). A Quantitative Kinetic Study of Polysorbate Autoxidation: The Role of Unsaturated Fatty Acid Ester Substituents. Pharm. Res..

[135] Kerwin B.A., Remmele R.L. (2007). Protect from Light: Photodegradation and Protein Biologics. J. Pharm. Sci..

[136] Du C., Barnett G., Borwankar A., Lewandowski A., Singh N., Ghose S., Borys M., Li Z.J. (2018). Protection of Therapeutic Antibodies from Visible Light Induced Degradation: Use Safe Light in Manufacturing and Storage. Eur. J. Pharm. Biopharm..

[137] Nejadnik M.R., Randolph T.W., Volkin D.B., Schöneich C., Carpenter J.F., Crommelin D.J.A., Jiskoot W. (2018). Postproduction Handling and Administration of Protein Pharmaceuticals and Potential Instability Issues. J. Pharm. Sci..

[138] Kim H.-H., Lee Y.M., Suh J.-K., Song N.W. (2007). Photodegradation Mechanism and Reaction Kinetics of Recombinant Human Interferon-Alpha2a. Photochem. Photobiol. Sci..

[139] Sreedhara A., Yin J., Joyce M., Lau K., Wecksler A.T., Deperalta G., Yi L., John Wang Y., Kabakoff B., Kishore R.S.K. (2016). Effect of Ambient Light on IgG1 Monoclonal Antibodies during Drug Product Processing and Development. Eur. J. Pharm. Biopharm..

[140] Maity H., O’Dell C., Srivastava A., Goldstein J. (2009). Effects of Arginine on Photostability and Thermal Stability of IgG1 Monoclonal Antibodies. Curr. Pharm. Biotechnol..

[141] Bane J., Mozziconacci O., Yi L., Wang Y.J., Sreedhara A., Schöneich C. (2017). Photo-Oxidation of IgG1 and Model Peptides: Detection and Analysis of Triply Oxidized His and Trp Side Chain Cleavage Products. Pharm. Res..

[142] Pattison D.I., Rahmanto A.S., Davies M.J. (2012). Photo-Oxidation of Proteins. Photochem. Photobiol. Sci..

[143] Roy S., Mason B.D., Schöneich C.S., Carpenter J.F., Boone T.C., Kerwin B.A. (2009). Light-Induced Aggregation of Type I Soluble Tumor Necrosis Factor Receptor. J. Pharm. Sci..

[144] Li Y., Polozova A., Gruia F., Feng J. (2014). Characterization of the Degradation Products of a Color-Changed Monoclonal Antibody: Tryptophan-Derived Chromophores. Anal. Chem..

[145] Mason B.D., Schöneich C., Kerwin B.A. (2012). Effect of PH and Light on Aggregation and Conformation of an IgG1 MAb. Mol. Pharm..

[146] Fradkin A.H., Mozziconacci O., Schöneich C., Carpenter J.F., Randolph T.W. (2014). UV Photodegradation of Murine Growth Hormone: Chemical Analysis and Immunogenicity Consequences. Eur. J. Pharm. Biopharm..

[147] Torosantucci R., Schöneich C., Jiskoot W. (2014). Oxidation of Therapeutic Proteins and Peptides: Structural and Biological Consequences. Pharm. Res..

[148] Pham N.B., Meng W.S. (2020). Protein Aggregation and Immunogenicity of Biotherapeutics. Int. J. Pharm..

[149] Coors E.A., Seybold H., Merk H.F., Mahler V. (2005). Polysorbate 80 in Medical Products and Nonimmunologic Anaphylactoid Reactions. Ann. Allergy Asthma Immunol..

[150] Palacios Castaño M.I., Venturini Díaz M., Lobera Labairu T., González Mahave I., Del Pozo Gil M.D., Blasco Sarramián A. (2016). Anaphylaxis Due to the Excipient Polysorbate 80. J. Investig. Allergol. Clin. Immunol..

[151] Badiu I., Guida G., Heffler E., Rolla G. (2015). Multiple Drug Allergy Due to Hypersensitivity to Polyethylene Glycols of Various Molecular Weights. J. Investig. Allergol. Clin. Immunol..

[152] Stone C.A., Liu Y., Relling M.V., Krantz M.S., Pratt A.L., Abreo A., Hemler J.A., Phillips E.J. (2019). Immediate Hypersensitivity to Polyethylene Glycols and Polysorbates: More Common than We Have Recognized. J. Allergy Clin. Immunol. Pract..

[153] Price K.S., Hamilton R.G. (2007). Anaphylactoid Reactions in Two Patients after Omalizumab Administration after Successful Long-Term Therapy. Allergy Asthma Proc..

[154] Dreyfus D.H., Randolph C.C. (2006). Characterization of an Anaphylactoid Reaction to Omalizumab. Ann. Allergy Asthma Immunol..

[155] Pérez-Pérez L., García-Gavín J., Piñeiro B., Zulaica A. (2011). Biologic-Induced Urticaria Due to Polysorbate 80: Usefulness of Prick Test. Br. J. Dermatol..

[156] Bergmann K.C., Maurer M., Church M.K., Zuberbier T. (2020). Anaphylaxis to Mepolizumab and Omalizumab in a Single Patient: Is Polysorbate the Culprit?. J. Investig. Allergol. Clin. Immunol..

[157] Badiu I., Geuna M., Heffler E., Rolla G. (2012). Hypersensitivity Reaction to Human Papillomavirus Vaccine Due to Polysorbate 80. BMJ Case Rep..

[158] Steele R.H., Limaye S., Cleland B., Chow J., Suranyi M.G. (2005). Hypersensitivity Reactions to the Polysorbate Contained in Recombinant Erythropoietin and Darbepoietin. Nephrology.

[159] Center for Biologics Evaluation and Research (CBER) (2022). Pilot Program for the Review of Innovation and Modernization of Excipients (PRIME).

[160] Bollenbach L., Buske J., Mäder K., Garidel P. (2022). Poloxamer 188 as Surfactant in Biological Formulations—An Alternative for Polysorbate 20/80?. Int. J. Pharm..

[161] Inactive Ingredient Search for Approved Drug Products. https://www.accessdata.fda.gov/scripts/cder/iig/index.cfm.

[162] Csóka G., Marton S., Zelko R., Otomo N., Antal I. (2007). Application of Sucrose Fatty Acid Esters in Transdermal Therapeutic Systems. Eur. J. Pharm. Biopharm..

[163] CFR-Code of Federal Regulations Title 21. https://www.accessdata.fda.gov/scripts/cdrh/cfdocs/cfcfr/cfrsearch.cfm?fr=172.859.

[164] Scott L.N., Bergfeld W.F., Belsito D.V., Hill R.A., Klaassen C.D., Liebler D.C., Marks J.G., Shank R.C., Slaga T.J., Snyder P.W. (2021). Safety Assessment of Saccharide Esters as Used in Cosmetics. Int. J. Toxicol..

[165] Obikili A., Deyme M., Wouessidjewe D., Duchěne D. (1988). Improvement of Aqueous Solubility and Dissolution Kinetics of Canrenone by Solid Dispersion in Sucroester. Drug Dev. Ind. Pharm..

[166] Hahn L., Sucker H. (1989). Solid Surfactant Solutions of Active Ingredients in Sugar Esters. Pharm. Res..

[167] Abd-Elbary A., El-laithy H.M., Tadros M.I. (2008). Sucrose Stearate-Based Proniosome-Derived Niosomes for the Nebulisable Delivery of Cromolyn Sodium. Int. J. Pharm..

[168] Lerk P.C., Sucker H. (1993). Application of Sucrose Laurate in Topical Preparations of Cyclosporin A. Int. J. Pharm..

[169] Okamoto H., Sakai T., Danjo K. (2005). Effect of Sucrose Fatty Acid Esters on Transdermal Permeation of Lidocaine and Ketoprofen. Biol. Pharm. Bull..

[170] Ahsan F., Arnold J.J., Meezan E., Pillion D.J. (2003). Sucrose Cocoate, a Component of Cosmetic Preparations, Enhances Nasal and Ocular Peptide Absorption. Int. J. Pharm..

[171] Thevenin M.A., Grossiord J.L., Poelman M.C. (1996). Sucrose Esters/Cosurfactant Microemulsion Systems for Transdermal Delivery: Assessment of Bicontinuous Structures. Int. J. Pharm..

[172] Fanun M. (2008). Surfactant Chain Length Effect on the Structural Parameters of Nonionic Microemulsions. J. Dispers. Sci. Technol..

[173] Mollee H., de Vrind J., De Vringer T. (2000). Stable Reversed Vesicles in Oil: Characterization Studies and Encapsulation of Model Compounds. J. Pharm. Sci..

[174] Honeywell-Nguyen P.L., Bouwstra J.A. (2003). The in Vitro Transport of Pergolide from Surfactant-Based Elastic Vesicles through Human Skin: A Suggested Mechanism of Action. J. Control. Release.

[175] Klang V., Matsko N., Raupach K., El-Hagin N., Valenta C. (2011). Development of Sucrose Stearate-Based Nanoemulsions and Optimisation through γ-Cyclodextrin. Eur. J. Pharm. Biopharm..

[176] Zheng Y., Zheng M., Ma Z., Xin B., Guo R., Xu X. (2015). Sugar Fatty Acid Esters. Polar Lipids.

[177] Soultani S., Ognier S., Engasser J.-M., Ghoul M. (2003). Comparative Study of Some Surface Active Properties of Fructose Esters and Commercial Sucrose Esters. Colloids Surf. A Physicochem. Eng. Asp..

[178] Younes M., Aggett P., Aguilar F., Crebelli R., Dusemund B., Filipič M., Frutos M.J., Galtier D., Gundert-Remy U., Kuhnle G.G. (2010). Scientific opinion on the safety of sucrose esters of fatty acids prepared from vinyl esters of fatty acids and on the extension of use of sucrose esters of fatty acids in flavourings|EFSA. EFSA J..

[179] Thomson A.B., Hunt R.H., Zorich N.L. (1998). Review Article: Olestra and Its Gastrointestinal Safety. Aliment Pharmacol..

[180] Daniel J.W., Marshall C.J., Jones H.F., Snodin D.J. (1979). The Metabolism of Beef Tallow Sucrose Esters in Rat and Man. Food Cosmet. Toxicol..

[181] Shigeoka T., Izawa O., Kitazawa K., Yamauchi F., Murata T. (1984). Studies on the Metabolic Fate of Sucrose Esters in Rats. Food Chem. Toxicol..

[182] Noker P.E., Lin T.H., Hill D.L., Shigeoka T. (1997). Metabolism of 14C-Labelled Sucrose Esters of Stearic Acid in Rats. Food Chem. Toxicol..

[183] Sucrose Esters of Fatty Acids, and Sucroglycerides (WHO Food Additives Series 5). https://inchem.org/documents/jecfa/jecmono/v05je52.htm.

[184] Drummond C.J., Fong C., Krodkiewksa I., Boyd B., Baker I. (2003). Sugar Fatty Acid Esters. Novel Surfactants: Preparation, Applications and Biodegradability.

[185] Savić S., Tamburić S., Savić M.M. (2010). From Conventional towards New-Natural Surfactants in Drug Delivery Systems Design: Current Status and Perspectives. Expert Opin. Drug Deliv..

[186] Szűts A., Szabó-Révész P. (2012). Sucrose Esters as Natural Surfactants in Drug Delivery Systems—A Mini-Review. Int. J. Pharm..

[187] Gaudin T., Lu H., Fayet G., Berthauld-Drelich A., Rotureau P., Pourceau G., Wadouachi A., Van Hecke E., Nesterenko A., Pezron I. (2019). Impact of the Chemical Structure on Amphiphilic Properties of Sugar-Based Surfactants: A Literature Overview. Adv. Colloid Interface Sci..

[188] Lerk P.C., Sucker H.H., Eicke H.F. (1996). Micellization and Solubilization Behavior of Sucrose Laurate, a New Pharmaceutical Excipient. Pharm. Dev. Technol..

[189] Brown D.L. (2010). Parenteral Formulations Comprising Sugar-Based Esters and Ethers. U.S. Patent.

[190] Weber N., Benning H. (1984). Metabolism of Orally Administered Alkyl Beta-Glycosides in the Mouse. J. Nutr..

[191] Garofalakis G., Murray B.S., Sarney D.B. (2000). Surface Activity and Critical Aggregation Concentration of Pure Sugar Esters with Different Sugar Headgroups. J. Colloid Interface Sci..

[192] The European Agency for the Evaluation of Medicinal Products (2003). Polyethylene Glycol Searates and Polyethylene Glycol 15 Hydroxystearate.

[193] Elder E. (1983). Final Report on the Safety Assessment of PEG-2,-6, -8, -12, -20, -32, -40, -50, -100, and -150 Stearates. J. Am. Coll. Toxicol..

[194] Fruijtier-Pölloth C. (2005). Safety Assessment on Polyethylene Glycols (PEGs) and Their Derivatives as Used in Cosmetic Products. Toxicology.

[195] Bárány E., Lindberg M., Lodén M. (2000). Unexpected Skin Barrier Influence from Nonionic Emulsifiers. Int. J. Pharm..

[196] Johns C.H., Pepper W.P. (1983). 5 Final Report on the Safety Assessment of Propylene Glycol Stearate and Propylene Glycol Stearate Self-Emulsifying. J. Am. Coll. Toxicol..

[197] Johnson W. (2001). Cosmetic Ingredient Review Expert Panel Final Report on the Safety Assessment of PEG-25 Propylene Glycol Stearate, PEG-75 Propylene Glycol Stearate, PEG-120 Propylene Glycol Stearate, PEG-10 Propylene Glycol, PEG-8 Propylene Glycol Cocoate, and PEG-55 Propylene Glycol Oleate. Int. J. Toxicol..

[198] Ali S., Kolter K. (2019). Kolliphor® HS 15-An Enabler for Parenteral and Oral Formulations. Am. Pharm. Rev..

[199] Reintjes T. (2011). Solubility Enhancement with BASF Pharma Polymers-Solubilizer Compendium.

[200] Shubber S., Vllasaliu D., Rauch C., Jordan F., Illum L., Stolnik S. (2015). Mechanism of Mucosal Permeability Enhancement of CriticalSorb® (Solutol® HS15) Investigated in Vitro in Cell Cultures. Pharm. Res..

[201] Pilotaz F., Mercier F., Chibret H. (2011). Preservative-Free Prostaglandin-Based Ophthalmic Solution. Portugal Patent.

[202] Dai W.-G., Dong L.C., Li S., Pollock-Dove C., Chen J., Mansky P., Eichenbaum G. (2007). Parallel Screening Approach to Identify Solubility-Enhancing Formulations for Improved Bioavailability of a Poorly Water-Soluble Compound Using Milligram Quantities of Material. Int. J. Pharm..

[203] Berkó S., Regdon G., Erös I. (2002). Solutol and Cremophor Products as New Additives in Suppository Formulation. Drug Dev. Ind. Pharm..

[204] Zakeri B., Kroll S., Romanski F.S. (2020). Expanding the Toolbox of Surfactants Available for Biologics Formulations with Kolliphor® HS 15 and Kolliphor® ELP.

[205] Ruchatz F., Schuch H. (1988). Physicochemical Properties of Solutol HS 15 and Its Solubilizates. BASF ExAct.

[206] Zhao X., Chen D., Gao P., Ding P., Li K. (2005). Synthesis of Ibuprofen Eugenol Ester and Its Microemulsion Formulation for Parenteral Delivery. Chem. Pharm. Bull..

[207] Lee E.-H., Lee S.-H., Park D.-Y., Ki K.-H., Lee E.-K., Lee D.-H., Noh G.-J. (2008). Physicochemical Properties, Pharmacokinetics, and Pharmacodynamics of a Reformulated Microemulsion Propofol in Rats. Anesthesiology.

[208] Bartels M., Vilsendorf A., Kassahun T., Gerstenbergk B., Engelhart K., Vilsendorf E., Faber S., Biesalski H. (2007). Protective Effect of Antioxidative Vitamins against Lipid Peroxidation in Liver Ischemia and Reperfusion–an Animal Experimental Study. EXCLI J..

[209] Volker B. (2008). Pharmaceutical Technology of BASF Excipients.

[210] BASF (2016). Product Regulations Safety Data Sheet- Kolliphor HS 15.

[211] Borisov O.V., Ji J.A., Wang Y.J. (2015). Oxidative Degradation of Polysorbate Surfactants Studied by Liquid Chromatography–Mass Spectrometry. J. Pharm. Sci..

[212] Hvattum E., Yip W.L., Grace D., Dyrstad K. (2012). Characterization of Polysorbate 80 with Liquid Chromatography Mass Spectrometry and Nuclear Magnetic Resonance Spectroscopy: Specific Determination of Oxidation Products of Thermally Oxidized Polysorbate 80. J. Pharm. Biomed. Anal..

[213] Zhang H., Wang Z., Liu O. (2016). Simultaneous Determination of Kolliphor HS15 and Miglyol 812 in Microemulsion Formulation by Ultra-High Performance Liquid Chromatography Coupled with Nano Quantity Analyte Detector. J. Pharm. Anal..

[214] Coon J.S., Knudson W., Clodfelter K., Lu B., Weinstein R.S. (1991). Solutol HS 15, Nontoxic Polyoxyethylene Esters of 12-Hydroxystearic Acid, Reverses Multidrug Resistance. Cancer Res..

[215] Bhaskar V., Middha A., Tiwari S., Shivakumar S. (2013). Identification and Reduction of Matrix Effects Caused by Solutol Hs15 in Bioanalysis Using Liquid Chromatography/Tandem Mass Spectrometry. J. Anal. Bioanal. Tech..

[216] Bhaskar V.V., Middha A., Srivastava P., Rajagopal S. (2015). Liquid Chromatography/Tandem Mass Spectrometry Method for Quantitative Estimation of Solutol HS15 and Its Applications. J. Pharm. Anal..

[217] Cervantes-Martinez C.V., Emo M., Lebeau B., García-Celma M.-J., Stébé M.-J., Blin J.-L. (2019). Insights of the Kolliphor/Water System for the Design of Mesostructured Silica Materials. Microporous Mesoporous Mater..

[218] BASF (2019). Product Regulations Technical Information Sheet- Kolliphor EL.

[219] European Medicine Agency (1999). Polyolyl Caster Oil, Polyoxyl Hydrogenated Caster Oil.

[220] Irizarry L., McKoy J., Samaras A., Fisher M., Carias E., Raisch D., Calhoun E., Bennett C., Brown J., Lurie R. (2009). Cremophor EL-Containing Paclitaxel-Induced Anaphylaxis: A Call to Action. Community Oncol..

[221] Riegert-Johnson D.L., Volcheck G.W. (2002). The Incidence of Anaphylaxis Following Intravenous Phytonadione (Vitamin K1): A 5-Year Retrospective Review. Ann. Allergy Asthma Immunol..

[222] Baker M.T., Naguib M. (2005). Propofol: The Challenges of Formulation. Anesthesiology.

[223] Ebo D.G., Piel G.C., Conraads V., Stevens W.J. (2001). IgE-Mediated Anaphylaxis after First Intravenous Infusion of Cyclosporine. Ann. Allergy Asthma Immunol..

[224] Jenkins D.J., Wolever T.M., Taylor R.H., Reynolds D., Nineham R., Hockaday T.D. (1980). Diabetic Glucose Control, Lipids, and Trace Elements on Long-Term Guar. Br. Med. J..

[225] Volcheck G.W., Van Dellen R.G. (1998). Anaphylaxis to Intravenous Cyclosporine and Tolerance to Oral Cyclosporine: Case Report and Review. Ann. Allergy Asthma Immunol..

[226] Wenande E., Garvey L.H. (2016). Immediate-Type Hypersensitivity to Polyethylene Glycols: A Review. Clin. Exp. Allergy.

[227] Rosenberg A.S. (2006). Effects of Protein Aggregates: An Immunologic Perspective. AAPS J..

[228] Moore W.V., Leppert P. (1980). Role of Aggregated Human Growth Hormone (HGH) in Development of Antibodies to HGH. J. Clin. Endocrinol. Metab..

[229] Baert F., Noman M., Vermeire S., Van Assche G., D’ Haens G., Carbonez A., Rutgeerts P. (2003). Influence of Immunogenicity on the Long-Term Efficacy of Infliximab in Crohn’s Disease. N. Engl. J. Med..

[230] Cox F., Khalib K., Conlon N. (2021). PEG That Reaction: A Case Series of Allergy to Polyethylene Glycol. J. Clin. Pharmacol..

[231] Nagarajan R. (1999). Solubilization of Hydrocarbons and Resulting Aggregate Shape Transitions in Aqueous Solutions of Pluronic® (PEO–PPO–PEO) Block Copolymers. Colloids Surf. B Biointerfaces.

[232] Kabanov A.V., Batrakova E.V., Alakhov V.Y. (2002). Pluronic Block Copolymers as Novel Polymer Therapeutics for Drug and Gene Delivery. J. Control. Release.

[233] Emanuele M., Balasubramaniam B. (2014). Differential Effects of Commercial-Grade and Purified Poloxamer 188 on Renal Function. Drugs R D.

[234] Strickley R.G., Lambert W.J. (2021). A Review of Formulations of Commercially Available Antibodies. J. Pharm. Sci..

[235] Uchiyama S. (2014). Liquid Formulation for Antibody Drugs. Biochim. Biophys. Acta.

[236] Gervasi V., Dall Agnol R., Cullen S., McCoy T., Vucen S., Crean A. (2018). Parenteral Protein Formulations: An Overview of Approved Products within the European Union. Eur. J. Pharm. Biopharm..

[237] (2016). Highlights of Prescribing Information: Norditropin® (Somatropin) Injection, for Subcutaneous Use. https://www.accessdata.fda.gov/drugsatfda_docs/label/2017/021148s049lbl.pdf.

[238] Erlandsson B. (2002). Stability-Indicating Changes in Poloxamers: The Degradation of Ethylene Oxide-Propylene Oxide Block Copolymers at 25 and 40 °C. Polym. Degrad. Stab..

[239] Gallet G., Carroccio S., Rizzarelli P., Karlsson S. (2002). Thermal Degradation of Poly(Ethylene Oxide–Propylene Oxide–Ethylene Oxide) Triblock Copolymer: Comparative Study by SEC/NMR, SEC/MALDI-TOF-MS and SPME/GC-MS. Polymer.

[240] Wang T., Markham A., Thomas S.J., Wang N., Huang L., Clemens M., Rajagopalan N. (2019). Solution Stability of Poloxamer 188 under Stress Conditions. J. Pharm. Sci..

[241] Grapentin C., Müller C., Kishore R.S.K., Adler M., ElBialy I., Friess W., Huwyler J., Khan T.A. (2020). Protein-Polydimethylsiloxane Particles in Liquid Vial Monoclonal Antibody Formulations Containing Poloxamer 188. J. Pharm. Sci..

[242] Wang T., Richard C.A., Dong X., Shi G.H. (2020). Impact of Surfactants on the Functionality of Prefilled Syringes. J. Pharm. Sci..

[243] Jiao J. (2008). Polyoxyethylated Nonionic Surfactants and Their Applications in Topical Ocular Drug Delivery. Adv. Drug Deliv. Rev..

[244] Sahoo R.K., Biswas N., Guha A., Sahoo N., Kuotsu K. (2014). Nonionic Surfactant Vesicles in Ocular Delivery: Innovative Approaches and Perspectives. Biomed. Res. Int..

[245] Mesiha M.S., El-Bitar H.I. (1981). Hypoglycaemic Effect of Oral Insulin Preparations Containing Brij 35, 52, 58 or 92 and Stearic Acid. J. Pharm. Pharmacol..

[246] Bam N.B., Randolph T.W., Cleland J.L. (1995). Stability of Protein Formulations: Investigation of Surfactant Effects by a Novel EPR Spectroscopic Technique. Pharm. Res..

[247] Jones L.S., Bam N.B., Randolph T.W. (1997). Surfactant-Stabilized Protein Formulations: A Review of Protein-Surfactant Interactions and Novel Analytical Methodologies. Therapeutic Protein and Peptide Formulation and Delivery.

[248] Krause M., Rudolph R., Schwarz E. (2002). The Non-Ionic Detergent Brij 58P Mimics Chaperone Effects. FEBS Lett..

[249] Yue L., Yan Z., Li H., Liu X., Sun P. (2020). Brij-58, a Potential Injectable Protein-Stabilizer Used in Therapeutic Protein Formulation. Eur. J. Pharm. Biopharm..

[250] Agarkhed M., O’Dell C., Hsieh M.-C., Zhang J., Goldstein J., Srivastava A. (2018). Effect of Surfactants on Mechanical, Thermal, and Photostability of a Monoclonal Antibody. AAPS PharmSciTech..

[251] Eng Y.Y., Sharma V.K., Ray A.K. (2010). Photocatalytic Degradation of Nonionic Surfactant, Brij 35 in Aqueous TiO2 Suspensions. Chemosphere.

[252] U.S. Government Accountability Office. (2005). Alkyl (C10–C16) Polyglycosides; Exemptions from the Requirement of a Tolerance.

[253] Maggio E.T. (2008). Stabilizing Alkylglycoside Compositions and Methods Thereof. U.S. Patent.

[254] Rifkin R.A., Maggio E.T., Dike S., Kerr D.A., Levy M. (2011). N-Dodecyl-β-D-Maltoside Inhibits Aggregation of Human Interferon-β-1b and Reduces Its Immunogenicity. J. Neuroimmune Pharm..

[255] Katz J.S., Tan Y., Kuppannan K., Song Y., Brennan D.J., Young T., Yao L., Jordan S. (2016). Amino-Acid-Incorporating Nonionic Surfactants for Stabilization of Protein Pharmaceuticals. ACS Biomater. Sci. Eng..

[256] Katz J.S., Nolin A., Yezer B.A., Jordan S. (2019). Dynamic Properties of Novel Excipient Suggest Mechanism for Improved Performance in Liquid Stabilization of Protein Biologics. Mol. Pharm..

[257] Katz J.S., Brennan D.J., Dan F., Tan Y., Jordan S.L., Young T.J., Song Y. (2018). Polyalkoxy Fatty Compound. U.S. Patent.

[258] Mensink M.A., Frijlink H.W., van der Voort Maarschalk K., Hinrichs W.L.J. (2017). How Sugars Protect Proteins in the Solid State and during Drying (Review): Mechanisms of Stabilization in Relation to Stress Conditions. Eur. J. Pharm. Biopharm..

[259] Olsson C., Swenson J. (2019). The Role of Disaccharides for Protein–Protein Interactions—A SANS Study. Mol. Phys..

[260] Carpenter J.F., Kendrick B.S., Chang B.S., Manning M.C., Randolph T.W. (1999). Inhibition of Stress-Induced Aggregation of Protein Therapeutics. Methods in Enzymology.

[261] Panzica M., Emanuele A., Cordone L. (2012). Thermal Aggregation of Bovine Serum Albumin in Trehalose and Sucrose Aqueous Solutions. J. Phys. Chem. B.

[262] Sun W.Q., Davidson P. (1998). Protein Inactivation in Amorphous Sucrose and Trehalose Matrices: Effects of Phase Separation and Crystallization. Biochim. Et Biophys. Acta (BBA)-Gen. Subj..

[263] Uritani M., Takai M., Yoshinaga K. (1995). Protective Effect of Disaccharides on Restriction Endonucleases during Drying under Vacuum. J. Biochem..

[264] Ohtake S., Wang Y.J. (2011). Trehalose: Current Use and Future Applications. J. Pharm. Sci..

[265] Schiefelbein L., Keller M., Weissmann F., Luber M., Bracher F., Frieß W. (2010). Synthesis, Characterization and Assessment of Suitability of Trehalose Fatty Acid Esters as Alternatives for Polysorbates in Protein Formulation. Eur. J. Pharm. Biopharm..

[266] Messina M.S., Ko J.H., Yang Z., Strouse M.J., Houk K.N., Maynard H.D. (2017). Effect of Trehalose Polymer Regioisomers on Protein Stabilization. Polym. Chem..

[267] Mastrotto F., Francini N., Allen S., van der Walle C.F., Stolnik S., Mantovani G. (2018). Synthetic Glycopolymers as Modulators of Protein Aggregation: Influences of Chemical Composition, Topology and Concentration. J. Mater. Chem. B.

[268] Mancini R.J., Lee J., Maynard H.D. (2012). Trehalose Glycopolymers for Stabilization of Protein Conjugates to Environmental Stressors. J. Am. Chem. Soc..

[269] Lee J., Lin E.-W., Lau U.Y., Hedrick J.L., Bat E., Maynard H.D. (2013). Trehalose Glycopolymers as Excipients for Protein Stabilization. Biomacromolecules.

[270] Liu Y., Lee J., Mansfield K.M., Ko J.H., Sallam S., Wesdemiotis C., Maynard H.D. (2017). Trehalose Glycopolymer Enhances Both Solution Stability and Pharmacokinetics of a Therapeutic Protein. Bioconjugate Chem..

[271] Schebor C., Burin L., del PilarBuera M., Chirife J. (1999). Stability to Hydrolysis and Browning of Trehalose, Sucrose and Raffinose in Low-Moisture Systems in Relation to Their Use as Protectants of Dry Biomaterials. LWT-Food Sci. Technol..

[272] Li J., Chen J., An L., Yuan X., Yao L. (2020). Polyol and Sugar Osmolytes Can Shorten Protein Hydrogen Bonds to Modulate Function. Commun. Biol..

[273] Timasheff S.N. (1998). Control of Protein Stability and Reactions by Weakly Interacting Cosolvents: The Simplicity of the Complicated. Adv. Protein Chem..

[274] Carpenter J.F., Crowe J.H. (1989). An Infrared Spectroscopic Study of the Interactions of Carbohydrates with Dried Proteins. Biochemistry.

[275] Vagenende V., Yap M.G.S., Trout B.L. (2009). Mechanisms of Protein Stabilization and Prevention of Protein Aggregation by Glycerol. Biochemistry.

[276] Jena S., Suryanarayanan R., Aksan A. (2016). Mutual Influence of Mannitol and Trehalose on Crystallization Behavior in Frozen Solutions. Pharm. Res..

[277] Cui Y., Cui P., Chen B., Li S., Guan H. (2017). Monoclonal Antibodies: Formulations of Marketed Products and Recent Advances in Novel Delivery System. Drug Dev. Ind. Pharm..

[278] Andya J.D., Maa Y.-F., Costantino H.R., Nguyen P.-A., Dasovich N., Sweeney T.D., Hsu C.C., Shire S.J. (1999). Effect of Formulation Excipients on Protein Stability and Aerosol Performance of Spray-Dried Powders of a Recombinant Humanized Anti-IgE Monoclonal Antibody1. Pharm. Res..

[279] Al-Hussein A., Gieseler H. (2012). The Effect of Mannitol Crystallization in Mannitol–Sucrose Systems on LDH Stability during Freeze-Drying. J. Pharm. Sci..

[280] Izutsu K., Yoshioka S., Terao T. (1994). Effect of Mannitol Crystallinity on the Stabilization of Enzymes during Freeze-Drying. Chem. Pharm. Bull..

[281] Izutsu K., Kojima S. (2002). Excipient Crystallinity and Its Protein-Structure-Stabilizing Effect during Freeze-Drying. J. Pharm. Pharmacol..

[282] Costantino H.R., Andya J.D., Nguyen P.-A., Dasovich N., Sweeney T.D., Shire S.J., Hsu C.C., Maa Y.-F. (1998). Effect of Mannitol Crystallization on the Stability and Aerosol Performance of a Spray-Dried Pharmaceutical Protein, Recombinant Humanized Anti-IgE Monoclonal Antibody. J. Pharm. Sci..

[283] Pyne A., Chatterjee K., Suryanarayanan R. (2003). Solute Crystallization in Mannitol–Glycine Systems—Implications on Protein Stabilization in Freeze-dried Formulations. J. Pharm. Sci..

[284] Thakral S., Sonje J., Munjal B., Bhatnagar B., Suryanarayanan R. (2022). Mannitol as an Excipient for Lyophilized Injectable Formulations. J. Pharm. Sci..

[285] Cleland J.L., Lam X., Kendrick B., Yang J., Yang T., Overcashier D., Brooks D., Hsu C., Carpenter J.F. (2001). A Specific Molar Ratio of Stabilizer to Protein Is Required for Storage Stability of a Lyophilized Monoclonal Antibody. J. Pharm. Sci..

[286] Wang S.S., Yan Y.S., Ho K. (2021). US FDA-Approved Therapeutic Antibodies with High-Concentration Formulation: Summaries and Perspectives. Antib. Ther..

[287] Piedmonte D.M., Summers C., McAuley A., Karamujic L., Ratnaswamy G. (2007). Sorbitol Crystallization Can Lead to Protein Aggregation in Frozen Protein Formulations. Pharm. Res..

[288] Piedmonte D.M., Hair A., Baker P., Brych L., Nagapudi K., Lin H., Cao W., Hershenson S., Ratnaswamy G. (2015). Sorbitol Crystallization-Induced Aggregation in Frozen MAb Formulations. J. Pharm. Sci..

[289] Davis J.M., Zhang N., Payne R.W., Murphy B.M., Abdul-Fattah A.M., Matsuura J.E., Herman A.C., Manning M.C. (2013). Stability of Lyophilized Sucrose Formulations of an IgG1: Subvisible Particle Formation. Pharm. Dev. Technol..

[290] Chang L.L., Shepherd D., Sun J., Tang X.C., Pikal M.J. (2005). Effect of Sorbitol and Residual Moisture on the Stability of Lyophilized Antibodies: Implications for the Mechanism of Protein Stabilization in the Solid State. J. Pharm. Sci..

[291] Rowe R.C., Quinn M.E. (2009). Handbook of Pharmaceutical Excipients.

[292] Kumar A. (2003). Adverse Effects of Pharmaceutical Excipients. Advers. Drug React. Bull..

[293] Pawar S., Kumar A. (2002). Issues in the Formulation of Drugs for Oral Use in Children: Role of Excipients. Paediatr. Drugs.

[294] Sweetman S.C. (2009). Martindale: The Complete Drug Reference.

[295] Hägnevik K., Gordon E., Lins L.E., Wilhelmsson S., Forster D. (1974). Glycerol-Induced Haemolysis with Haemoglobinuria and Acute Renal Failure. Report of Three Cases. Lancet.

[296] Welch K.M., Meyer J.S., Okamoto S., Mathew N.T., Rivera V.M., Bond J. (1974). Letter: Glycerol-Induced Haemolysis. Lancet.

[297] Staples R., Misher A., Wardell J. (1967). Gastrointestinal Irritant Effect of Glycerin as Compared with Sorbitol and Propylene Glycol in Rats and Dogs. J. Pharm. Sci..

[298] Porter G.A., Starr A., Kimsey J., Lenertz H. (1967). Mannitol Hemodilution-Perfusion: The Kinetics of Mannitol Distribution and Excretion during Cardiopulmonary Bypass. J. Surg. Res..

[299] Safety Assessment of Mannitol, Sorbitol, and Xylitol as Used in Cosmetics 2019. https://www.cir-safety.org/sites/default/files/Mannitol,%20Sorbitol,%20Xylitol_0.pdf.

[300] McNeill I.Y. (1985). Hypersensitivity Reaction to Mannitol. Drug Intell. Clin. Pharm..

[301] Lamb J.D., Keogh J.A. (1979). Anaphylactoid Reaction to Mannitol. Can. Anaesth Soc. J..

[302] Spaeth G.L., Spaeth E.B., Spaeth P.G., Lucier A.C. (1967). Anaphylactic Reaction to Mannitol. Arch. Ophthalmol..

[303] Serno T., Geidobler R., Winter G. (2011). Protein Stabilization by Cyclodextrins in the Liquid and Dried State. Adv. Drug Deliv. Rev..

[304] Vyas A., Saraf S., Saraf S. (2008). Cyclodextrin Based Novel Drug Delivery Systems. J. Incl. Phenom. Macrocycl. Chem..

[305] Gidwani B., Vyas A. (2015). A Comprehensive Review on Cyclodextrin-Based Carriers for Delivery of Chemotherapeutic Cytotoxic Anticancer Drugs. Biomed. Res. Int..

[306] Ferreira L., Campos J., Veiga F., Cardoso C., Paiva-Santos A.C. (2022). Cyclodextrin-Based Delivery Systems in Parenteral Formulations: A Critical Update Review. Eur. J. Pharm. Biopharm..

[307] Charman S.A., Mason K.L., Charman W.N. (1993). Techniques for Assessing the Effects of Pharmaceutical Excipients on the Aggregation of Porcine Growth Hormone. Pharm. Res..

[308] Tavornvipas S., Tajiri S., Hirayama F., Arima H., Uekama K. (2004). Effects of Hydrophilic Cyclodextrins on Aggregation of Recombinant Human Growth Hormone. Pharm. Res..

[309] Taneri F., Güneri T., Aigner Z., Kata M. (2002). Improvement in the Physicochemical Properties of Ketoconazole through Complexation with Cyclodextrin Derivatives. J. Incl. Phenom..

[310] Yoshida A., Arima H., Uekama K., Pitha J. (1988). Pharmaceutical Evaluation of Hydroxyalkyl Ethers of β-Cyclodextrins. Int. J. Pharm..

[311] Samra H.S., He F., Bhambhani A., Pipkin J.D., Zimmerer R., Joshi S.B., Middaugh C.R. (2010). The Effects of Substituted Cyclodextrins on the Colloidal and Conformational Stability of Selected Proteins. J. Pharm. Sci..

[312] Serno T., Carpenter J.F., Randolph T.W., Winter G. (2010). Inhibition of Agitation-Induced Aggregation of an IgG-Antibody by Hydroxypropyl-Beta-Cyclodextrin. J. Pharm. Sci..

[313] Wu H.H., Garidel P., Michaela B. (2021). HP-β-CD for the Formulation of IgG and Ig-Based Biotherapeutics. Int. J. Pharm..

[314] Aachmann F.L., Otzen D.E., Larsen K.L., Wimmer R. (2003). Structural Background of Cyclodextrin–Protein Interactions. Protein Eng. Des. Sel..

[315] Zhang H., Hong S., Tan S.S.K., Peng T., Goh L.Y.H., Lam K.H., Chow K.T., Gokhale R. (2022). Polysorbates versus Hydroxypropyl Beta-Cyclodextrin (HPβCD): Comparative Study on Excipient Stability and Stabilization Benefits on Monoclonal Antibodies. Molecules.

[316] Stam M.R., Danchin E.G.J., Rancurel C., Coutinho P.M., Henrissat B. (2006). Dividing the Large Glycoside Hydrolase Family 13 into Subfamilies: Towards Improved Functional Annotations of Alpha-Amylase-Related Proteins. Protein Eng. Des. Sel..

[317] Karginov V.A. (2013). Cyclodextrin Derivatives as Anti-Infectives. Curr. Opin. Pharmacol..

[318] Frömming K.-H., Szejtli J. (1994). Cyclodextrins in Pharmacy.

[319] Buedenbender S., Schulz G.E. (2009). Structural Base for Enzymatic Cyclodextrin Hydrolysis. J. Mol. Biol..

[320] Atwood J.L., Lehn J.-M., Szejtli J. (1996). The Metabolism, Toxicity Abd Biological Effects of Cyclodextrins. Comprehensive Supramolecular Chemistry.

[321] Munro I.C., Newberne P.M., Young V.R., Bär A. (2004). Safety Assessment of Gamma-Cyclodextrin. Regul. Toxicol. Pharmacol..

[322] Jodál I., Kandra L., Harangi J., Nánási P., Szejtli J. (1984). Enzymatic Degradation of Cyclodextrins; Preparation and Application of Their Fragments. J. Incl. Phenom..

[323] Turner P., Labes A., Fridjonsson O.H., Hreggvidson G.O., Schönheit P., Kristjansson J.K., Holst O., Karlsson E.N. (2005). Two Novel Cyclodextrin-Degrading Enzymes Isolated from Thermophilic Bacteria Have Similar Domain Structures but Differ in Oligomeric State and Activity Profile. J. Biosci. Bioeng..

[324] Connolly B., HAMBURG L., HOLZ E. (2017). Formulations with Reduced Degradation of Polysorbate. French Patent.

[325] Uchida K., Kawakishi S. (1986). Oxidative Degradation of β-Cyclodextrin Induced by an Ascorbic Acid-Copper Ion System. Agric. Biol. Chem..

[326] Mamani P.L., Ruiz-Caro R., Veiga M.D. (2012). Matrix Tablets: The Effect of Hydroxypropyl Methylcellulose/Anhydrous Dibasic Calcium Phosphate Ratio on the Release Rate of a Water-Soluble Drug through the Gastrointestinal Tract I. in vitro Tests. AAPS PharmSciTech..

[327] Ishikawa T., Watanabe Y., Takayama K., Endo H., Matsumoto M. (2000). Effect of Hydroxypropylmethylcellulose (HPMC) on the Release Profiles and Bioavailability of a Poorly Water-Soluble Drug from Tablets Prepared Using Macrogol and HPMC. Int. J. Pharm..

[328] Mašková E., Kubová K., Raimi-Abraham B.T., Vllasaliu D., Vohlídalová E., Turánek J., Mašek J. (2020). Hypromellose-A Traditional Pharmaceutical Excipient with Modern Applications in Oral and Oromucosal Drug Delivery. J. Control. Release.

[329] CiNii (1986). Final Report on the Safety Assessment of Hydroxyethylcellulose, Hydroxypropylcellulose, Methylcellulose, Hydroxypropyl Methylcellulose, and Cellulose Gum. J. Am. Coll. Toxicol..

[330] Soane D.S., Mahoney R.P., Wuthrich P., GREENE D.G. (2019). Stabilizing Excipients for Therapeutic Protein Formulations. French Patent.

[331] Lynch S., Turowski M., Yokoyama W., Hong Y., Conklin J., Hung S., Young S. (2008). Uses of Water-Soluble Cellulose Derivatives for Preventing or Treating Metabolic Syndrome. French Patent.

[332] Sasahara K., McPhie P., Minton A.P. (2003). Effect of Dextran on Protein Stability and Conformation Attributed to Macromolecular Crowding. J. Mol. Biol..

[333] Flood A., Estrada M., McAdams D., Ji Y., Chen D. (2016). Development of a Freeze-Dried, Heat-Stable Influenza Subunit Vaccine Formulation. PLoS ONE.

[334] Sun W.Q., Davidson P. (2001). Effect of Dextran Molecular Weight on Protein Stabilization during Freeze-Drying and Storage. Cryo Lett..

[335] Jones B., Mahajan A., Aksan A. (2018). Dextranol: A Better Lyoprotectant. bioRxiv.

[336] Rudin C., Günthard J., Halter C., Staehelin J., Berglund A. (1995). Anaphylactoid Reaction to BCG Vaccine Containing High Molecular Weight Dextran. Eur. J. Pediatr..

[337] Uchida S., Takekawa D., Kitayama M., Hirota K. (2022). Two Cases of Circulatory Collapse Due to Suspected Remimazolam Anaphylaxis. JA Clin. Rep..

[338] Bozorgmehr M.R., Monhemi H. (2015). How Can a Free Amino Acid Stabilize a Protein? Insights from Molecular Dynamics Simulation. J. Solut. Chem..

[339] Bruździak P., Panuszko A., Kaczkowska E., Piotrowski B., Daghir A., Demkowicz S., Stangret J. (2018). Taurine as a Water Structure Breaker and Protein Stabilizer. Amino Acids.

[340] Platts L., Falconer R.J. (2015). Controlling Protein Stability: Mechanisms Revealed Using Formulations of Arginine, Glycine and Guanidinium HCl with Three Globular Proteins. Int. J. Pharm..

[341] Shiraki K., Kudou M., Fujiwara S., Imanaka T., Takagi M. (2002). Biophysical Effect of Amino Acids on the Prevention of Protein Aggregation. J. Biochem..

[342] Mojtabavi S., Samadi N., Faramarzi M.A. (2019). Osmolyte-Induced Folding and Stability of Proteins: Concepts and Characterization. Iran. J. Pharm. Res..

[343] Arakawa T., Tsumoto K. (2003). The Effects of Arginine on Refolding of Aggregated Proteins: Not Facilitate Refolding, but Suppress Aggregation. Biochem. Biophys. Res. Commun..

[344] Inoue N., Takai E., Arakawa T., Shiraki K. (2014). Arginine and Lysine Reduce the High Viscosity of Serum Albumin Solutions for Pharmaceutical Injection. J. Biosci. Bioeng..

[345] Inoue N., Takai E., Arakawa T., Shiraki K. (2014). Specific Decrease in Solution Viscosity of Antibodies by Arginine for Therapeutic Formulations. Mol. Pharm..

[346] Das U., Hariprasad G., Ethayathulla A.S., Manral P., Das T.K., Pasha S., Mann A., Ganguli M., Verma A.K., Bhat R. (2007). Inhibition of Protein Aggregation: Supramolecular Assemblies of Arginine Hold the Key. PLoS ONE.

[347] Shukla D., Trout B.L. (2010). Interaction of Arginine with Proteins and the Mechanism by Which It Inhibits Aggregation. J. Phys. Chem. B.

[348] Stärtzel P. (2018). Arginine as an Excipient for Protein Freeze-Drying: A Mini Review. J. Pharm. Sci..

[349] Falconer R.J., Chan C., Hughes K., Munro T.P. (2011). Stabilization of a Monoclonal Antibody during Purification and Formulation by Addition of Basic Amino Acid Excipients. J. Chem. Technol. Biotechnol..

[350] Holeček M. (2020). Histidine in Health and Disease: Metabolism, Physiological Importance, and Use as a Supplement. Nutrients.

[351] Bramham J.E., Davies S.A., Podmore A., Golovanov A.P. (2021). Stability of a High-Concentration Monoclonal Antibody Solution Produced by Liquid–Liquid Phase Separation. MAbs.

[352] Burnett C.L., Heldreth B., Bergfeld W.F., Belsito D.V., Hill R.A., Klaassen C.D., Liebler D.C., Marks J.G., Shank R.C., Slaga T.J. (2013). Safety Assessment of α-Amino Acids as Used in Cosmetics. Int. J. Toxicol..

[353] Label: R-Gene® 10 Arginine Hydrochloride Injection, USP for Intravenous Use. https://labeling.pfizer.com/ShowLabeling.aspx?id=642.

[354] Tangphao O., Grossmann M., Chalon S., Hoffman B.B., Blaschke T.F. (1999). Pharmacokinetics of Intravenous and Oral L-Arginine in Normal Volunteers. Br. J. Clin. Pharmacol..

[355] Bode-Böger S.M., Böger R.H., Creutzig A., Tsikas D., Gutzki F.M., Alexander K., Frölich J.C. (1994). L-Arginine Infusion Decreases Peripheral Arterial Resistance and Inhibits Platelet Aggregation in Healthy Subjects. Clin. Sci..

[356] Registration Dossier-ECHA. https://echa.europa.eu/de/registration-dossier/-/registered-dossier/13725/7/3/1.

[357] Tsubuku S., Hatayama K., Mawatari K., Smriga M., Kimura T. (2004). Thirteen-Week Oral Toxicity Study of l-Arginine in Rats. Int. J. Toxicol..

[358] Tsai-Turton M. (2016). Pharmacology/Toxicology NDA/BLA Review and Evaluation.

[359] Minois N., Carmona-Gutierrez D., Madeo F. (2011). Polyamines in Aging and Disease. Aging.

[360] Kudou M., Shiraki K., Fujiwara S., Imanaka T., Takagi M. (2003). Prevention of Thermal Inactivation and Aggregation of Lysozyme by Polyamines. Eur. J. Biochem..

[361] Okanojo M., Shiraki K., Kudou M., Nishikori S., Takagi M. (2005). Diamines Prevent Thermal Aggregation and Inactivation of Lysozyme. J. Biosci. Bioeng..

[362] Schwarz C., Stekovic S., Wirth M., Benson G., Royer P., Sigrist S.J., Pieber T., Dammbrueck C., Magnes C., Eisenberg T. (2018). Safety and Tolerability of Spermidine Supplementation in Mice and Older Adults with Subjective Cognitive Decline. Aging.

[363] Til H.P., Falke H.E., Prinsen M.K., Willems M.I. (1997). Acute and Subacute Toxicity of Tyramine, Spermidine, Spermine, Putrescine and Cadaverine in Rats. Food Chem. Toxicol..

[364] Truong-Le V., Lovalenti P.M., Abdul-Fattah A.M. (2015). Stabilization Challenges and Formulation Strategies Associated with Oral Biologic Drug Delivery Systems. Adv. Drug Deliv. Rev..

[365] Sönksen P.H., Ellis J.P., Lowy C., Rutherford A., Nabarro J.D.N. (1966). A Quantitative Evaluation of the Relative Efficiency of Gelatine and Albumin in Preventing Insulin Adsorption to Glass. Diabetologia.

[366] Jeyachandran Y.L., Mielczarski E., Rai B., Mielczarski J.A. (2009). Quantitative and Qualitative Evaluation of Adsorption/Desorption of Bovine Serum Albumin on Hydrophilic and Hydrophobic Surfaces. Langmuir.

[367] Haeuser C., Goldbach P., Huwyler J., Friess W., Allmendinger A. (2020). Excipients for Room Temperature Stable Freeze-Dried Monoclonal Antibody Formulations. J. Pharm. Sci..

[368] Sønderby P., Bukrinski J.T., Hebditch M., Peters G.H.J., Curtis R.A., Harris P. (2018). Self-Interaction of Human Serum Albumin: A Formulation Perspective. ACS Omega.

[369] Castro L.S., Lobo G.S., Pereira P., Freire M.G., Neves M.C., Pedro A.Q. (2021). Interferon-Based Biopharmaceuticals: Overview on the Production, Purification, and Formulation. Vaccines.

[370] Taverna M., Marie A.-L., Mira J.-P., Guidet B. (2013). Specific Antioxidant Properties of Human Serum Albumin. Ann. Intensive Care.

[371] Chou D.K., Krishnamurthy R., Manning M.C., Randolph T.W., Carpenter J.F. (2012). Physical Stability of Albinterferon-α(2b) in Aqueous Solution: Effects of Conformational Stability and Colloidal Stability on Aggregation. J. Pharm. Sci..

[372] Tarelli E., Mire-Sluis A., Tivnann H.A., Bolgiano B., Crane D.T., Gee C., Lemercinier X., Athayde M.L., Sutcliffe N., Corran P.H. (1998). Recombinant Human Albumin as a Stabilizer for Biological Materials and for the Preparation of International Reference Reagents. Biologicals.

[373] Rajan R.S., Li T., Aras M., Sloey C., Sutherland W., Arai H., Briddell R., Kinstler O., Lueras A.M.K., Zhang Y. (2006). Modulation of Protein Aggregation by Polyethylene Glycol Conjugation: GCSF as a Case Study. Protein Sci..

[374] Chao S.-H., Matthews S.S., Paxman R., Aksimentiev A., Gruebele M., Price J.L. (2014). Two Structural Scenarios for Protein Stabilization by PEG. J. Phys. Chem. B.

[375] Rawat S., Raman Suri C., Sahoo D.K. (2010). Molecular Mechanism of Polyethylene Glycol Mediated Stabilization of Protein. Biochem. Biophys. Res. Commun..

[376] Vrkljan M., Foster T.M., Powers M.E., Henkin J., Porter W.R., Staack H., Carpenter J.F., Manning M.C. (1994). Thermal Stability of Low Molecular Weight Urokinase during Heat Treatment. II. Effect of Polymeric Additives. Pharm. Res..

[377] El-hoshoudy A.N. (2021). Experimental and Theoretical Investigation of Glycol-Based Hydrogels through Waterflooding Processes in Oil Reservoirs Using Molecular Dynamics and Dissipative Particle Dynamics Simulation. ACS Omega.

[378] McCallen J., Prybylski J., Yang Q., Lai S.K. (2017). Cross-Reactivity of Select PEG-Binding Antibodies to Other Polymers Containing a C-C-O Backbone. ACS Biomater. Sci. Eng..

[379] Rajan R., Ahmed S., Sharma N., Kumar N., Debas A., Matsumura K. (2021). Review of the Current State of Protein Aggregation Inhibition from a Materials Chemistry Perspective: Special Focus on Polymeric Materials. Mater. Adv..

[380] Rajan R., Matsumura K. (2017). Inhibition of Protein Aggregation by Zwitterionic Polymer-Based Core-Shell Nanogels. Sci. Rep..

[381] Rajan R., Suzuki Y., Matsumura K. (2018). Zwitterionic Polymer Design That Inhibits Aggregation and Facilitates Insulin Refolding: Mechanistic Insights and Importance of Hydrophobicity. Macromol. Biosci..

[382] Zhao D., Rajan R., Matsumura K. (2019). Dual Thermo- and PH-Responsive Behavior of Double Zwitterionic Graft Copolymers for Suppression of Protein Aggregation and Protein Release. ACS Appl. Mater. Interfaces.

[383] Reslan M., Kayser V. (2018). Ionic Liquids as Biocompatible Stabilizers of Proteins. Biophys. Rev..

[384] Shukla S.K., Mikkola J.-P. (2020). Use of Ionic Liquids in Protein and DNA Chemistry. Front. Chem..

[385] Patel R., Kumari M., Khan A.B. (2014). Recent Advances in the Applications of Ionic Liquids in Protein Stability and Activity: A Review. Appl. Biochem. Biotechnol..

[386] Bui-Le L., Clarke C.J., Bröhl A., Brogan A.P.S., Arpino J.A.J., Polizzi K.M., Hallett J.P. (2020). Revealing the Complexity of Ionic Liquid–Protein Interactions through a Multi-Technique Investigation. Commun. Chem..

[387] Kumar A., Venkatesu P. (2012). Prevention of Insulin Self-Aggregation by a Protic Ionic Liquid. RSC Adv..

[388] Summers C.A., Flowers II R.A. (2000). Protein Renaturation by the Liquid Organic Salt Ethylammonium Nitrate. Protein Sci..

[389] Bisht M., Kumar A., Venkatesu P. (2016). Refolding Effects of Partially Immiscible Ammonium-Based Ionic Liquids on the Urea-Induced Unfolded Lysozyme Structure. Phys. Chem. Chem. Phys..

[390] Mann J.P., McCluskey A., Atkin R. (2009). Activity and Thermal Stability of Lysozyme in Alkylammonium Formate Ionic Liquids—Influence of Cation Modification. Green Chem..

[391] Satish L., Rana S., Arakha M., Rout L., Ekka B., Jha S., Dash P., Sahoo H. (2016). Impact of Imidazolium-Based Ionic Liquids on the Structure and Stability of Lysozyme. Spectrosc. Lett..

[392] Singh U.K., Kumari M., Khan S.H., Bohidar H.B., Patel R. (2018). Mechanism and Dynamics of Long-Term Stability of Cytochrome c Conferred by Long-Chain Imidazolium Ionic Liquids at Low Concentration. ACS Sustain. Chem. Eng..

[393] Lange C., Patil G., Rudolph R. (2005). Ionic Liquids as Refolding Additives: N′-Alkyl and N′-(ω-Hydroxyalkyl) N-Methylimidazolium Chlorides. Protein Sci..

[394] Sindhu A., Bhakuni K., Sankaranarayanan K., Venkatesu P. (2020). Implications of Imidazolium-Based Ionic Liquids as Refolding Additives for Urea-Induced Denatured Serum Albumins. ACS Sustain. Chem. Eng..

[395] Constatinescu D., Herrmann C., Weingärtner H. (2010). Patterns of Protein Unfolding and Protein Aggregation in Ionic Liquids. Phys. Chem. Chem. Phys..

[396] Zhao H. (2016). Protein Stabilization and Enzyme Activation in Ionic Liquids: Specific Ion Effects. J. Chem. Technol. Biotechnol..

[397] Bisht M., Kumar A., Venkatesu P. (2015). Analysis of the Driving Force That Rule the Stability of Lysozyme in Alkylammonium-Based Ionic Liquids. Int. J. Biol. Macromol..

[398] Baker G.A., Heller W.T. (2009). Small-Angle Neutron Scattering Studies of Model Protein Denaturation in Aqueous Solutions of the Ionic Liquid 1-Butyl-3-Methylimidazolium Chloride. Chem. Eng. J..

[399] Kumar A., Venkatesu P. (2014). The Stability of Insulin in the Presence of Short Alkyl Chain Imidazolium-Based Ionic Liquids. RSC Adv..

[400] Kumar P.K., Jha I., Venkatesu P., Bahadur I., Ebenso E.E. (2017). A Comparative Study of the Stability of Stem Bromelain Based on the Variation of Anions of Imidazolium-Based Ionic Liquids. J. Mol. Liq..

[401] Ajloo D., Sangian M., Ghadamgahi M., Evini M., Saboury A.A. (2013). Effect of Two Imidazolium Derivatives of Ionic Liquids on the Structure and Activity of Adenosine Deaminase. Int. J. Biol. Macromol..

[402] Veríssimo N.V., Saponi C.F., Ryan T.M., Greaves T.L., Pereira J.F.B. (2021). Imidazolium-Based Ionic Liquids as Additives to Preserve the Enhanced Green Fluorescent Protein Fluorescent Activity. Green Chem. Eng..

[403] Cheng K., Wu Q., Jiang L., Liu M., Li C. (2019). Protein Stability Analysis in Ionic Liquids by 19F NMR. Anal. Bioanal. Chem..

[404] Roethlisberger D., Mahler H.-C., Altenburger U., Pappenberger A. (2017). If Euhydric and Isotonic Do Not Work, What Are Acceptable PH and Osmolality for Parenteral Drug Dosage Forms?. J. Pharm. Sci..

[405] Foureau D.M., Vrikkis R.M., Jones C.P., Weaver K.D., Macfarlane D.R., Salo J.C., McKillop I.H., Elliott G.D. (2012). In Vitro Assessment of Choline Dihydrogen Phosphate (CDHP) as a Vehicle for Recombinant Human Interleukin-2 (RhIL-2). Cell Mol. Bioeng..

[406] Nony P., Girard P., Chabaud S., Hessel L., Thébault C., Boissel J.P. (2001). Impact of Osmolality on Burning Sensations during and Immediately after Intramuscular Injection of 0.5 Ml of Vaccine Suspensions in Healthy Adults. Vaccine.

[407] Kumar A., Bisht M., Jha I., Venkatesu P. (2017). The Role of Ionic Liquids in Protein Folding/ Unfolding Studies. Progress and Developments in Ionic Liquids.

[408] Gonçalves A.R.P., Paredes X., Cristino A.F., Santos F.J.V., Queirós C.S.G.P. (2021). Ionic Liquids—A Review of Their Toxicity to Living Organisms. Int. J. Mol. Sci..

[409] Thuy Pham T.P., Cho C.-W., Yun Y.-S. (2010). Environmental Fate and Toxicity of Ionic Liquids: A Review. Water Res..

[410] Musiał M., Zorębski E., Malarz K., Kuczak M., Mrozek-Wilczkiewicz A., Jacquemin J., Dzida M. (2021). Cytotoxicity of Ionic Liquids on Normal Human Dermal Fibroblasts in the Context of Their Present and Future Applications. ACS Sustain. Chem. Eng..

[411] Gouveia W., Jorge T.F., Martins S., Meireles M., Carolino M., Cruz C., Almeida T.V., Araújo M.E.M. (2014). Toxicity of Ionic Liquids Prepared from Biomaterials. Chemosphere.

[412] Moshikur R.M., Chowdhury M.R., Moniruzzaman M., Goto M. (2020). Biocompatible Ionic Liquids and Their Applications in Pharmaceutics. Green Chem..

[413] Weaver K.D., Kim H.J., Sun J., MacFarlane D.R., Elliott G.D. (2010). Cyto-Toxicity and Biocompatibility of a Family of Choline Phosphate Ionic Liquids Designed for Pharmaceutical Applications. Green Chem..

[414] Raiguel S., Dehaen W., Binnemans K. (2020). Stability of Ionic Liquids in Brønsted-Basic Media. Green Chem..

