# Supplementary material for: Alternative Excipients for Protein Stabilization in Protein Therapeutics: Overcoming the Limitations of Polysorbates
Source: Pharmaceutics. 2022 Nov 23;14(12):2575. doi: 10.3390/pharmaceutics14122575 (PMC9781097; doi:10.3390/pharmaceutics14122575)
Supplement: Supplementary file 1 [file pharmaceutics-14-02575-s001.zip › pharmaceutics-2010958-Supplementary.pdf]

Supplementary Information

Table S1. FDA and CBER-approved protein biopharmaceuticals containing PS20 and PS80 as a surfactant indicating the amount of surfactant per mL. Data obtained from https://www.accessdata.fda.gov/scripts/cder/iig/index.cfm (accessed on 10 August 2022).

Commercial name	FDA Approval year	Biotherapeutic type	Route of administration	Surfactant	Concentration
ACTIVASE (alteplase)	1987	a recombinant a tissue plasminogen activator	IV	PS80	0.1 mg/mL
NEUPOGEN® (filgrastim)	1991	a recombinant 175 amino acid human granulocyte colony-stimulating factor	SC, IV	PS80	0.04 mg/mL
CEREZYME® (imiglucerase)	1994	an analogue of the human enzyme ß-glucocerebrosidase, a lysosomal glycoprotein enzyme which catalyzes the hydrolysis of the glycolipid glucocerebroside to glucose and ceramide	IV	PS80	0.1 mg/mL
ReoPro® (abciximab)	1994	fragment of the chimeric human-murine 7E3 mAb binding to the glycoprotein IIb/IIIa receptor of human platelets as well as the vitronectin (αvβ3) receptor on platelets, vessel wall endothelial, and smooth muscle cells	IV	PS80	0.01 mg/mL
RITUXAN (rituximab)	1997	a genetically engineered chimeric murine/human mAb (IgG1κ) directed against the CD20 antigen	IV	PS80	0.7 mg/mL
ZENAPAX® (daclizumab)	1997	humanized mAb (IgG1) binding specifically to the alpha subunit (p55 alpha, CD25, or Tac subunit) of the human high-affinity interleukin-2 receptor expressed on the surface of activated lymphocytes	IV	PS80	0.2 mg/mL
HERCEPTIN (Trastuzumab)	1998	a recombinant humanized mAb (IgG1κ) selectively and effectively binding to the extracellular domain of the human epidermal growth factor receptor 2 protein	IV	PS20	0.09 mg/mL
REMICADE (infliximab)	1998	a chimeric mAb (IgG1κ; composed of human constant and murine variable regions) specific for human tumor necrosis factor-alpha	IV	PS80	0.05 mg/mL
CAMPATH® (alemtuzumab)	2001	a recombinant humanized mAb against the cell surface glycoprotein CD52	IV	PS80	0.1mg/mL
LEMTRADA® (alemtuzumab)	2001	a recombinant humanized mAb against the cell surface glycoprotein CD52	IV	PS80	0.1 mg/mL
HUMIRA® (adalimumab)	2002	a recombinant human mAb (IgG1) specific for human tumor necrosis factor	SC	PS80	1 mg/mL
PEGASYS (peginterferon alfa-2a)	2002	a covalent conjugate of recombinant alfa-2a interferon with a single branched bis-monomethoxy polyethylene glycol	SC	PS80	0.05 mg/mL
XOLAIR® (omalizumab)	2003	A recombinant humanized mAb (IgGκ) binding to human immunoglobulin E	SC	PS20	0.36 mg/mL
AVASTIN® (bevacizumab)	2004	a recombinant humanized mAb (IgG1) binding to and inhibiting the biologic activity of human vascular endothelial growth factor	IV	PS20	0.4 mg/mL
TYSABRI (natalizumab)	2004	a recombinant humanized mAb (IgG4κ) binding to binds to α4-integrin	IV	PS80	0.2 mg/mL
LUCENTIS® (ranibizumab injection)	2006	a recombinant humanized mAb (IgGκ) binding to and inhibiting the biologic activity of human vascular endothelial growth factor A	IVT	PS20	0.1 mg/mL

SOLIRIS® (eculizumab)	2007	a recombinant humanized mAb (IgG2/4κ) inhibiting the complement	IV	PS80	0.22 mg/mL
CIMZIA (certolizumab pegol)	2008	a recombinant, humanized Ab Fab' fragment, with specificity for human tumor necrosis factor alpha	SC	PS20	0.1 mg/mL
Nplate® (romiplostim)	2008	an Fc-peptide fusion protein (peptibody) that activates intracellular transcriptional pathways leading to increased platelet production via the thrombopoietin receptor	SC	PS20	0.042 mg/mL
ARZERRA® (ofatumumab)	2009	A humanized mAb (IgG1κ) binding specifically to both the small and large extracellular loops of the CD20 molecule	IV	PS80	0.2 mg/mL
ILARIS® (canakinumab)	2009	human mAb (IgG1κ) binding and inhibiting the activity of human interleukin-1β	SC	PS80	0.6 mg/mL
SIMPONI ARIA® (golimumab)	2009	Human mAb (IgG1κ) specific for human tumor necrosis factor alpha	SC, IV	PS80	0.15 mg/mL
STELARA® (ustekinumab)	2009	a human mAb (IgG1κ) binding with specificity to the p40 protein subunit used by both the interleukin-12 and interleukin-23	SC, IV	PS80	0.04 mg/mL
ACTEMRA® (tocilizumab)	2010	a recombinant humanized mAb (IgG1κ) binding to interleukin 6 receptor	SC, IV	PS80	0.5 mg/mL
Prolia® (denosumab)	2010	a human mAb (IgG2) with affinity and specificity for human receptor activator of nuclear factor kappa-B ligand	SC	PS20	0.1 mg/mL
ADCETRIS® (brentuximab vedotin)	2011	an CD30-directed antibody-drug conjugate consisting a chimeric AB (IgG1) specific for human CD30, the microtubule disrupting agent monomethyl auristatin E (MMAE) covalently bond together through a protease-cleavable linker	IV	PS80	0.2 mg/mL
BENLYSTA (belimumab)	2011	a human IgG1λ mAb specific for soluble human B lymphocyte stimulator protein	SC, IV	PS80	0.4 mg/mL
EYLEA™ (aflibercept)	2011	a recombinant fusion protein (portions of human vascular endothelial growth factor receptors 1 and 2 extracellular domains fused to the constant fragment of human IgG1)	IVT	PS20	0.3 mg/mL
YERVOY® (ipilimuma)	2011	a recombinant, human mAb (IgG1κ) binding to the cytotoxic tlymphocyte-associated antigen 4	IV	PS80	0.1 mg/mL
ELELYSO (taliglucerase alfa)	2012	a hydrolytic lysosomal glucocerebroside-specific enzyme catalyzing the hydrolysis of the glycolipid glucocerebroside to glucose and ceramide	IV	PS80	0.11 mg/mL
GRANIX® (tbo-filgrastim)	2012	a non-glycosylated recombinant methionyl human granulocyte colony-stimulating growth factor	SC	PS80	0.055 mg/mL
PERJETA (pertuzumab)	2012	a recombinant humanized mAb targeting the subdomain II of the human epidermal growth factor receptor 2	IV	PS20	0.2 mg/mL
RAXIBACUMAB	2012	a human mAb (IgG1λ) binding to the protective antigen component of B. anthracis toxin	IV	PS80	0.2 mg/mL
ZALTRAP® (ziv-aflibercept)	2012	a recombinant fusion protein (portions of human vascular endothelial growth factor receptors 1 and 2 extracellular domains fused to the constant fragment of human IgG1)	IV	PS20	1 mg/mL
KADCYLA® (ado-trastuzumab emtansine)	2013	trastuzumab (humanized mAb (IgG)) covalently linked to the microtubule inhibitory drug DM1 (a maytansine derivative) via the stable thioether linker MCC (4-[N-maleimidomethyl] cyclohexane-1-carboxylate)	IV	PS20	0.2 mg/mL
NOVOEIGHT	2013	recombinant antihemophilic factor	IV	PS80	0.1 mg/mL
RIXUBIS	2013	recombinant coagulation factor IX	IV	PS80	0.05 mg/mL

TRETTEN®	2013	recombinant coagulation factor XIII A-subunit	IV	PS20	0.1 mg/mL
ALPROLIX®	2014	recombinant coagulation factor IX, Fc fusion protein	IV	PS20	Not available
BLINCYTO® (blinatumomab)	2014	a bispecific CD19-directed CD3 T-cell engager	IV	PS80	0.213 mg/mL
CYRAMZA (ramucirumab)	2014	a recombinant human mAb (IgG1) against vascular endothelial growth factor receptor 2	IV	PS80	0.1 mg/mL
ELOCTATE®	2014	recombinant antihemophilic factor, Fc fusion protein	IV	PS20	Not available
ENTYVIO (vedolizumab)	2014	a humanized mAb (IgG1) binding to the human α4β7 integrin	IV	PS80	0.625 mg/mL
KEYTRUDA® (pembrolizumab)	2014	a humanized mAb (IgG4κ) blocking the programmed death receptor-1	IV	PS80	0.2 mg/mL
MYALEPT™ (metreleptin)	2014	a recombinant human leptin analog binding to and activates the leptin receptor	SC	PS20	0.1 mg/mL
OBIZUR		recombinant antihemophilic factor, porcine sequence	IV	PS80	0.05 mg/mL
OPDIVO (nivolumab)	2014	a mAb (IgG4κ) blocking the programmed death receptor-1	IV	PS80	0.2 mg/mL
PLEGRIDY (peginterferon beta-1a)	2014	an interferon beta-1a covalently bond through the alpha amino group of the N-terminal amino acid residue to a single, linear 20,000 dalton methoxy poly(ethyleneglycol)-O-2-methylpropionaldehyde molecule	SC	PS20	0.05 mg/mL
SYLVANT (siltuximab)	2014	a human-mouse chimeric mAb binding to human interleukin-6	IV	PS80	0.16 mg/mL
TANZEUM (albiglutide)	2014	a recombinant fusion protein comprised of 2 tandem copies of modified human glucagon-like peptide-1 genetically fused in tandem to human albumin, a glucagon-like peptide-1 ligand	SC	PS80	0.1 mg/mL
TRULICITY (dulaglutide)	2014	a fusion protein that consisting of 2 identical, disulfide-linked chains, each containing an N-terminal glucagon-like peptide-1 analog sequence covalently linked to the constant fragment of a modified human immunoglobulin G4 (IgG4) heavy chain by a small peptide linker, a glucagon-like peptide-1 antagonist	SC	PS80	0.2 mg/mL
VIMIZIM (elosulfase alfa)	2014	a purified human hydrolytic lysosomal glycosaminoglycan-specific enzyme hydrolyzing sulfate from either galactose-6-sulfate or N-acetyl-galactosamine-6-sulfate on the non-reducing ends of the glycosaminoglycans keratan sulfate and chondroitin-6-sulfate	IV	PS20	0.1 mg/mL
ADYNOVATE	2015	recombinant antihemophilic factor, PEGylated	IV	PS80	0.25 mg/mL (for 250, 500, 750, 1000, 1500 IU),<br><br>0.1 mg/mL (for 2000, 3000 IU)
COSENTYX® (secukinumab)	2015	a recombinant human mAb (IgG1κ) binding specifically to IL-17A	SC	PS80	0.2 mg/mL (prefilled syringe) 0.6 mg/mL (lyophilized powder)
DARZALEX® (daratumumab)	2015	A human mAb (IgG1κ) binding to CD38 antigen	IV	PS20	0.4 mg/mL
EMPLICITI™ (elotuzumab)	2015	a humanized recombinant monoclonal antibody directed to signaling lymphocytic activation molecule family member 7	IV	PS80	0.26 mg/mL

IXINITY®	2015	recombinant coagulation factor IX	IV	PS80	0.075 mg/mL
NUCALA (mepolizumab)	2015	a humanized recombinant mAb against interleukin-5	SC	PS80	0.67 mg/mL
PORTRAZZA (necitumumab)	2015	a recombinant human mAb (IgG1κ) binding to the ligand binding site of the human epidermal growth factor receptor	IV	PS80	0.1 mg/mL
PRALUENT® (alirocumab)	2015	a human mAb (IgG1) that targets proprotein convertase subtilisin kexin type 9	SC	PS20	0.1 mg/mL
PRAXBIND® (idarucizumab)	2015	humanized mAb fragment derived from an IgG1 isotype molecule, targeting the direct thrombin inhibitor dabigatra	IV	PS20	0.2 mg/mL
REPATHA (evolocumab)	2015	a human mAb (IgG2) directed against human proprotein convertase subtilisin kexin 9	SC	PS80	0.1 mg/mL
UNITUXIN™ (dinutuximab)	2015	a chimeric mAb composed of murine variable heavy and light chain regions and the human constant region for the heavy chain IgG1 and light chain κ, binding to the glycolipid disialoganglioside	IV	PS20	0.5 mg/mL
VONVENDI	2015	recombinant von Willebrand factor	IV	PS80	0.1 mg/mL
ZARXIO™ (filgrastim-sndz)	2015	a recombinant 175 amino acid human granulocyte colony-stimulating factor	SC, IV	PS80	0.04 mg/mL
AFSTYLA®	2016	recombinant antihemophilic factor, single chain	IV	PS80	0.2 mg/mL
ANTHIM® (obiltoxaximab)	2016	a chimeric mAb (IgG1κ) binding to the protective antigen component of B. anthracis toxin	IV	PS80	0.1 mg/mL
IDELVION®	2016	recombinant coagulation factor IX, albumin fusion protein	IV	PS80	Not available
INFLECTRA (infliximab-dyyb)	2016	a chimeric mAb (IgG1κ) composed of human constant and murine variable regions binding specifically to human tumor necrosis factor-alpha	IV	PS80	0.05 mg/mL
KOVALTRY	2016	recombinant antihemophilic factor	IV	PS80	80 ppm
TALTZ (ixekizumab)	2016	a humanized mAb (IgG4) with neutralizing interleukin-17A	SC	PS80	0.3 mg/mL
TECENTRIQ® (atezolizumab)	2016	an Fc-engineered, humanized, non-glycosylated Ab (IgG1κ) blocking the programmed cell death ligand 1	IV	PS20	0.4 mg/mL
ZINBRYTA (daclizumab)	2016	a humanized mAb binding to the alpha subunit of the interleukin-2 receptor (IL-2Rα, CD25)	SC	PS80	0.3 mg/mL
DUPIXENT® (dupilumab)	2017	a human mAb (IgG4 subclass) binding to the IL-4Rα subunit and inhibits IL-4 and IL-13 signaling	SC	PS80	2 mg/mL
KEVZARA (sarilumab)	2017	a human recombinant mAb (IgG1) binding to the IL-6 receptor	SC	PS20	2 mg/mL
RITUXAN HYCELA™ (rituximab and hyaluronidase human)	2017	a combination of rituximab (chimeric murine/human mAb (IgG1κ) against the CD20 antigen) and hyaluronidase human	SC	PS80	0.6 mg/mL
SILIQ™ (brodalumab)	2017	a human mAb (IgG2κ) against human interleukin-17 receptor A	SC	PS20	0.1 mg/mL
TREMFYA® (guselkumab)	2017	a human immunoglobulin G1 lambda (IgG1λ) mAb blocking interleukin-23	SC	PS80	0.5 mg/mL
AIMOVIG® (erenumab-aooe)	2018	a human mAb (IgG2) monoclonal antibody with high affinity binding to the calcitonin gene-related peptide receptor	SC	PS80	0.1 mg/mL

AJOVY™ (fremanezumab-vfrm)	2018	a fully humanized mAb (IgG2Δa/κ) specific for calcitonin gene-related peptide ligand	SC	PS80	0.2 mg/mL
EMGALITY (galcanezumab-gnlm)	2018	a humanized mAb (IgG4) specific for calcitonin-gene related peptide ligand	SC	PS80	0.5 mg/mL
ILUMYA™ (tildrakizumab-asmn)	2018	a humanized IgG1 IgGκ Ab binding to the p19 subunit of interleukin-23	SC	PS80	0.5 mg/mL
TAKHZYRO™ (lanadelumab-flyo)	2018	a non-plasma derived, recombinant, fully human mAb (IgG1κ - light chain) binding to plasma kallikrein and inhibiting its proteolytic activity	SC	PS80	0.1 mg/mL
TROGARZO™ (ibalizumab-uiyk)	2018	a humanized mAb (IgG4) directed against CD4 domain 2	IV	PS80	0.45 mg/mL
BEOVU® (brolucizumab-dbll)	2019	a recombinant human monoclonal single-chain variable AB fragment acting as a vascular endothelial growth factor inhibitor	IVT	PS80	0.2 mg/mL
HERCEPTIN HYLECTA™ (trastuzumab and hyaluronidase-oysk)	2019	a combination of trastuzumab (humanized mAb (IgG1κ) with high affinity to the extracellular domain of the human epidermal growth factor receptor 2 protein) and hyaluronidase	SC	PS20	0.4 mg/mL
SKYRIZI® (risankizumab-rzaa)	2019	a humanized mAb (IgG1) against interleukin-23	SC	PS20	0.2 mg/mL
PHESGO (pertuzumab, trastuzumab, and hyaluronidase-zzxf)	2020	a combination of pertuzumab (a recombinant humanized mAb targeting subdomain II of the human epidermal growth factor receptor 2 protein), trastuzumab (humanized mAB (IgG1κ) with high affinity to the extracellular domain of the human epidermal growth factor receptor 2 protein) and hyaluronidase	SC	PS20	0.4 mg/mL
VYEPTI™ (eptinezumab-jjmr)	2020	a humanized mAb (IgG1) specific for calcitonin gene-related peptide ligand	IV	PS80	0.15 mg/mL
ADUHELM™ (aducanumab-avwa)	2021	a recombinant human mAB (IgG1) against aggregated soluble and insoluble forms of amyloid beta	IV	PS80	0.5 mg/mL
DARZALEX FASPRO® (daratumumab and hyaluronidase-fihj)	2021	Combination of daratumumab (a human mAb (IgG1κ) binding to the CD38 antigen) and hyaluronidase	SC	PS20	0.4 mg/mL
EVKEEZA™ (evinacumab-dgnb)	2021	A recombinant mAb (IgG4) inhibiting angiopoietin-like protein 3	IV	PS80	1 mg/mL
SUSVIMO™ (ranibizumab injection)	2021	a recombinant humanized mAb fragment (IgG1κ) binding to and inhibiting human vascular endothelial growth factor-A	IVT	PS20	0.01 mg/mL

Abbreviations: CD: cluster of differentiation, IgG: Immunoglobulin G, IV: intravenous, IVT: intravitreal, mAB: monoclonal antibody, PS20: polysorbate 20, PS80: polysorbate 80, SC: subcutaneous
